# Supplementary material for: Bicoid gradient formation mechanism and dynamics revealed by protein lifetime analysis
Source: Mol Syst Biol. 2018 Sep 4;14(9):e8355. doi: 10.15252/msb.20188355 (PMC6121778; doi:10.15252/msb.20188355)
Supplement: Supplementary file 1 — Appendix [file MSB-14-e8355-s001.pdf]

Appendix for

Bicoid gradient formation mechanism and dynamics revealed by protein lifetime analysis

Lucia Durrieu[1,2,6], Daniel Kirrmaier[1,3], Tatjana Schneidt[2], Ilia Kats[1], Sarada Raghavan[1,7], Lars Hufnagel[2,*], Timothy E Saunders[2,4,5,*] and Michael Knop[1,3,*]

[1] Zentrum für Molekulare Biologie der Universität Heidelberg (ZMBH), University of Heidelberg, Im Neuenheimer Feld 282, 69120 Heidelberg, Germany.
[2] European Molecular Biology Laboratory (EMBL), Meyerhofstraße 1, 69117 Heidelberg, Germany.
[3] Deutsches Krebsforschungszentrum (DKFZ), Im Neuenheimer Feld 280, 69120 Heidelberg, Germany.
[4] Mechanobiology Institute and Department of Biological Sciences, National University of Singapore 117411, Singapore
[5] Institute of Molecular and Cell Biology, A*Star, Biopolis 138673, Singapore
[6] Current address: Instituto Leloir, Av. Patricias Argentinas 435, C1405BWE Ciudad de Buenos Aires, Argentina; Departamento de Fisiología, Biología Molecular, y Celular, Facultad de Ciencias Exactas y Naturales, Universidad de Buenos Aires, Buenos Aires, Argentina.
[7] Current address: p53 Laboratory, A*STAR, 8A Biomedical Grove, #06-06 Immunos, Singapore 138648
[*] For correspondence: m.knop@zmbh.uni-heidelberg.de, dbsste@nus.edu.sg, hufnagel@embl.de

Appendix Index:

Box 1: Outline of models considered
Box 2: Outline of protein age dynamics in different models

A. Imaging and image analysis
- A.1 MuVi SPIM sensitivity and accuracy
- A.2 Stereographic projection
- A.3 Segmentation
- A.4 Gradient fitting protocol
- A.5 Timer normalization protocol
- A.6 Image inhomogeneity correction

B. Theoretical modeling and simulation
- B.1 Models of Bcd gradient formation
- B.2 Predictions of average protein lifetime
- B.3 Timer ratio without protein production
- B.4 Simulations of the alternative models for Bcd gradient formation
- B.5 Simulations of the gradient dynamics before equilibrium

C. Appendix tables

D. Determination of the maturation kinetics of the fluorescent proteins
- D.1 RNA injection of embryos
- D.2 Imaging and image analysis
- D.3 Modelling of maturation rates and data fitting

E. Appendix References

F. Appendix figures and legends

Box 1: Summary of models of Bicoid protein concentration gradient formation

Synthesis, diffusion, degradation (SDD) model[1,2]:
Bcd is synthesized at rate *J* in the anterior pole, before diffusing (*D*) and degrading at rate *μ*. The Bcd concentration (C_{Bcd}) is then given by the reaction-diffusion equation

$$\frac{\partial C_{Bcd}}{\partial t} = D\frac{\partial^2 C_{Bcd}}{\partial x^2} - \mu C_{Bcd} + J\delta(x) \quad \text{(E1)}$$

In steady-state, the solution of Eq. E1 is given by

$$C_{bcd}^{ss} = \frac{J\lambda}{D} e^{-x/\lambda}, \quad \text{(E2)}$$

where $\lambda = \sqrt{D/\mu}$ and $\lambda << L$ (where L is the system length). It is also possible that the gradient may not reach dynamic equilibrium within the 2-3 hour time window of early development, resulting in *pre-steady-state* interpretation [3,4].

Nuclear shuttling model[5]:
This model is similar to the SDD model except *μ=0* in Eq. E1 and Bcd nuclear protein is diluted by increased trapping upon nuclei division. The length scale is now time dependent $\lambda_{shut}(t) = \sqrt{4Dt}$ and there is no steady-state (a secondary Bcd degradation pathway is required to clear Bcd during cycle 14). By suitable choice of *D*, the profile is exponential-like in early n.c. 14, Figure 1C and Appendix Figure S1B.

Spatially distributed bcd mRNA model[6-8]:
Here, we consider the *bcd* mRNA gradient to be exponentially-distributed with length scale λ_{rna} though alternative distributions can be used[9]. This alters the synthesis term in Eq. E1 to $\frac{J}{\lambda_{rna}} e^{-x/\lambda_{rna}}$. In the limit $\lambda_{rna} << \lambda$ the steady-state solution reduces to Eq. E2 while in the limit $\lambda_{rna} >> \lambda$ then the steady-state Bcd distribution is effectively described by the underlying *bcd* mRNA distribution. Note that other suitable distributions for the mRNA gradient do not significantly alter our results[7].

bcd mRNA diffusion and degradation model[6,10]:
The dynamics that describe this model are similar to the SDD model (Eq. 1) but now we consider *bcd* mRNA (c_{bcd}), not Bcd protein, diffusion. There is fixed initial *bcd* mRNA number in the anterior with (relatively) static Bcd protein:

$$\frac{\partial c_{bcd}(x,t)}{\partial t} = D_{bcd}\frac{\partial^2 c_{bcd}(x,t)}{\partial x^2} - \mu_{bcd} c_{bcd}(x,t) + N_{bcd}\delta(x)\delta(t), \qquad \frac{\partial C_{Bcd}(x,t)}{\partial t} = \alpha c_{bcd}(x,t) \quad \text{(E3)}$$

Although there is no Bcd protein degradation, this model obtains a steady state as the Bcd production ceases when the initial *bcd* mRNA is fully degraded.

Adaptations to these models, such as incorporating both distributed *bcd* mRNA and time dependent diffusion have been analyzed [11], including advection [12]. See *Appendix: **Theoretical modeling and Simulation*** for more complete mathematical details of the above models.

Box 2: Calculation of average Bcd age using different models

Here, we briefly outline qualitative arguments for the expected behavior of the four models considered. See ***Theoretical modeling and simulation*** for more complete mathematical details.

The average protein age depends on the mechanism of protein production and (possible) protein degradation and diffusion. For the SDD model, in steady state the average protein age $\tau(x)$ at a position *x* is given

$$\tau_{sdd} = \frac{1}{2\mu}\left(1+\frac{x}{\lambda}\right) \qquad \text{if } \lambda << L \text{ (where } L \text{ is system length)} \qquad \text{(E4)}$$

Interestingly, this is the same as the timescale to reach steady-state in the SDD model[13].

For the nuclear shuttling model, the protein age scales with system age, as there is no degradation. The protein age at larger distances from the source is older as it typically takes a time ~ x^2/D to diffuse a distance *x* (though the boundaries complicate the form of $\tau_{NucSh}(x,t)$, *Appendix: Theoretical modeling and simulation*).

For the RNA gradient model, Bcd is produced more locally. Therefore, there is reduced change in $\tau(x)$ as a function of position. When the *bcd* RNA gradient extends further than the diffusion length scale ($\lambda_{rna} >> \lambda$) then the average protein age is approximately constant throughout the embryo. In steady-state, the protein age is given by (again, analogous to the time to steady-state [9])

$$\tau_{RNA-grad} = \tau_{sdd}\left(\frac{1}{1-\Lambda e^{-x(1-\Lambda)/\lambda_{rna}}} + \frac{2}{(1-\Lambda^{-1})^2(1+x/\lambda)}\right) \text{ where } \Lambda = \lambda_s/\lambda \qquad \text{(E5)}$$

In the extreme case that $\lambda_{rna} = \lambda$ then protein is produced locally and diffusion can be set to zero yet still replicate the observed Bcd gradient. In this case, the protein has the same average age throughout the embryo, determined by the protein lifetime.

For the RNA diffusion model the average protein age gets younger as a function of distance from the anterior pole. This is because protein is first produced at the anterior pole where *bcd* mRNA is initially localized. The RNA then diffuses through the embryo, with new Bcd protein produced at steadily further distances from the anterior. The *bcd* dynamics mirror those of the SDD model for Bcd, resulting in an average protein lifetime given by (at long times, after all *bcd* RNA has degraded)

$$\tau_{RNA-diff} = t - \frac{1}{2\mu_{rna}}\left(1+\frac{x}{\sqrt{D_{rna}/\mu_{rna}}}\right). \qquad \text{(E6)}$$

See *Appendix: Theoretical modeling and simulation* for more complete discussion of the protein age in the models, including pre-steady-state solutions for $\tau(x)$ and implementation of boundary conditions.

A. Imaging and Image Analysis

A.1 MuVi-SPIM Characterization for imaging of *Drosophila* embryogenesis

We used light-sheet fluorescent microscopy (LSFM) to gain rapid *in toto* 4D images *in vivo* with subcellular resolution and low photo-bleaching. To characterize our microscope (MuVi-SPIM) we first explored the developing embryo expressing Bcd-Venus, Appendix Figure S5. To validate the microscope sensitivity, we focused on two challenging regions. First, we imaged Bcd-Venus expression in cycle 8 of embryo development, before the nuclei reached the outer membrane, Appendix Figure S5G. Nuclear Bcd-Venus expression was clear, despite the presence of lipids around the nuclei. Second, we analyzed Bcd-Venus expression in the posterior pole of the embryo in early cycle 14, Appendix Figure S4h. Although weak, expression above background levels was clearly observable in even the most posterior nuclei, Appendix Figure S5H. Background subtraction was performed through linear unmixing, as described in Methods and Appendix Figure S13.

We quantified the signal uniformity across the embryo by imaging H2b-mCherry expressing embryos, Appendix Figure S5I, and OregonR embryos, Appendix Figure S5J. The measured intensity was relatively flat across the embryo. The slight drop in H2b-mCherry intensity at the poles was partially due to difficulties in segmentation.

The relative fluctuations in the nuclear Bcd signal, Appendix Figure S5K-L, were comparable to previous published work[14]. Therefore, the readout of the Bcd gradient in MuVi-SPIM was reliable and comparable with previous observations using two-photon confocal microscopy.

A.2 Stereographic projection

During n.c. 9-14 of *Drosophila* embryo development Bcd is localized to the cortical region of the embryos. Therefore, we performed a stereographic projection of the embryo cortical surface[15,16], Appendix Figure S5A-B. With such a 2D projection, image segmentation was straightforward and we could extract the Bcd-eGFP intensity profile across the whole embryo, Appendix Figure S5C. We confirmed the projection accuracy by mapping the coordinates back to the three-dimensional data, Appendix Figure S5D-E. Finally, we look at the Bcd gradient on the dorsal and ventral sides of the embryo. Consistent with previous work[17], the dorsal and ventral gradients were equivalent after correction for source offset, Appendix Figure S5F.

The key ideas behind the stereographic projection used in the analysis are provided elsewhere[15,18]. The main difference between the projection performed previously (which was based on either histone or membrane localized signals) and here is that Bcd is spatially varying in concentration and therefore the Bcd fluorescent signal cannot be used to segment the whole embryo. Instead, we implemented in Matlab a manual segmentation protocol. The basic protocol was as follows:

1) Image aligned along the AP axis of the embryo
2) User determines planes describing most anterior/posterior parts of embryo

3) User inputs number of desired intermediate planes to segment
4) User then presented with each selected plane and they draw a polygon (using Matlab function *roipoly*) around the embryo
5) The software then interpolates the segmentation to the other planes
6) After segmentation, the same protocol as outlined in [15] was followed to extract the "carpet" of Bcd intensity
7) The 3D to 2D mapping was also inverted to ensure that the carpet faithfully reproduced the 3D data, Appendix Figure S5D-E.

A.3 Segmentation

Segmentation was performed using Ilastik (ilastik.org,[19]). This machine-learning approach could reliably segment the 2D carpets quickly. Though much slower, we also confirmed that we could segment the full 3D data on a PC with 128Gb RAM using Ilastik. Analysis of the segmentation data was then done in Matlab using custom written code.

A.4 Gradient fitting protocol

The Bcd gradient has an exponentially-decaying profile away from the anterior pole [2,20], Appendix Figure S5C. In model fitting, particularly of exponential-like profiles, reliable fitting can only be achieved by a fit that spans a range of magnitudes. We were able to fit an exponentially-decreasing profile, $I = I_0 e^{-x/\lambda}$, to the Bcd-eGFP gradient (where λ is the decay length) across almost the entire embryo length (50μm to 450μm), representing around four decay lengths, Appendix Figure S5C. Fitting to an exponential profile was performed using the Matlab function *fit*, utilizing a least-squares algorithm and included the 95% confidence intervals on each fit. In all embryos, the fitted decay length λ was between 100-110 μm in early cycle 14. Due to the large amount of data in a single time point, we could fit the Bcd profile of a single embryo with high confidence (typical 95% confidence intervals are within 2% of the best fit value). For the tandem reporter, the signal was less bright and so we were typically limited to ~80% of the embryo. However, this was more than sufficient for reliable curve fitting. We obtained similar curves for different Bcd insertions (Venus, eGFP and mCherry-sfGFP) in early n.c. 14. Overall, we analyzed the Bcd gradient across almost its entire extent and hence could reliably deduce the decay length with high confidence for a single embryo.

A.5 Timer normalization protocol

After background subtraction the mCherry and sfGFP profiles need to be compared. One approach is to use *a priori* knowledge that only *old* protein reaches posterior regions of the embryo (true if the SDD model is correct) and hence it is likely that both fluorophores will have folded. However, there are a number of issues with this approach. (1) If there is a significant FRET effect between the mCherry and sfGFP then the final ratio between the signals should not be 1 but offset from this. (2) Although both fluorophores will have mostly folded, there will still be a higher fraction of sfGFP folded than mCherry. For example, assuming that folding is a Poisson process then the probability of a fluorophore having folded by time *t* is given by

$P_{fold}(t) = 1 - e^{-t/\tau}$ where τ is the characteristic fluorophore folding time. In the SDD model the average age of a protein a distance *x* from the source in steady-state is given by $t_{age} = \frac{1}{2\mu}\left(1 + \frac{x}{\lambda}\right)$ (Eq. E4) as discussed in Box 2. Therefore, at x=300μm with λ=85μm we have an average protein age of around 100 minutes (with μ=1/45 min^{-1}). Using τ=18 minutes (sfGFP) and τ=50 minutes (mCherry, ignoring two step folding process here) suggests that in the normalization region, 99% of sfGFP will have folded but only around 90% of mCherry has folded. Therefore, the normalization should not be set to 1 in the region 250-350 μm but instead to around 0.9. Of course, here *a priori* knowledge was used to determine the normalization protocol. So, we do not use this approach but instead introduced an additional fitting parameter ε representing our uncertainty in the fluorescence scaling. In Appendix Figure S5M we show that measurements of the mCherry-sfGFP-Bcd fluorescence ratio on different microscopes (confocal, light-sheet) and with different imaging conditions gives similar profile shape after scaling only by a multiplicative factor.

In the case of diffusive *bcd* mRNA with localized Bcd protein (i.e. protein non-diffusive) we would expect to see the opposite trend to that observed: older protein near the anterior pole and younger protein further away. Therefore, we normalized the profiles with respect to the anterior pole instead, Appendix Figure S5N. Again, the general shape is unchanged with increasing protein age as a function of distance. This suggests that *bcd* mRNA diffusion is not the primary mechanism of Bcd gradient formation (this does not discount such a mechanism completely but it is unlikely to be the primary mechanism underlying Bcd gradient formation).

The fitting to the tFT-ratio was done using an additional free parameter ε representing the intensity scaling between the two different fluorophores. This fitting parameter increased the error in the fits, Figure 3D and Appendix Figure S7. However, the final parameter chosen was the one that minimized both the fit to the mCherry/sfGFP ratio and the intensity profiles. Fitting was performed by minimizing $r^2 = \frac{1}{N\langle O\rangle}\sum_{i=1}^{N}(E_i - O_i)^2$ for the sfGFP and tandem ratios simultaneously. The normalization N<O> ensures that the relative contributions to the fitting from the sfGFP profile and the tandem ratios are balanced. We also checked that using $\chi^2 = \sum_i \frac{(E_i - O_i)^2}{E_i}$ where E_i and O_i represented the model prediction and experimental observation for each position x_i, did not alter our general conclusions.

A.6 Image inhomogeneity correction

The measured image intensity $I_{measured}(x,y,z)$ is a function of position due to imaging inhomogeneity. Such inhomogeneity typically appears as a multiplicative factor $K(x,y,z)$ in the image intensity as the variability is typically due to changes in light intensity at different positions. Therefore, accounting for background $I_{background}(x,y,z)$,

$$I_{measured}(x,y,z) = K(x,y,z)I_{actual}(x,y,z) + I_{background}(x,y,z) \quad \text{(E7)}$$

Using linear unmixing we can (approximately) remove $I_{background}(x,y,z)$. If $K(x,y,z)$ is dependent primarily on variations in illumination intensity then it is (to a first approximation) independent of the illumination wavelength. Therefore $K(x,y,z)$ is similar for both 488nm and 561nm illumination. Hence,

$$\frac{I^{sfGFP}_{measured}(x,y,z) - I^{sfGFP}_{background}(x,y,z)}{I^{mCherry}_{measured}(x,y,z) - I^{mCherry}_{background}(x,y,z)} = \frac{I^{sfGFP}_{actual}(x,y,z)}{I^{mCherry}_{actual...}(x,y,z)} \quad \text{(E8)}$$

which is independent of $K(x,y,z)$. Therefore, although illumination inhomogeneity affects measurements of, for example, the Bcd spatial concentration profile, it does not affect the measurement of the timer ratio.

B. Theoretical modeling and simulations

B.1 Models of Bcd gradient formation

B.1.1. Synthesis, Diffusion, Degradation (SDD) Model

In the SDD model, the Bcd protein is synthesized (at rate *J*) in the anterior region. The Bcd protein then diffuses (D) through the cytoplasmic space, constrained to be near the cortex before degrading (μ). The spatio-temporal dynamics of the Bcd concentration (C_{bcd}) are then described by (in one dimension)

$$\frac{\partial C_{Bcd}}{\partial t} = D\frac{\partial^2 C_{Bcd}}{\partial x^2} - \mu C_{Bcd} + J\delta(x) . \qquad \text{(E9)}$$

The general solution of Eq. E9 at time *t* and position *x* is given by

$$C_{Bcd} = \frac{J\lambda}{D}\left(e^{-x/\lambda} - \frac{1}{2}e^{-x/\lambda} erfc\left(\frac{\frac{2Dt}{\lambda} - x}{\sqrt{4Dt}}\right) - \frac{1}{2}e^{x/\lambda} erfc\left(\frac{\frac{2Dt}{\lambda} + x}{\sqrt{4Dt}}\right)\right) \qquad \text{(E10)}$$

where $\lambda = \sqrt{D/\mu}$ and $\lambda << L$ (where *L* is the system length) so that the right hand boundary is effectively unimportant. In steady-state, Eq. E10 reduces to

$$C_{bcd}^{ss} = \frac{J\lambda}{D}e^{-x/\lambda} . \qquad \text{(E11)}$$

When analyzing the data in the paper, we used Eq. E10 to describe the behavior of the Bcd morphogen gradient.

Our approach considered a one-spatial-dimensional model of Bcd gradient formation (for all models). This is a reasonable approximation to the Bcd gradient[2] but higher dimensional models that account more realistically for the embryo morphology have also been used[3,11,21]. Therefore, our estimations of the Bcd dynamic parameters may be altered by extending the model to higher dimensions, though the consistency between the one-dimensional model and more complex implementations means that we are confident that our approach is a reasonable approximation to the underlying dynamics.

B.1.2. Shuttling Model

In the shuttling model, the Bcd protein is again synthesized at the anterior and diffuses through the embryo[5,7]. However, there is no Bcd degradation. The parameters are selected such that the nuclear divisions act to dilute the Bcd signal to ensure relative constant Bcd concentration in nuclei during n.c. 12-14. It is then assumed within this model that in n.c. 14 an alternative mechanism acts to degrade the Bcd protein. For the total Bcd concentration, the spatio-temporal dynamics are described by

$$\frac{\partial C_{Bcd}}{\partial t} = D\frac{\partial^2 C_{Bcd}}{\partial x^2} + J\delta(x) \qquad \text{(E12)}$$

with general solution

$$C_{Bcd} = \frac{J\lambda(t)}{D}\left(\frac{1}{\sqrt{\pi}}e^{-\frac{x^2}{\lambda(t)^2}} - \frac{x}{\lambda(t)}erfc\left(\frac{x}{\lambda(t)}\right)\right) \qquad \text{(E13)}$$

where $\lambda(t) = \sqrt{4Dt}$ is now the time-dependent length scale of the gradient. The nuclear Bcd concentration is then determined by the shuttling rate into nuclei[7]. There is no steady-state in the shuttling model.

B.1.3. RNA Gradient Model

The RNA gradient model is similar dynamically to the SDD model but now, instead of the Bcd originating from a point source in the anterior, the *bcd* mRNA is distributed, resulting in non-local production of Bcd[6,8,11]. The shape of the mRNA distribution is typically taken to be a gradient akin to the Bcd protein gradient. Here, we consider the case when the *bcd* mRNA is exponentially distributed with length scale λ_{rna}. Alternatives, such as Gaussian distributed sources, are also viable[6,7] but do not significantly alter our general conclusions discussed.

We consider the *bcd* mRNA gradient to be exponentially-distributed,

$$c_{bcd} = \frac{J}{\lambda_{rna}}e^{-x/\lambda_{rna}}. \qquad \text{(E14)}$$

The steady-state solution of this model is given by[9]

$$C_{Bcd}^{ss} = \frac{J\lambda}{D}\frac{1}{(1-\Lambda^2)}\left(e^{-x/\lambda} - \Lambda e^{-x/\lambda_{rna}}\right) \qquad \text{(E15)}$$

where $\Lambda = \lambda_{rna}/\lambda$. In the limit $\lambda >> \lambda_{rna}$ then the solution reduces to Eq. E11. In the alternative limit $\lambda_{rna} >> \lambda$ then the Bcd distribution is effectively described by the underlying *bcd* mRNA distribution.

B.1.4. *bcd* RNA Diffusion Model

This model assumes that the *bcd* RNA (c_{rna}) is the primary dynamic mechanism and that Bcd protein is relatively stationary[10]. In this case, a pool of *bcd* mRNA is deposited in the anterior pole by the mother. This pool of RNA then diffuses (D_{rna}) through the embryo and degrades (μ_{rna}). Bcd is produced locally, at a rate α. Such dynamics are described by the coupled equations:

$$\frac{\partial c_{rna}}{\partial t} = D_{rna}\frac{\partial^2 c_{rna}}{\partial x^2} - \mu_{rna}c_{rna} \qquad \text{(E16)}$$

$$\frac{\partial C_{Bcd}}{\partial t} = \alpha c_{rna} \qquad \text{(E17)}$$

where initially c_{rna} is localized to the anterior pole. The particular initial distribution of c_{rna} can be varied. Here, we consider all the c_{rna} localized at $x=0$. We note that more complex distributions[10] give slightly improved fits to the Bcd gradient but do not alter the underlying dynamic properties.

For initial condition $c_{rna}(x,0) = \frac{A}{\lambda_s}\delta(x)\delta(t)$ the solution of Eqs. E16-17 are

$$c_{rna}(x,t) = \frac{A}{\sqrt{\pi D t}} e^{-\mu t} e^{-\frac{x^2}{\lambda(t)^2}} \quad \text{(E18)}$$

$$C_{Bcd}(x,t) = \frac{\alpha A}{2\lambda_s}\left[erfc\left(\frac{\frac{x}{\lambda_s} - 2\mu t}{\sqrt{4\mu t}}\right) e^{-x/\lambda_s} - erfc\left(\frac{\frac{x}{\lambda_s} + 2\mu t}{\sqrt{4\mu t}}\right) e^{x/\lambda_s} \right] \quad \text{(E19)}$$

where $\lambda_s = \sqrt{D_{rna} / \mu_{rna}}$ and $\lambda(t) = \sqrt{4Dt}$. In steady-state ($t \to \infty$) we obtain

$$C_{Bcd}(x) = \frac{\alpha A}{\lambda_s} e^{-x/\lambda_s}. \quad \text{(E20)}$$

Therefore, we see that all models considered can create an exponentially-decaying profile with (effective) length scale of around 100 μm at particular times with plausible parameter selection. We emphasize here that we generally use the simplest form of each model to test the dynamic behavior: our objective is to discover which model best replicates the observed protein age, not find a detailed model exactly replicating the Bcd morphogen gradient. Hence, for example, we do not consider the more complex initial conditions used in Ref. [10] as the fundamental protein dynamics are unchanged.

B.2 Theoretical predictions for average protein lifetime

When considering the protein age, the models fall into two general categories: those with protein degradation; and those without protein degradation. In the latter case, in the long time limit the average protein age scales with the system age. The shuttling and RNA diffusion (even though it obtains a steady-state) models fall into this latter category. To give the reader a more intuitive understanding of protein age we begin by outlining the results for a simple model without diffusion where we can analytically compare different modes of concentration dynamics. We then discuss the models of Bcd gradient formation.

B.2.1 Simple model: same concentration, different protein lifetimes

Consider the following two models describing the temporal change in concentration of some protein. Model A has constant production *J* and linear protein degradation m. Model B has no degradation but production is temporally dependent, and decays as exp(-μt). Both models start initially with $\rho(t=0)=0$. Mathematically, the two models can be described by the equations: (A) $\dot{\rho} = J - \mu\rho$; and (B) $\dot{\rho} = Je^{-\mu t}$. Both models have identical solution for the concentration $\rho = J(1-e^{-\mu t})/\mu$, Theory Figure 1a. However, the predicted protein lifetime is very different.

For Model A, the probability of a protein being τ old is $P(\tau) = \mu e^{-\mu\tau}$ (*i.e.* the probability it has not decayed up to time τ). However, we need to account for the system age, *t* (i.e. a protein cannot be τ old if t < τ). Therefore, we are interested in the probability that a protein is τ old given the system is time *t* old,

$$P(\tau \mid t) = \frac{\mu e^{-\mu\tau}}{1-e^{-\mu t}} \tag{E21}$$

Hence, we can calculate the average particle age, $<\tau(t)>$, at time t,

$$<\tau(t)> = \int_0^t \frac{\tau' \mu e^{-\mu\tau'}}{1-e^{-\mu t}} d\tau' = \frac{1}{\mu}\left[1-\frac{\mu t e^{-\mu t}}{1-e^{-\mu t}}\right] \tag{E22}$$

At large times this reduces to $<\tau> = \mu^{-1}$ as expected. At very short times ($\mu t << 1$), $<\tau(t)> \sim t/2$ since little degradation has occurred.

In Model B, there is no degradation but production is not constant. Therefore, the probability of a particle being τ old is given by the probability that is was produced at time t- τ:

$$P(\tau \mid t) = \frac{\mu e^{-\mu(t-\tau)}}{1-e^{-\mu t}} \tag{E23}$$

and hence

$$<\tau(t)> = \frac{\mu e^{-\mu t}}{1-e^{-\mu t}} \int_0^t \tau' e^{\mu\tau'} d\tau' = \frac{t-\frac{1}{\mu}(1-e^{-\mu t})}{1-e^{-\mu t}} . \tag{E24}$$

In the limit of long time this tends to $<\tau(t)> = t-\mu^{-1}$ (*i.e.* the system time minus the average time that a protein was produced) and at very short times ($\mu t << 1$), $<\tau(t)> \sim t/2$ (as with Model A). Therefore, these toy models allow us to see that measuring protein lifetime can distinguish models that have identical concentration behavior, Theory Figure 1b.

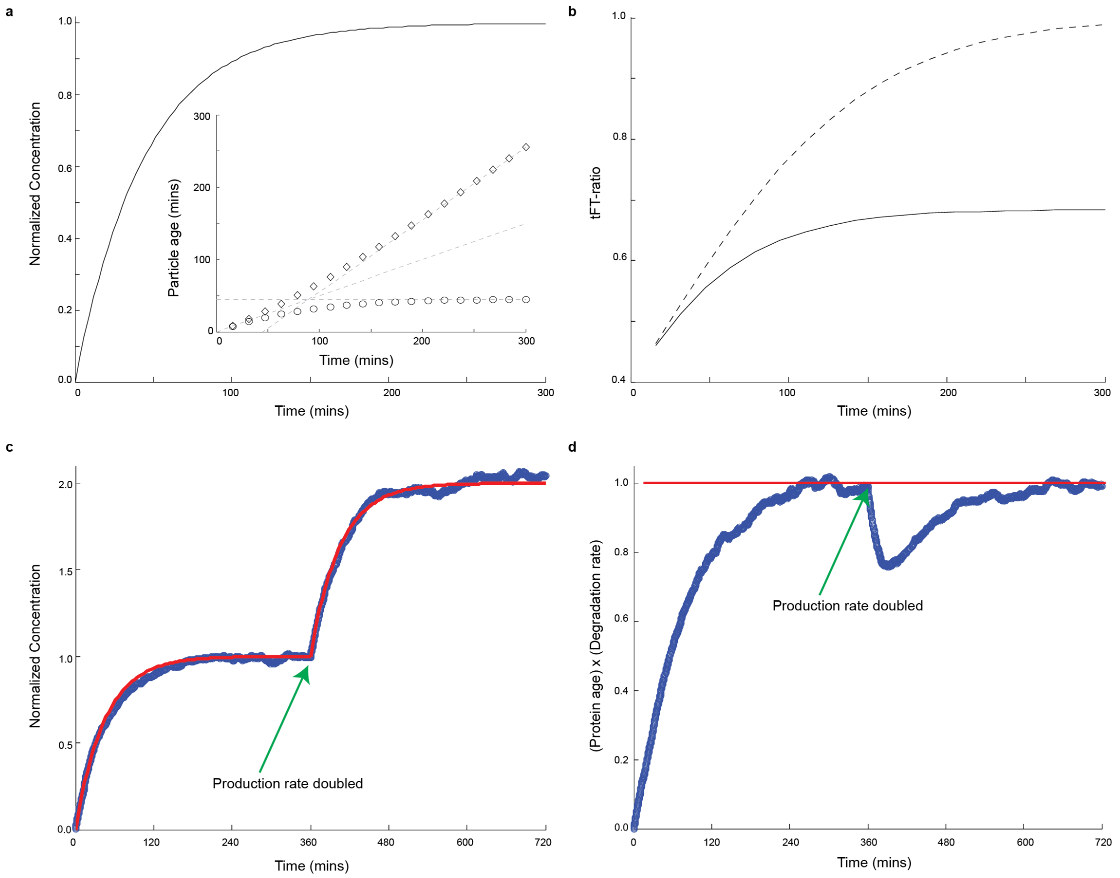

Theory Figure 1: Comparison of protein lifetime in two toy models with identical protein concentration behavior (see Appendix: Theory). **a** Both models have identical concentration time dependence but the protein lifetimes in the two models are different (inset), with circles representing model A which has degradation and diamonds model B which has limited protein synthesis. **b** Corresponding mCherry/sfGFP ratio for both models (solid line, model A; dashed line, model B). **c** In model A, the system is allowed to reach steady-state, and then synthesis is double (green arrow). **d** The corresponding effect on average protein age in the system (scaled by degradation rate). At the change in production, the average protein age transiently decreases.

Further, using this toy model it is straightforward to see that the production rate itself does not affect protein age (so long as there are no non-linear interactions in the system). In Theory Figure 1c-d, we show the scenario where model (A) has its production rate doubled after some time. The total concentration eventually doubles. However, the average protein age returns to μ^{-1} after a brief dip. This dip is caused by the sudden increase in very young protein due to the increase in production rate – *i.e.* the protein age is sensitive to $\frac{\partial J}{\partial t}$ but not *J* itself. Therefore, we see that the protein age can be sensitive to temporal shifts in production, but it is insensitive to the underlying production rate value.

B.2.2 Synthesis, Diffusion, Degradation (SDD) Model

In the SDD model, since there is continuous production (exceptions to this discussed below) and degradation the proteins have a finite average lifetime in steady-state. At small times ($t \ll 1/\mu$) there are relative few degradation events and hence protein age scales with system time. However, as more degradation occurs the average protein age becomes less dependent on system time (essentially, the system "forgets" its initial condition). In steady-state, the average protein age as a function of distance is given by

$$<\tau(x)>=\frac{\int_0^\infty t'\dot{\rho}(x,t')dt'}{\rho_{ss}(x)} \qquad \text{(E25)}$$

which reduces to at long times

$$\tau(x)=\frac{1}{2\mu}\left(1+\frac{x}{\lambda}\right), \qquad \text{(E26)}$$

in the limit of $L \gg \lambda$ (where *L* is the system length). Note that this is also the time scale for the gradient to obtain steady-state[9]. In systems that have protein degradation, the time scale to obtain steady state at position *x* and the average age of particles in steady state at that position are the same. Extending this model to a finite system (*i.e.* where the boundary at *x*=*L* must be considered) complicates the form of Eq. E25 but is straightforward to compute numerically.

To derive the pre-steady-state time distribution is more challenging. Essentially, we need to calculate $P(\tau|x)_t$ - the probability that a particle is age τ at a distance *x* from the source after total time *t.* We consider the behavior of a single particle.
From diffusion theory we know $P(x|\tau)_t$ as this is simply the expected diffusion of the particle injected at time *t-t*: $P(x|\tau)=\frac{2e^{-x^2/\lambda(\tau)^2}}{\sqrt{\pi}\lambda(\tau)}$ (i.e. Gaussian distributed from the source with infinite system size – see below for finite size solution). The probability of the particle (assuming linear decay) being age τ is $P(\tau)=m\exp[-\mu t]$ (note that both

these terms are independent of system age as we consider a single particle). Finally, in steady-state the probability of a particle being at position *x* is given by $P(x)=\frac{1}{\lambda}e^{-x/\lambda}$. Therefore, we can use Bayes formula to find $P(\tau|x)$

$$P(t\,|\,x)=\frac{P(x\,|\,t)P(t)}{P(x)}=\sqrt{\frac{\mu}{\pi}}\frac{e^{-\frac{x^2}{\lambda(t)^2}}e^{-\mu t}}{\sqrt{t}e^{-x/\lambda}} \tag{E27}$$

and hence

$$\tau(x)_t=\int_0^t tP(t\,|\,x)dt \tag{E28}$$

In the steady-state limit this reduces to Eq E26. In Figure 1C and Appendix Figure S1 we see good agreement between the simulations and Eq. 26 at long times where we solve Eq. E28 numerically, accounting for finite boundary conditions. In Figure 1C, we solve the full pre-steady-state equations, where *P(x)* is now the spatial distribution at time *t*, (not necessarily at steady-state). Solving this analytically results in an unwieldy solution (particularly if finite system size is considered) so we numerically integrate Eq. E28 (using Matlab).

B.2.3. Shuttling Model

The shuttling model does not obtain a steady state. The age of a protein is $\tau=t-t_{prod}$, where *t* is the system age and t_{prod} the time of production. Since diffusion is the driving dynamics, a protein of average age τ will have travelled an average distance $x=\sqrt{2D\tau}$. At a given position x, if the time is greater than approximately $\tau=x^2/4D$ (or equivalently one could consider the mean-first-passage-time) then the age of protein will scale with position. Of course, τ cannot be greater than *t,* so at large times the the protein lifetime is determined by the total time *t*, as can be seen in Figure 1D and Appendix Figure S1C-D.

The probability of a particle being at position x given a particle age t, in a system of length *L:*

$$P(x\,|\,t)=\frac{1}{L}\left(1+2\sum_{n=1}^{\infty}\cos\left(\frac{n\pi x}{L}\right)e^{-\left(\frac{n\pi}{L}\right)^2 Dt}\right) \tag{E29}$$

From this, we can derive the total probability distribution at time *t* after initiation if all particles are inserted at a time distributed with uniform probability. Hence, we can find an equivalent form of Eq. E28 for the shuttling model. However, the form of this is not particularly illuminating and in the manuscript we present numerical solutions.

B.2.4 RNA Gradient Model

In the RNA gradient model, the proteins have finite lifetime, as in the SDD model. Therefore, we can use the result from[9] to find the average protein age in steady-state:

$$\tau(x) = \frac{1}{2\mu}\left[\left(1+\frac{x}{\lambda}\right)\frac{e^{-x/\lambda}}{e^{-x/\lambda} - \Lambda_s e^{-x/\lambda_s}} + \frac{2}{1-\Lambda_s^{-2}}\right] \tag{E30}$$

where $\Lambda_s = \lambda_s / \lambda$. In Figure 1C and Appendix Figure S1 we find good agreement between our simulations and Eq. E30 at long times. In Figure 1D we solve the model numerically (akin to described for the SDD model) to find the average protein lifetime at earlier times in a finite system.

B.2.5 *bcd* RNA Diffusion Model

In the RNA diffusion model, the *bcd* mRNA has finite lifetime but not the Bcd protein itself. Therefore, at a given position and time a protein have age given by $\tau = t - t_{prod}$. The time of production will be determined by the *bcd* RNA concentration. However, the *bcd* RNA in this case behaves, dynamically, the same as Bcd protein in the SDD model. Therefore, at long time ($\mu_{rna} t >> 1$) the average protein age is given by

$$< \tau(x) >_t = t - \frac{1}{2\mu_{rna}}\left(1+\frac{x}{\lambda_{rna}}\right) \tag{E31}$$

Eq. E31 fits the simulations for the *bcd* RNA model well, Figure 1D and Appendix Figure S1. In Figure 1D we calculate the average protein age numerically, accounting for finite boundary conditions. Eq. E31 shows that the general trend of old protein near the source is a general property of the *bcd* RNA diffusion model, regardless of particular parameters.

B.2.6 Theoretical predictions for mCherry/sfGFP ratio

From the above estimations of average protein age, we developed an understanding of the expected behavior of the timer ratio. To calculate the timer ratio for each model we extended the dynamic equations to include folding of both sfGFP and mCherry. Each protein can exist in six states: unfolded (C_u); sfGFP folded only (C_g); mCherry partial-folded only (C_{r*}); mCherry full folded (C_r); sfGFP folded and mCherry partial-folded (C_{gr*}); and both fluorophores folded (C_{gr}). We also confirmed our results using simulations of the fluorophore folding, Appendix Figure S1.

For the SDD model, Eq. E9 becomes

$$\begin{aligned}
\frac{\partial C_u}{\partial t} &= D\frac{\partial^2 C_u}{\partial x^2} - (\mu + \alpha + \beta_1)C_u + J\delta(x) \\
\frac{\partial C_g}{\partial t} &= D\frac{\partial^2 C_g}{\partial x^2} + \alpha C_u - (\mu + \beta_1)C_g \\
\frac{\partial C_{r*}}{\partial t} &= D\frac{\partial^2 C_{r*}}{\partial x^2} + \beta_1 C_u - (\mu + \beta_2 + \alpha)C_{r*} \\
\frac{\partial C_r}{\partial t} &= D\frac{\partial^2 C_r}{\partial x^2} + \beta_2 C_{r*} - (\mu + \alpha)C_r
\end{aligned} \tag{E32}$$

$$\frac{\partial C_{gr*}}{\partial t} = D\frac{\partial^2 C_{gr*}}{\partial x^2} + \beta_1 C_g + \alpha C_{r*} - (\mu + \beta_2) C_{gr*}$$

$$\frac{\partial C_{gr}}{\partial t} = D\frac{\partial^2 C_{gr}}{\partial x^2} + \beta_2 C_{gr*} + \alpha C_r - \mu C_{gr}$$

Therefore, the total Bcd concentration stills obeys Eq. E9 (the folding terms cancel).

It is straightforward to extend these equations to the other models and include FRET effects. For an extended discussion of modeling folding kinetics see[22]. The equations were solved using Matlab (pdepe) and the solutions compared with the Monte Carlo simulations, Appendix Figure S1. All model fitting to the mCherry/sfGFP ratio in Figure 3A,C and Appendix Figure S7 involves solving the full set of equations for different fluorophore states for each model (accounting for finite boundary conditions and interpretation prior to fully reaching steady-state).

B.3 Timer ratio without protein production

As has been previously proposed, Bcd protein production may cease in n.c. 14 (and potentially earlier[11]). To explore further, we considered the case

$$\frac{\partial C_{Bcd}}{\partial t} = D\frac{\partial^2 C_{Bcd}}{\partial x^2} - \mu_{14} C_{Bcd} \tag{E33}$$

where we account for potentially different rate of degradation in n.c. 14, with initial condition

$$C_{Bcd}(t=0,x) = \frac{J}{\sqrt{D\mu}} e^{-x/\sqrt{D/\mu}}$$

but no further production.

At $t=0$, the average protein concentration was given by Eq. E26. First, ignoring diffusion, the concentration at a particular position decreases as $e^{-\mu_{14}t}$ with time (μ_{14} being the degradation rate in n.c. 14). Since protein degradation is independent of position and other proteins, the probability of a particular protein degrading is independent of its state (e.g. which fluorophores have folded). However, the average age of each protein at time t after cessation of production goes as $<\tau(x)>_t \frac{1}{2\mu}\left(1+\frac{x}{\lambda}\right)+t$, since, although the total protein number has decreased significantly, the age of each surviving protein must be older. Therefore, the ratio of mCherry to sfGFP signal approaches one, independently of the value of μ_{14} (as $<\tau(x)>$ is independent of μ_{14}). Of course, μ_{14} plays an important role in the experimental validation of this result – large μ_{14} results in very low concentrations, and subsequently much noisier experimental measurements of the mCherry/sfGFP ratio. Including diffusion introduces potential dependence on μ_{14}. However, the timescale for diffusion over a significant fraction of the embryo is larger than the expected timescale for degradation. e.g. the diffusion time over scale of 50μm (10% embryo length) is ~ 20 minutes, whereas the degradation time scale is around 15

minutes. Therefore, locally over a few nuclei the role of diffusion is important but at the scale of the embryo the degradation dominates in cycle 14 (assuming degradation is increased). We see that since degradation dominates the timescales at the embryo level in cycle 14 (assuming no production) then the mCherry/sfGFP ratio is largely independent of the degradation rate.

B.4 Simulation details

One dimensional Monte Carlo simulations were written in Matlab and run on a server PC with 128Gb memory. Simulations took between one hour and one day to complete, depending on the diffusion constant, particle number and the desired simulation time.

The simulations were performed in the region 0<x<L, where L=500μm. Discretizing in space, we defined Δx as the distance between points, so we typically had $N_x = L/\Delta x + 1$ spatial positions (+1 accounts for x=0). Simulations were run for a total time T, so we had $N_T = (T/\delta t) + 1$ time points (+1 accounts for t=0).

In the simulations there were three probabilities: $P(J) = J\delta t$ the probability that in time t->t+δt a particle was inserted at *x=0*, $P(\mu) = \mu\delta t$, the probability that in time t->t+δt a particle decays and $P(D) = \frac{D\delta t}{(\Delta x)^2}$, the probability that in time t->t+δt a particle moves. Generally, P(D) was the largest probability and hence we defined $\delta t = \frac{1}{2}\frac{(\Delta x)^2}{D}$ so that each time step each particle moves either left or right (except if at x=0 or x=L).

At each time step a uniformly distributed random number in (0,1) was drawn to determine the number of newly injected particles based on *P(J)* (assuming Poisson statistics for insertion). Next, for each particle a uniformly distributed random number in (0,1) was drawn. If it was less than P(μ) the particle decayed. Finally, a uniformly distributed random number in (0,1) was drawn for each particle: if it was less than 0.5 the particle moved left; if it was greater than 0.5 the particle moved right. The behavior of each individual particle was saved to allow investigation of the particle lifetime (it is much faster to run the simulations based on total particle number but then the local particle age information is lost). Simulation results were checked against analytical predictions and there was always excellent fit, giving confidence in the results.

We also performed simulations where individual particles could fold green, fold red or both. As above, we assigned a probability for each folding event based on the experimentally measured folding times. As shown in Figure 1 and Appendix Figure S1 there was good agreement between simulations and analytical approaches.

We also numerically integrated the above equations with finite boundary conditions using Matlab pdepe. We ensured that the numerical solutions (both simulations and numerical integration) and analytical solutions were in good agreement at long times.

B.5 Simulations of the gradient dynamics before equilibrium

In order to study the development of the Bcd gradient during stages 4 and 5 (Figure 6), we implemented a version of the SDD model that includes the maturation of the fluorophores in the timer- such as we did before- and additionally allows for the possibility of changing the production and degradation rates parameters during the simulations.

This model was implemented in R, using the ReacTran package (https://CRAN.R-project.org/package=ReacTran)[23], which allows building models that describe reaction and advective-diffusive transport. We implemented a 1-D diffusive model. The simulations were run on a MacBook Pro with a 2.5 GHz Intel Core i7 processor and 16 Gb memory. Each simulation took approximately 60 min. The geometry of the model is a one-dimensional grid composed of 500 finite Δx of 1 μm, with zero-gradient boundary conditions. The production of tFT-Bcd was restricted to the first five grid cells to mimic an anteriorly restricted source, while all the other parameters were invariant in space. The simulations were run for 1200- 4000 time points (sec). The initial abundance of all species was 0. The reactions and species included are depicted in the following scheme, Theory Figure 2:

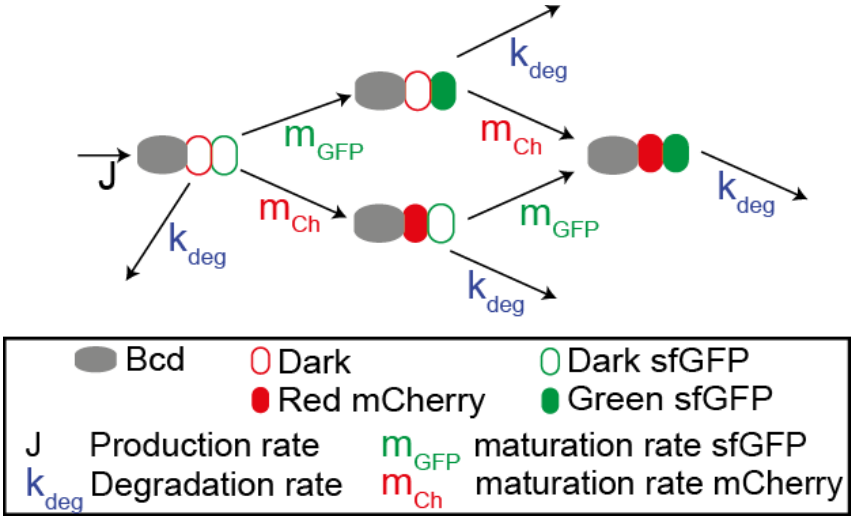

Theory Figure 2: Schematic of reaction scheme used in modeling SDD through blastoderm development

To study the effects on the reporter of the possible escape of free GFP from degradation, we included an additional reaction that gives free GFP as a product of degradation, "$k_{inc\text{-}deg}$" and a diffusion coefficient for free GFP "D_{gfp}" (Appendix Figure S4C). The incomplete degradation of the timer was implemented as a fraction of the full degradation, thus "$k_{inc\text{-}deg}$" is a probability. The Diffusion of free GFP was consider as 3 times the diffusion of the full construct because it has aproximately 1/3 of its size. For the analysis that required variation in the production and degradation rates, we considered them variables with a constant value except when changed at a certain time in the simulations (typically t=600 sec). The change was modeled as an exponential decay, with decay rate "dg" for the degradation rate and "dp" for the production rate (Appendix Figure S11).

C. Appendix Tables

Appendix Table 1. List of parameter values used in Figure 6B

Parameter	Initial phase	1	2	3	4	5	6
j_{ini} (Mol/sec)	4000	**0**	4000	4000	**8000**	**2000**	**1000**
k_{ini} (sec^{-1})	0.4	0.4	**0.8**	**0**	0.4	**0.2**	**0.8**
$K_{deg\text{-}inc}$	0	0	0	0	0	0	0
**D** (μm^2/sec)	2	2	2	2	2	2	2
D_{gfp} (μm^2/sec)	D*3	D*3	D*3	D*3	D*3	D*3	D*3
m_{gfp} (sec^{-1})	1.5	1.5	1.5	1.5	1.5	1.5	1.5
m_{ch} (sec^{-1})	0.6	0.6	0.6	0.6	0.6	0.6	0.6
**dg** (sec^{-1})	0	0	0	0	0	0	0
**dp** (sec^{-1})	0	0	0	0	0	0	0

The initial phase corresponds to times 0-600 and the following to times 600-1200.

Appendix Table 2. List of parameter values used in Appendix Figure S4C

Parameter	Magenta line	Blue line	Light blue line	Orange line
j_{ini} (Mol/sec)	500	4000	4000	4000
k_{ini} (sec^{-1})	0.2	1.2	1.2	1.2
$K_{inc\text{-}deg}$	**0**	**0.0001**	**0.001**	**0.01**
**D** (μm^2/sec)	2	2	2	2
D_{gfp} (μm^2/sec)	D*3	D*3	D*3	D*3
m_{gfp} (sec^{-1})	0.2	1.5	1.5	1.5
m_{ch} (sec^{-1})	0.01	m_{gfp}/2.5	m_{gfp}/2.5	m_{gfp}/2.5
**dg** (sec^{-1})	0	0	0	0
**dp** (sec^{-1})	0	0	0	0

Appendix Table 3. List of parameter values used in Appendix Figure S11

Parameter	Value
j_{ini} (Mol/sec)	4000
k_{ini} (sec^{-1})	1.2
$K_{deg\text{-}inc}$	0
**D** (μm^2/sec)	2

**D_{gfp}** (μm^2/sec)	D*3
**m_{gfp}** (sec^{-1})	1.5
**m_{ch}** (sec^{-1})	0.6
**dg** (sec^{-1})	**10**
**dp** (sec^{-1})	10

D. Determination of the maturation kinetics of the fluorescent proteins

Fluorescent proteins need to maturate before they become fluorescent, a process that takes an average time that is different for each fluorophore. Using of the tandem timer fluorescent proteins requires knowledge of the maturation kinetics of the fluorophores in the tandem pair. The maturation kinetics of the fluorophores have been previously estimated in yeast[22]. However, the cellular context plays an important role in determining folding times – in particular temperature, pH and oxygen availability[24,25].   – so we measured the folding rates in the *Drosophila* embryo.

The approach we chose is to produce *in vitro* RNA of the tandem timer constructs, inject it on early embryos and then follow the fluorescence in time. Previous estimations of fluorophores maturation in live cells were based on a timed induced expression and successive inhibition of protein translation. However, there are no good expression systems for the early drosophila embryo - in which the activation of the genome has not happened yet or is going on at the moment-, so we decided to inject RNA directly. . We use the RNA of the full timer to maintain similarity to the age-measurements. Further, fitting is improved since the only difference between the red and green signals are the maturation kinetics.

D.1 RNA injection of embryos

RNA copies of the full tFT construct (mCherry-linker-sfGFP) were produced by *in vitro* transcription and injected to wt y/w embryos. For obtaining the embryos, young flies were caged two days before the experiment. The morning of the experiment three 45 min pre-laids were performed followed by a 30 min lay. The plates were allowed to develop 30 min at RT and then the embryos were dechorionated 40 sec in 50% sodium hypochlorite solution. Following, the embryos were taken to the 18ºC injection room and mounted on a glass coverslip pretreated with embryo glue (glue from adhesive tape extracted with heptane). For the injection process only, the coverslip with the embryos was mounted over a glass slide for additional support. They were dehydrated for 8 min in a salts chamber and they were covered with a drop of halocarbon oil 700/27 (1:2; Sigma-Aldrich).

The injection needles were pulled from borosilicate glass capillaries (1.2-mm outer diameter and 0.94-mm inner diameter; Harvard Apparatus), using a P-97 Flamming/brown puller (Sutter Instrument).

The embryos were mounted such that three fitted in the microscope field of view. The first embryo of each trio was left un-injected as a control, and the other two were injected in the posterior pole with RNA 100 ng/μl. The injections were performed using a microinjector (model 5242; Eppendorf). The injection time was 0.5 sec in every case, and the injection pressure was calibrated for each needle and liquid to produce a drop of the same approximate volume. All the embryos were injected within 1-2 min.

D.2 Imaging and image analysis

Sequential imaging of ten positions (each containing 1 control and 2 injected embryos) was started as soon as possible, approximately 10 min after finishing the injections. The embryos were immersed in PBS and imaged at RT using a two-photon microscope (LSM 780 NLO; Carl Zeiss) with a 20x objective. The time resolution of the time courses was 1 min and their duration was between 30 and 160 min. One central z-slice of each embryo was acquired, with the confocal pinhole set to 5 a.u..

Image quantification of mCherry and sfGFP fluorescence intensities over time were done with ImageJ.

D.3 Modeling of maturation rates and data fitting

Estimation of maturation kinetics was performed using a model adapted from Khmelinskii *et al.*[22]. We modeled sfGFP maturation as a one-step process and mCherry maturation as a two-step process, in accordance with chemical reactions leading to chromophore formation[22,26-28]. For fmCherry, one-step and two-step models were tested (equivalent to sfGFP and mCherry models). To constrain the total amount of mCherry to be equal to that of sfGFP (as is the case biochemically), and improve the power and robustness of the fit, joint production and degradation kinetics of the fusion protein were considered, Appendix Figure S6A. We assume that the non-fluorescent precursor A is produced with a constant production rate *p*. sfGFP can mature in a single reaction with rate *m*, while mCherry requires two consecutive reactions with rates m_1 and m_2, respectively. Degradation of all fusion isoforms occurs with the same constant rate *k*. This system can be described with the following set of rate equations:

$$
\begin{aligned}
\frac{dA}{dt} &= p - m_1A - mA - kA \\
\frac{dB}{dt} &= m_1A - m_2B - mB - kB \\
\frac{dC}{dt} &= m_2B - mC - kC \\
\frac{dD}{dt} &= mA - m_1D - kD \\
\frac{dE}{dt} &= mB + m_1D - m_2E - kE \\
\frac{dF}{dt} &= mC + m_2E - kF
\end{aligned}
\qquad \text{(E34)}
$$

To map the amounts of fluorescent protein species C, D, E, and F to the observed fluorescence intensities and account for autofluorescence, we introduce scaling factors and offsets for each channel:

$$\text{mCherry}_{\text{observed}} = C_{\text{mCherry}} \cdot (C + F) + \text{offset}_{\text{mCherry}} \qquad \text{(E35)}$$

(E36)

$$sfGFP_{observed} = C_{sfGFP} \cdot \left(D + E + F \cdot (1 - E_{FRET})\right) + offset_{sfGFP}$$

where E_{FRET} is the FRET efficiency and was taken to be 0.173[22]. Since the production rate and scaling factors are structurally non-identifiable[22], we set the production rate to 1 and estimated only the scaling factors. Furthermore, to account for delay between RNA injection and protein production, a lag time τ was introduced, such that $p = \begin{cases} 0, & t < \tau \\ 1, & t \geq \tau \end{cases}$. The degradation rate can be decomposed into effective degradation k_{deg} and dilution k_{cycle}, such that $k = k_{deg} + k_{cycle}$. Since the timer construct does not have any degradation signals, we assume $k_{deg} = 0$. Furthermore, since *D. melanogaster* embryos at this stage undergo cell division without increasing in volume, $k_{cycle} = 0$. We therefore set $k = 0$.

We assume the initial amount of all protein species to be 0 and estimate a single value of each maturation rate m, m_1, and m_2 globally for the entire data set, while scaling factors, offsets, and time delay parameters are estimated separately for each embryo. Parameter estimation was performed in MATLAB (MathWorks) using the D2D framework[29,30].

With this approach we we able to fit all the parameters, scaling factors and offsets and they are all identifiable, as determined by likelihood profile analysis (Appendix Figure S6D-F). For mCherry, we estimated m_1 = 0.017 min^{-1} (uncertainty range between 0.014 min^{-1} and 0.022 min^{-1}), and m_2 = 0.076 min^{-1} (0.053 min^{-1}, 0.104 min^{-1}) (Appendix Figure S6G). The half time of the maturation is equal to log(2)/m, so for mCherry we get T_1= 40 ± 9 min, and T_2= 9 ± 4 min. For sfGFP we estimated m = 0.025 min^{-1} ,with an uncertainty range between 0.023 and 0.027 min^{-1}, which is equivalent of a maturation half-time of T= 27 ± 2 min (Appendix Figure S6B-D, G). For fmCherry we estimated m_1 = m_2= 0.112 min^{-1} (uncertainty range between 0.094 min^{-1} and 0.141 min^{-1}), T_1= T_2= 6 ± 2 min(Appendix Figure S6C and F-G). We note that the measured sfGFP and mCherry characteristic folding time are larger than previously estimated[22,31]. It is not surprising that the fluorophore maturation rate in the *Drosophila* embryo is slower, considering that oxygen availability is likely lower as a result of low permeability and high demand.

E. Additional references

1. Driever, W. & Nüsslein-Volhard, C. A gradient of bicoid protein in Drosophila embryos. *Cell* **54,** 83–93 (1988).
2. Gregor, T., Wieschaus, E. F., McGregor, A. P., Bialek, W. & Tank, D. W. Stability and Nuclear Dynamics of the Bicoid Morphogen Gradient. *Cell* **130,** 141–152 (2007).
3. Bergmann, S. *et al.* Pre-Steady-State Decoding of the Bicoid Morphogen Gradient. *PLoS Biol* **5,** e46 (2007).
4. de Lachapelle, A. M. & Bergmann, S. Precision and scaling in morphogen gradient read-out. *Mol Sys Biol* **6,** 351 (2010).
5. Coppey, M., Berezhkovskii, A. M., Kim, Y., Boettiger, A. N. & Shvartsman, S. Y. Modeling the bicoid gradient: Diffusion and reversible nuclear trapping of a stable protein. *Dev. Biol.* **312,** 623–630 (2007).
6. Dalessi, S., Neves, A. & Bergmann, S. Modeling morphogen gradient formation from arbitrary realistically shaped sources. *J. Theor. Biol.* **294,** 130–138 (2012).
7. Grimm, O., Coppey, M. & Wieschaus, E. Modelling the Bicoid gradient. *Development* **137,** 2253–2264 (2010).
8. Spirov, A. *et al.* Formation of the bicoid morphogen gradient: an mRNA gradient dictates the protein gradient. *Development* **136,** 605–614 (2009).
9. Berezhkovskii, A. M., Sample, C. & Shvartsman, S. Y. How long does it take to establish a morphogen gradient? *Biophys. J.* **99,** L59–61 (2010).
10. Dilão, R. & Muraro, D. mRNA diffusion explains protein gradients in Drosophila early development. *J. Theor. Biol.* **264,** 847–853 (2010).
11. Little, S. C., Tkačik, G., Kneeland, T. B., Wieschaus, E. F. & Gregor, T. The Formation of the Bicoid Morphogen Gradient Requires Protein Movement from Anteriorly Localized mRNA. *PLoS Biol* **9,** e1000596 (2011).
12. Hecht, I., Rappel, W.-J. & Levine, H. Determining the scale of the Bicoid morphogen gradient. *Proc. Natl. Acad. Sci. U.S.A.* **106,** 1710–1715 (2009).
13. Gordon, P. V., Sample, C., Berezhkovskii, A. M., Muratov, C. B. & Shvartsman, S. Y. Local kinetics of morphogen gradients. *Proc. Natl. Acad. Sci. U.S.A.* **108,** 6157–6162 (2011).
14. Gregor, T., Tank, D. W., Wieschaus, E. F. & Bialek, W. Probing the Limits to Positional Information. *Cell* **130,** 153–164 (2007).
15. Krzic, U., Gunther, S., Saunders, T. E., Streichan, S. J. & Hufnagel, L. Multiview light-sheet microscope for rapid in toto imaging. *Nat Meth* **9,** 730–733 (2012).
16. Schmid, B. *et al.* High-speed panoramic light-sheet microscopy reveals global endodermal cell dynamics. *Nat Comm* **4,** 1–10 (2013).
17. He, F. *et al.* Distance measurements via the morphogen gradient of Bicoid in Drosophila embryos. *BMC Dev Biol* **10,** 80–14 (2010).
18. Rauzi, M. *et al.* Embryo-scale tissue mechanics during Drosophila gastrulation movements. *Nat Comm* **6,** 8677 (2015).
19. Sommer, C., Straehle, C. & Kothe, U. ilastik: Interactive learning and segmentation toolkit. *IEEE International Symposium on Biomedical Imaging* (2011).
20. Houchmandzadeh, B., Wieschaus, E. & Leibler, S. Establishment of

developmental precision and proportions in the early Drosophila embryo. *Nature* **415,** 798–802 (2002).
21. Deng, J., Wang, W., Lu, L. J. & Ma, J. A two-dimensional simulation model of the bicoid gradient in Drosophila. *PLoS ONE* **5,** e10275 (2010).
22. Khmelinskii, A. *et al.* Tandem fluorescent protein timers for in vivo analysis of protein dynamics. *Nat Biotechnol* **30,** 708–714 (2012).
23. Soetaert, K. & Meysman, F. Reactive transport in aquatic ecosystems: Rapid model prototyping in the open source software R. *Environmental Modelling and Software* **32,** 49–60 (2012).
24. Miyawaki, A., Shcherbakova, D. M. & Verkhusha, V. V. Red fluorescent proteins: chromophore formation and cellular applications. *Current Opinion in Structural Biology* **22,** 679–688 (2012).
25. Macdonald, P. J., Chen, Y. & Mueller, J. D. Chromophore maturation and fluorescence fluctuation spectroscopy of fluorescent proteins in a cell-free expression system. *Analytical Biochemistry* **421,** 291–298 (2012).
26. Shu, X., Shaner, N. C., Yarbrough, C. A., Tsien, R. Y. & Remington, S. J. Novel chromophores and buried charges control color in mFruits. *Biochemistry* **45,** 9639–9647 (2006).
27. Subach, O. M. *et al.* Structural Characterization of Acylimine- Containing Blue and Red Chromophores in mTagBFP and TagRFP Fluorescent Proteins. *Chem. Biol.* **17,** 333–341 (2010).
28. Hebisch, E., Knebel, J., Landsberg, J., Frey, E. & Leisner, M. High Variation of Fluorescence Protein Maturation Times in Closely Related Escherichia coli Strains. *PLoS ONE* **8,** e75991–9 (2013).
29. Raue, A. *et al.* Lessons Learned from Quantitative Dynamical Modeling in Systems Biology. *PLoS ONE* **8,** e74335–17 (2013).
30. Raue, A. *et al.* Data2Dynamics: a modeling environment tailored to parameter estimation in dynamical systems. *Bioinformatics* **31,** 3558–3560 (2015).
31. Shaner, N. C., Steinbach, P. A. & Tsien, R. Y. A guide to choosing fluorescent proteins. *Nat Meth* **2,** 905–909 (2005).

F. Appendix figures and legends

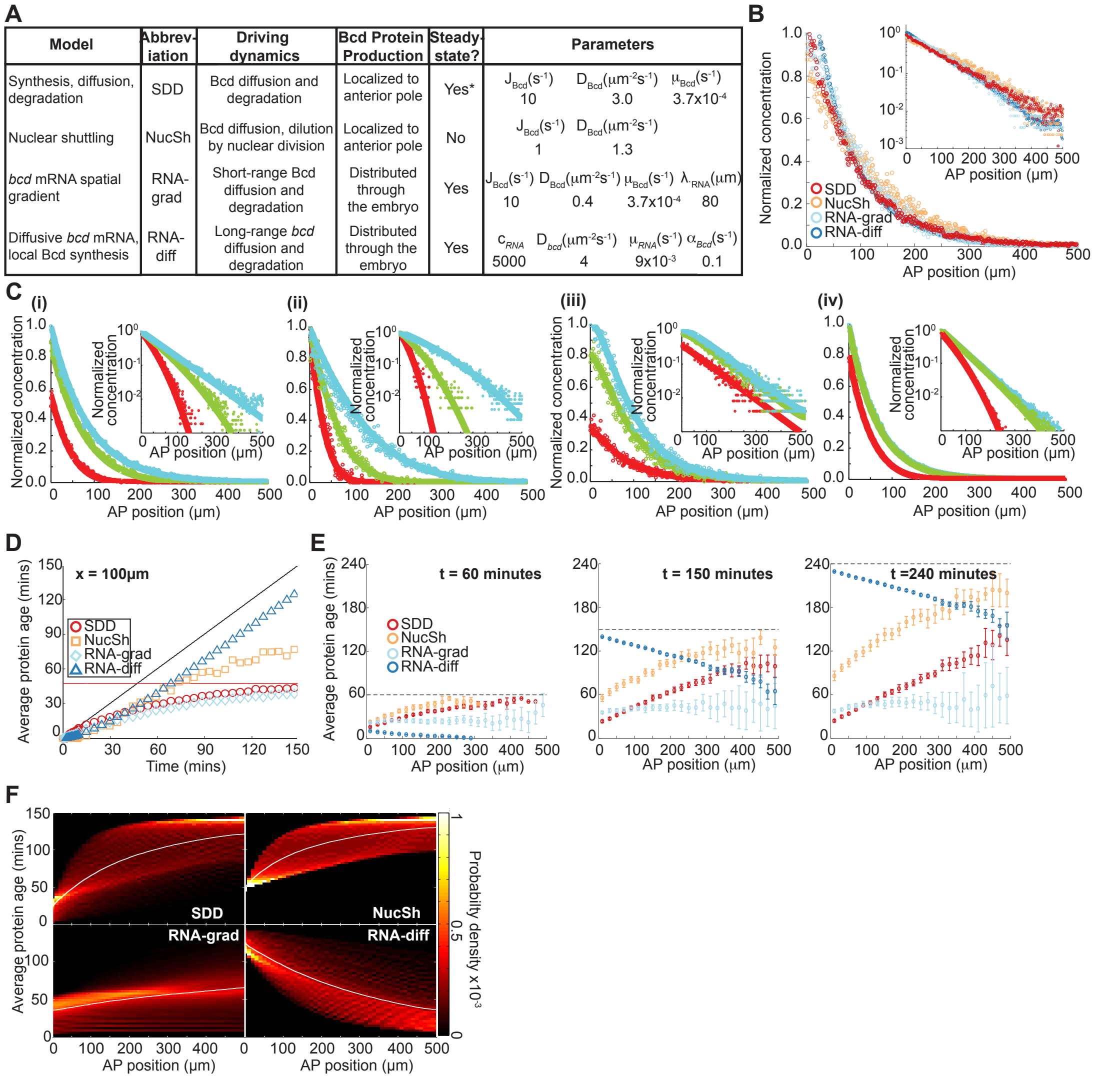

Appendix Figure S1 Protein age simulations for four alternative BCD gradient formation models .

A Models of Bcd gradient formation considered in the manuscript along with mean parameters used in the simulations. J_{Bcd}: Bcd protein production rate, D_{Bcd}: Bcd protein diffusion coefficient, μ_{Bcd}: Bcd protein degradation rate, λ_{RNA}: length scale of the exponentially distributed mRNA gradient , c_{RNA}: amount of bcd mRNA molecules, μ_{RNA}: bcd mRNA degradation rate, α: Bcd protein production. More details in Appendix section B1.

B Normalized concentration profiles from Monte Carlo simulations of the four models considered after 150 mins. Inset shows the profiles on a logarithmic scale.

C. Monte carlo simulations (points) along with theoretical fits (see Theory) for the four different models. i) SDD model, ii) Nuclear shuttling, iii) RNA gradient, iv) RNA diffusion. Red corresponds to 60 mins, green to 150 mins , and magenta to 240 mins.

D Average protein age as function of time at x=100μm from simulations as described above.

E Average protein age at different system times (60, 150, 240 minutes) from Monte

Carlo simulations. Error bars correspond to s.d. from 10 independent runs.
F Probability heat map of protein age in different models considered. Parameter range as described in Figure 1E, except profiles not constrained to match experimental profile (i.e. here, all solutions are shown). Unless otherwise stated, all parameters as **A.**

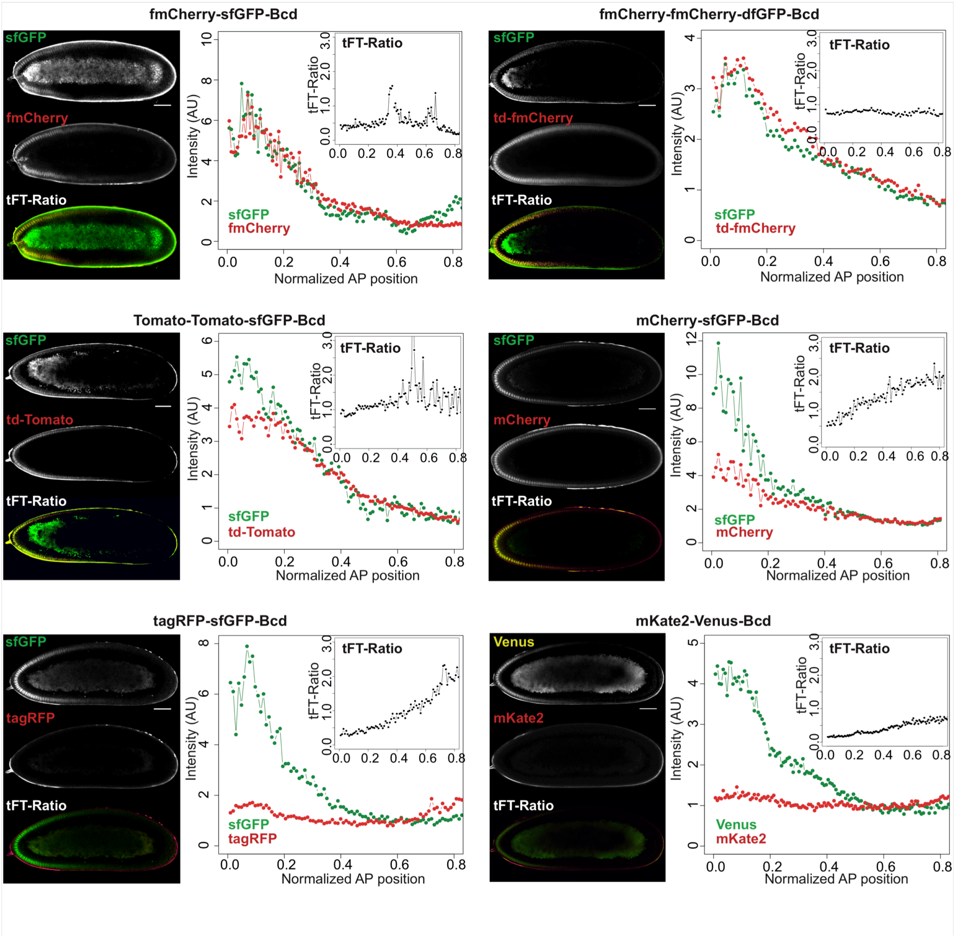

Appendix Figure S2 Comparison of six different Bcd- tandem timer fluorescent reporter lines.
Confocal images of a mid-sagittal plane of the six different tFT fluorophore lines constructed. All lines were generated by landing site insertion on the chromosome II with an identical construct with Bcd tagged with a different tandem reporter under *bcd* regulatory sequences as outlined in Methods. For the intensity profiles, each profile has been normalized to its own values at 60-70% of the embryos length (note that the y axis is different due to brightness differences amongst the fluorophores). The x axis is normalized to the total embryo length. A representative embryo of each line is shown. **Left:** Images for the green and red channels and weighted ratio. **Right:** Quantification of the fluorescence intensity for each channel. **Inset:** mCherry/sfGFP ratio. Of the fluorescent tandem timers tested, only mCherry-sfGFP (and in a lesser degree the fmCherry-sfGFP) reporters is useful as it has (i) signal in both channels and (ii) a significant difference in their profiles. Scale bars are 50 μm long.

A

	TM3/BcdE1 (%)	BcdE1/BcdE1 (%)
Observed Females	72 +- 7	28 +- 7
Observed Males	74 +- 3	26 +- 3
Observed- All	73 +- 3	27 +- 3
Expected if rescues	66.6	33.3
Expected if doesn't rescue	100	0

B

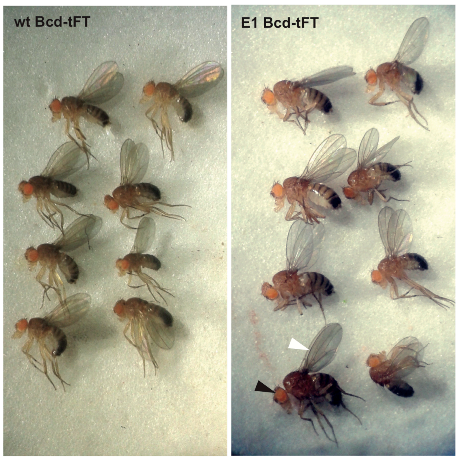

Appendix Figure S3 The tFT-Bcd is functional.

A The tFT-Bcd construct rescues a lethal null Bcd mutation. Percentage of phenotypes in the F1 of a rescue of Bcd^{E1} (bloomington stock 3630) experiment. Brother-sister crosses of flies with the genotype P{tFT-Bcd};+; th' st' kni^{ri-1} bcd^{6} rn^{roe-1} p^{p}/TM3,sb^{1} were performed. 250 flies were examined.

B Images of flies with the tandem reporter, comparing flies in wt and in Bcd^{E1} backgrounds. The mutant flies show mild abnormalities in the torax and an irregular eye color that could be due to the linked mutation rn roe1 (black arrow). Some flies show the shortage of the vein L2, characteristic of homocigosis in the Bcd^{E1} associated mutation Kni ri-1 (white arrow).

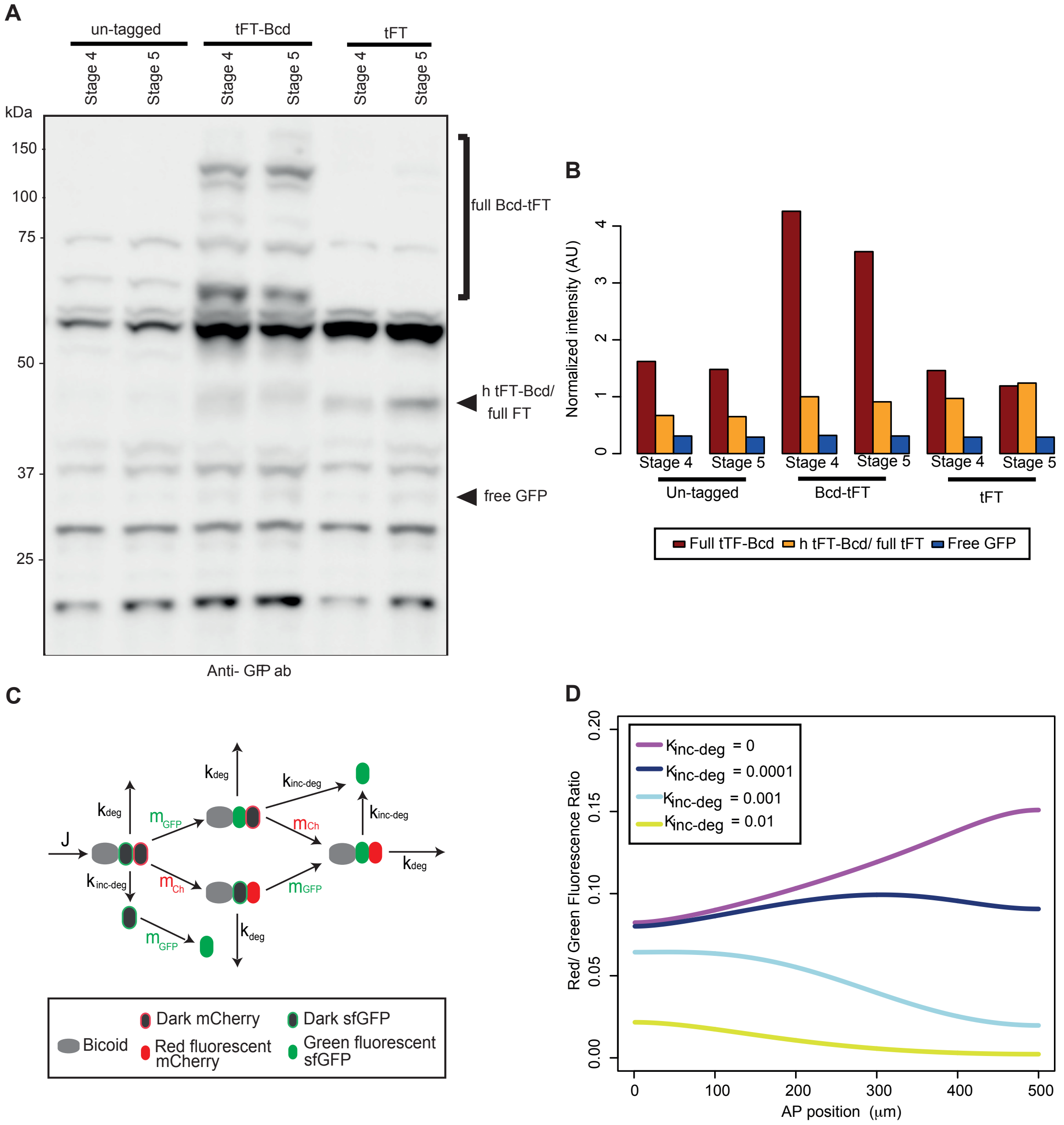

Appendix Figure S4 There is no significant escape of free GFP after tFT-Bcd degradation.

A Immunoblot of embryos expressing constructs indicated above, probed with an antibody against GFP. Stage 4 embryos, where the Bcd gradient is building, and stage 5, when Bcd is degraded, were collected. Multiple bands are observed for each construct, due to the previously reported distribution of bands of both Bcd and the tFT. The expected sizes for the full construct and its partial degradation products are indicated on the right (note that there are no visible bands of free GFP).

B Quantification of the gel in A.

C Cartoon of the model used to explore the possible impact of having partial degradation of the tFT – with escape of free GFP - on the measured gradients. The model is identical to the one used in Figure 6, but including an additional reaction of degradation of the tFT-Bcd (no matter its maturation status) that gives free GFP as a product ($K_{inc-deg}$).

D Theoretical expectation in the case of survival of free GFP after tFT-Bcd degradation. Simulations were performed with the model depicted in **C**. Red/green fluorescent ratio is shown as a function of position along the anterior-posterior axis. Setting $K_{inc\text{-}deg}$=0 (no incomplete degradation of the timer construct, equivalent to the model in Figure 6) predicts a monotonically increasing curve, consistent with experimental data (Figs 2-6). However, even low values of incomplete degradation produce curves that are inconsistent with the experimental observations.

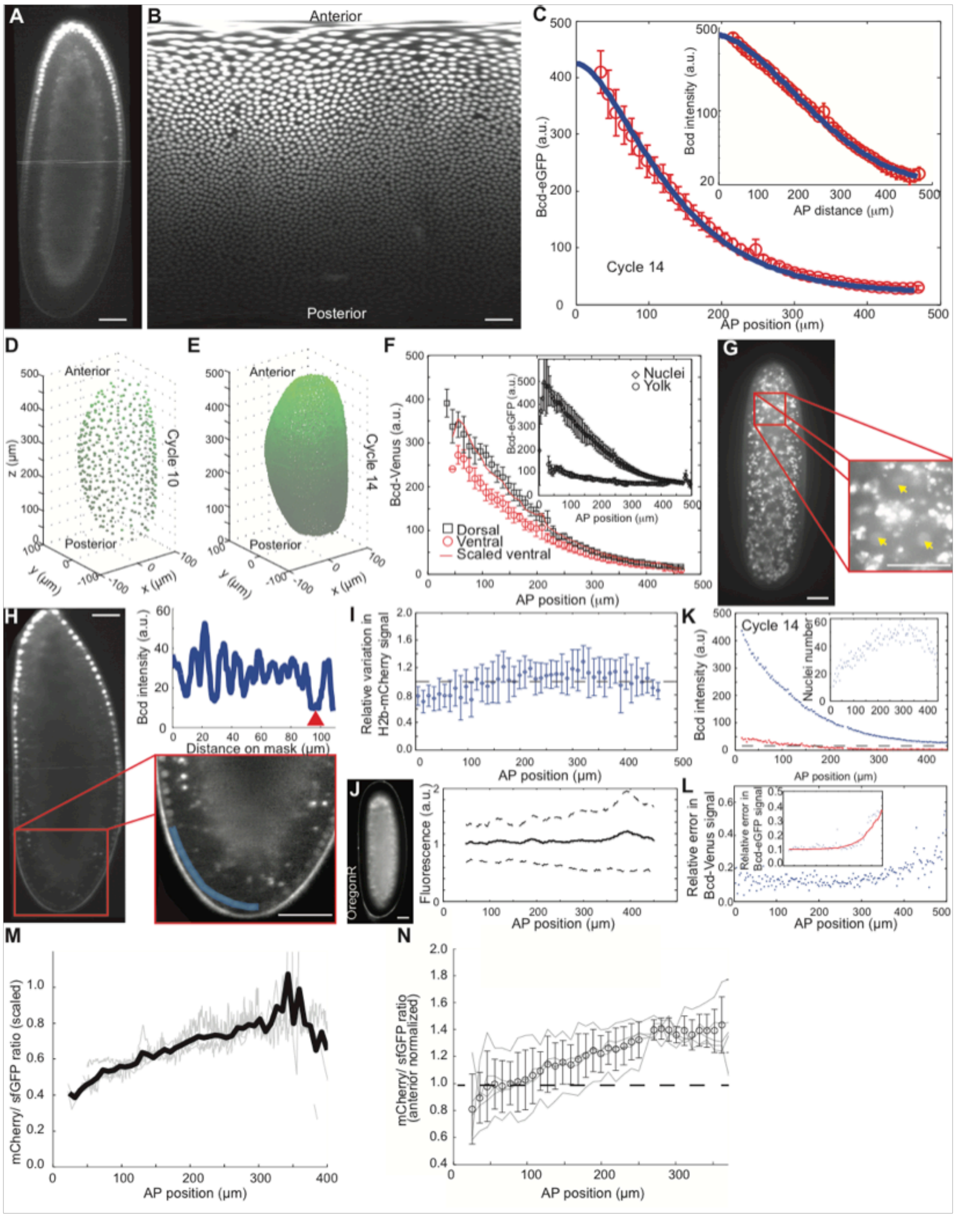

Appendix Figure S5 Light-sheet fluorescence microscopy using a confocal slit can record quantitative data of the Bcd gradient *in toto* .

Images were acquired with the MuVi-SPIM microscope. Scale bars are 50 μm long.

A Single plane at the center of an embryo expressing Bcd-Venus in n.c. 14. The anterior pole of the embryo is on the top.

B 2D projection “carpet” of the embryo’s cortex. Corresponds to the full 3D imaging of the embryo shown in **A**.

C Quantification of Bcd-eGFP gradient (n=4) in early n.c. 14. The blue line is the fit is to SDD model accounting for fluorophore folding time (~45 minutes for eGFP).

D- E 3D reconstruction of Bcd concentration in nuclei after unrolling and segmentation. **D**, **E** corresponds to n.c. 10 and 14 respectively.

F Bcd-Venus profile in n.c. 14 differs between dorsal and ventral sides of embryo. However, such differences do not significantly alter our main conclusions. Inset shows relative intensity of Bcd in nuclei relative to yolk.

G n.c. 8 embryo expressing Bcd-Venus. Zoomed region highlights Bcd-Venus positive nuclei (yellow arrows).

H Early n.c. 14 embryo expressing Bcd-Venus. Zoomed region highlights Bcd-Venus expression in nuclei in the posterior pole. Bcd-Venus intensity profile along the mask shown in the inset. Red arrow denotes most posterior position along mask.

I Variation in nuclear histone-mCherry signal as function of AP position in early n.c. 14. Error bars are standard deviation within each bin.

J Mean cortical fluorescence intensity of an un-tagged OregonR embryo along cortex taken with same microscope settings as Figs. 2-3. Dashed lines represent ±1s.d.

K Bcd-eGFP profile in n.c. 14 (blue dots) with error (red dots). Data from individual nuclei binned into 5 mm bins. Inset shows number of nuclei against distance from anterior pole.

L Relative error (standard deviation divided by mean intensity within each 5 μm bin) of Bcd-Venus (main panel) and Bcd-eGFP (inset). The red line is a fit to the theoretical prediction if noise is predominantly due to Poisson fluctuations.

M Imaging of the tFT-Bcd is reproducible between sessions. mCherry-sfGFP-Bcd ratio as function of position including data sets taken on confocal and LSFM at different times and conditions. Each profile (gray lines) is scaled by a single multiplicative factor (after background subtraction) to account for, as an example, variation in laser excitation intensity or microscope alignment.

N mCherry-sfGFP-Bcd ratio, normalized by the ratio in the anterior pole (set to 0.9 in region 50 μm from anterior).

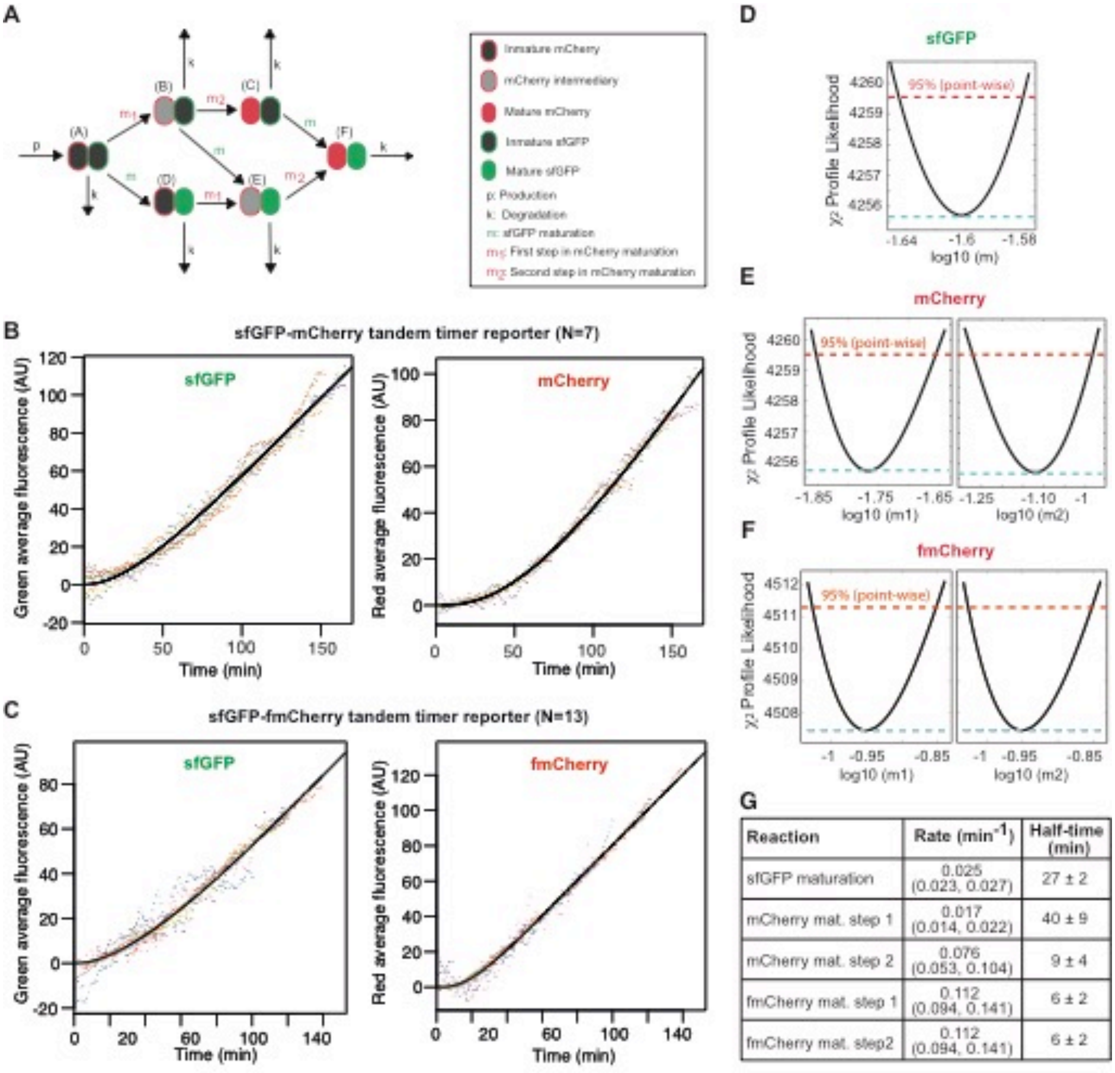

G

Reaction	Rate (min^{-1})	Half-time (min)
sfGFP maturation	0.025 (0.023, 0.027)	27 ± 2
mCherry mat. step 1	0.017 (0.014, 0.022)	40 ± 9
mCherry mat. step 2	0.076 (0.053, 0.104)	9 ± 4
fmCherry mat. step 1	0.112 (0.094, 0.141)	6 ± 2
fmCherry mat. step2	0.112 (0.094, 0.141)	6 ± 2

Appendix Figure S6 Experimental estimation of the fluorophores maturation times.

Experimental determination of the maturation rates of sfGFP, mCherry and fmCherry in the tFT constructs in the *Drosophila* early embryo. Stage 4 embryos were injected with tFT mRNA, and confocal imaging of the green and red fluorescence was started 10 minutes later.

A Scheme of the model used for the data fitting.

B Experimental data (colored dots) and best fit (black line) for the mCherry-sfGFP tFT reporter.

C Experimental data (colored dots) and best fit (black line) for the sfGFP-fmCherry tFT.

D-F Likelihood profile analysis of the fitted maturation rates for sfGFP, mCherry and fmCherry respectively. The light blue dtted line marks the lowest cost, and the red one the highest acceptable cost. All parameters were identifiable.

G The fitted maturation rates and uncertainty ranges for each reaction. The maturation half-time is equal to log(2)/ maturation rate.

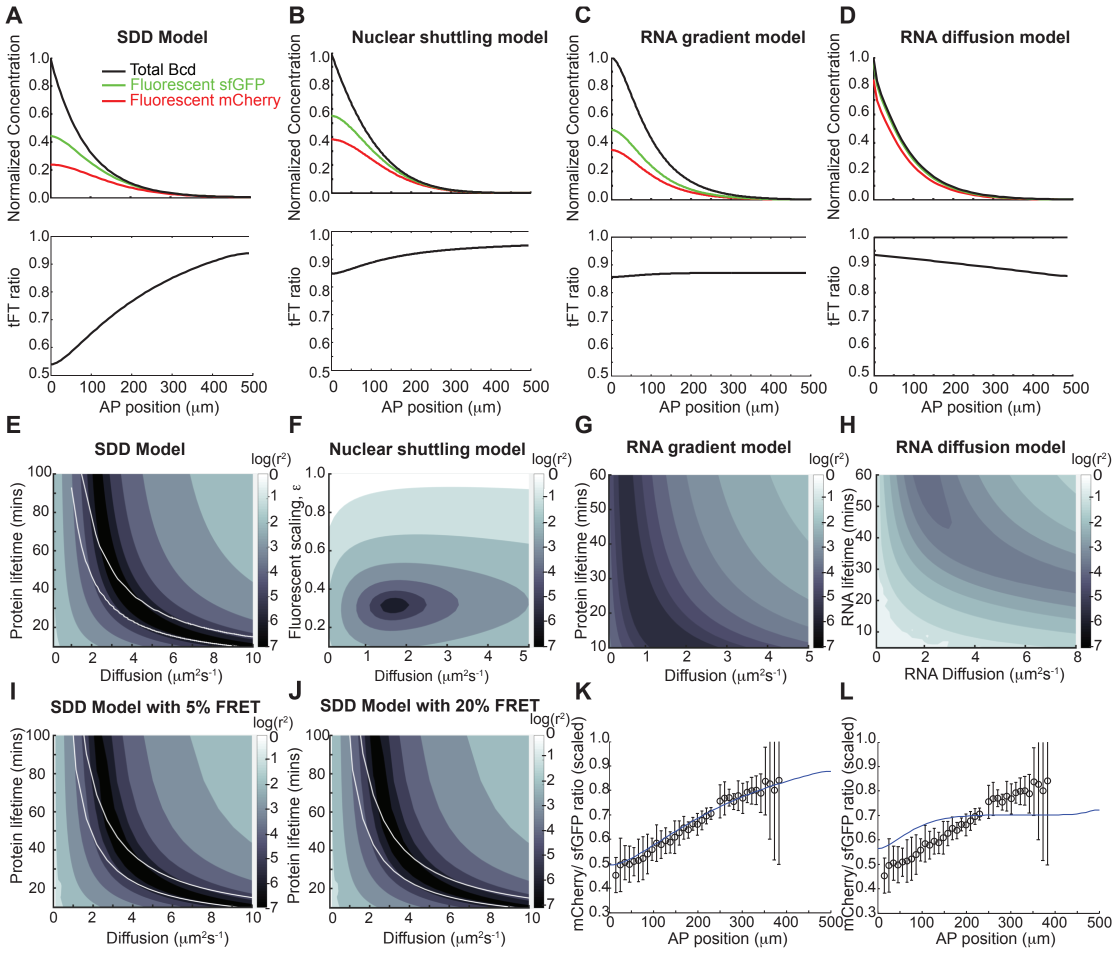

Appendix Figure S7 Fitting the tFT-Bcd ratio using different models for gradient formation.

A- D Top: Predicted concentration profiles (black = total Bcd, green = sfGFP, red = mCherry) and tFT-Bcd ratio (**bottom**) for all four models considered using representative parameters.

E- H Fit quality (see Methods) of different models to measured tFT-Bcd ratio for different parameter ranges. White line represents parameters that result in a profile that is exponential-like with decay length ~85mm. In **F**, for the nuclear shuttling model, there is only one parameter (diffusion). In this case, the y-axis corresponds to fits at different times of development. In **H**, diffusion and degradation refer to the mRNA, not the Bcd protein.

I- J As Figure 3b, but accounting for 5% (**J**) and 20% (**K**) FRET effect respectively. Fitting of the tFT-Bcd ratio using the SDD model with 5% FRET effect.

K- L Fitting of the tFT-Bcd ratio using the RNA gradient model with an RNA gradient of length 40mm (**N**) and 100mm (**O**).

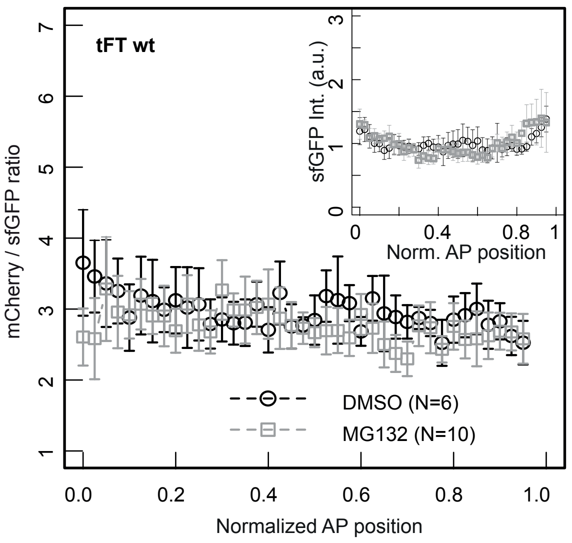

Appendix Figure S8 The inhibitor MG132 does not affect the tFT life-time.

The proteasome inhibitor MG132 has no impact on the average age of tandem constructs without the Bcd protein (gray, open squares) or in the total fluorescence (**inset**). All embryos are y/w and were injected while in n.c. 14 and imaged 60 min later.

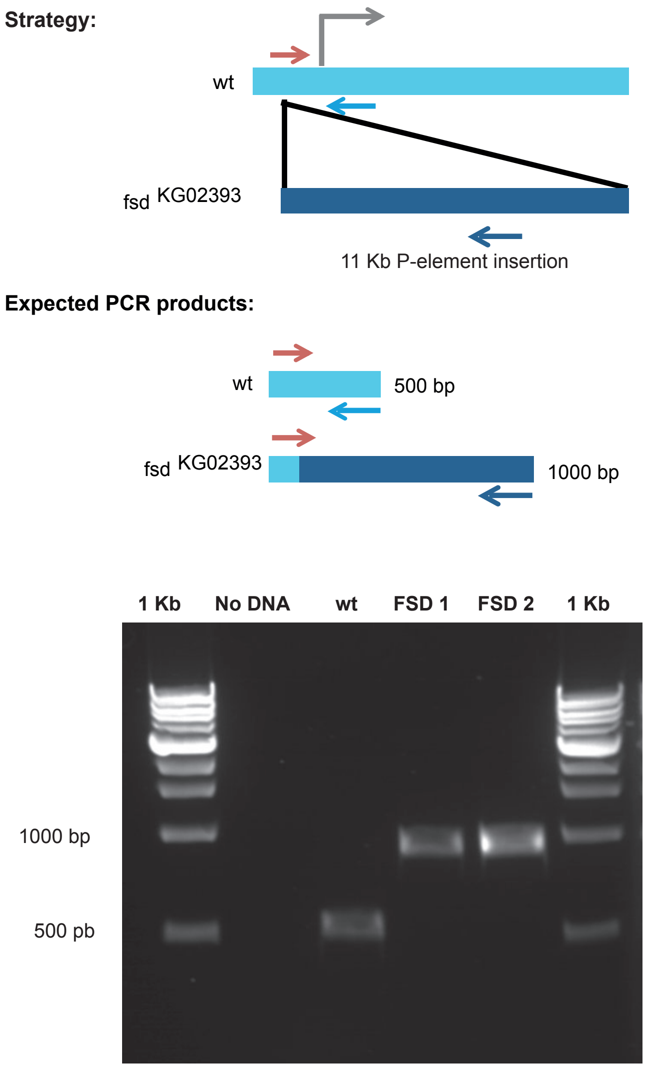

Appendix Figure S9 Corroboration of the *fsd* mutation.
Top: Strategy for line check by PCR Alternative reverse primers are used for the wt or mutant versions, giving different amplified fragments. **Bottom**: Agarose gel of the PCR. The mutant lines show the higher weight bands as expected.

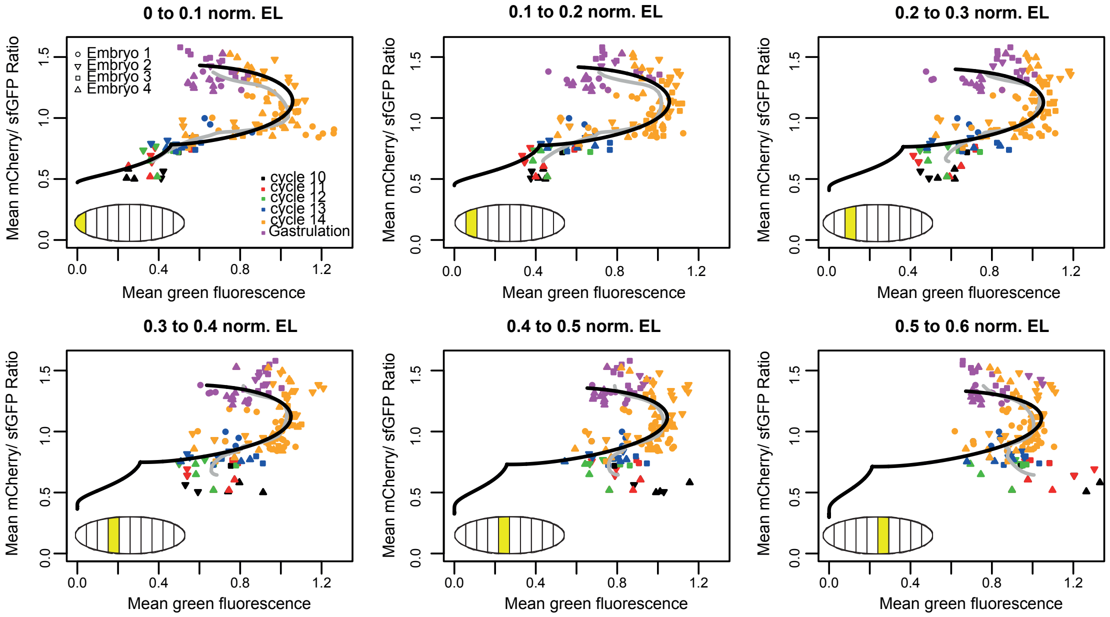

Appendix Figure S10 The selection of the embryo region quantified does not change qualitatively the dynamic trajectories.

Plots of Bcd protein age vs protein abundance through the early development were performed as in Figure 6 C-D, using the same data. However, different sections of the embryo across the AP axis were quantified (described in the title and sketched in the lower left corner, EL: Embryo Length). Comparison of simulations (black lines) and experimental data (colored symbols), both quantified in the same section, shows a general agreement. The quality of the data and the agreement diminishes towards the posterior pole due to the lower signal (and higher signal/noise) given by Bcd gradient.

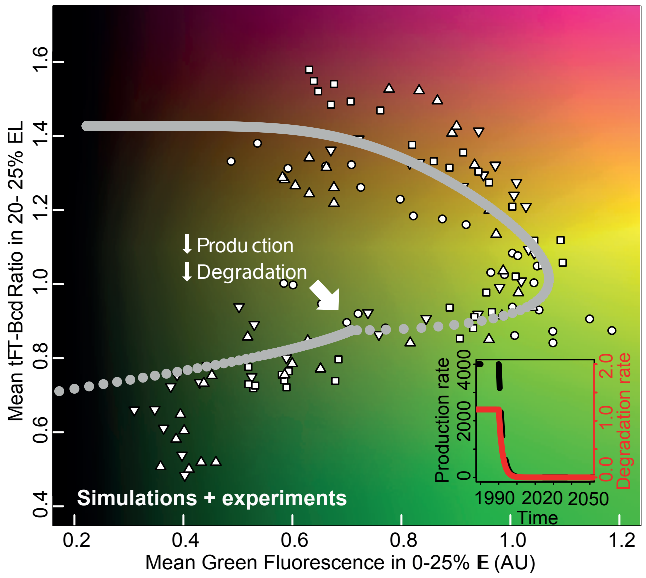

Appendix Figure S11 The SDD model structure with varying production and degradation rates can reproduce the experimental trajectories in the levels-age space.

Simulation of the SDD model mimicking the full early development (white continuous line), plotted over the experimental data points from Figure 6**D**. The simulations were performed to reproduce the experimental trajectories from Figure 6**D**. Two gradual changes were introduced following build-up of the gradient: an decrease in production and a decrease in degradation (**inset**).

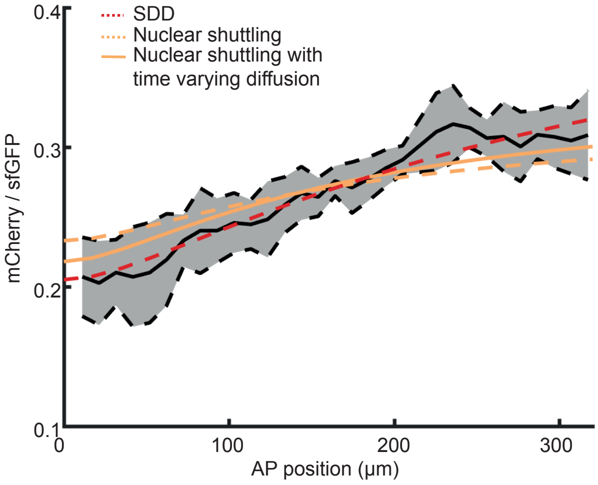

Appendix Figure S12 Fitting of nuclear shuttling model with time dependent diffusion.

Fitting of the nuclear shuttling model (as described in text) to the mCherry/sfGFP ratio (solid orange). Dashed lines correspond to SDD (red) and nuclear shuttling (orange) models with fixed parame

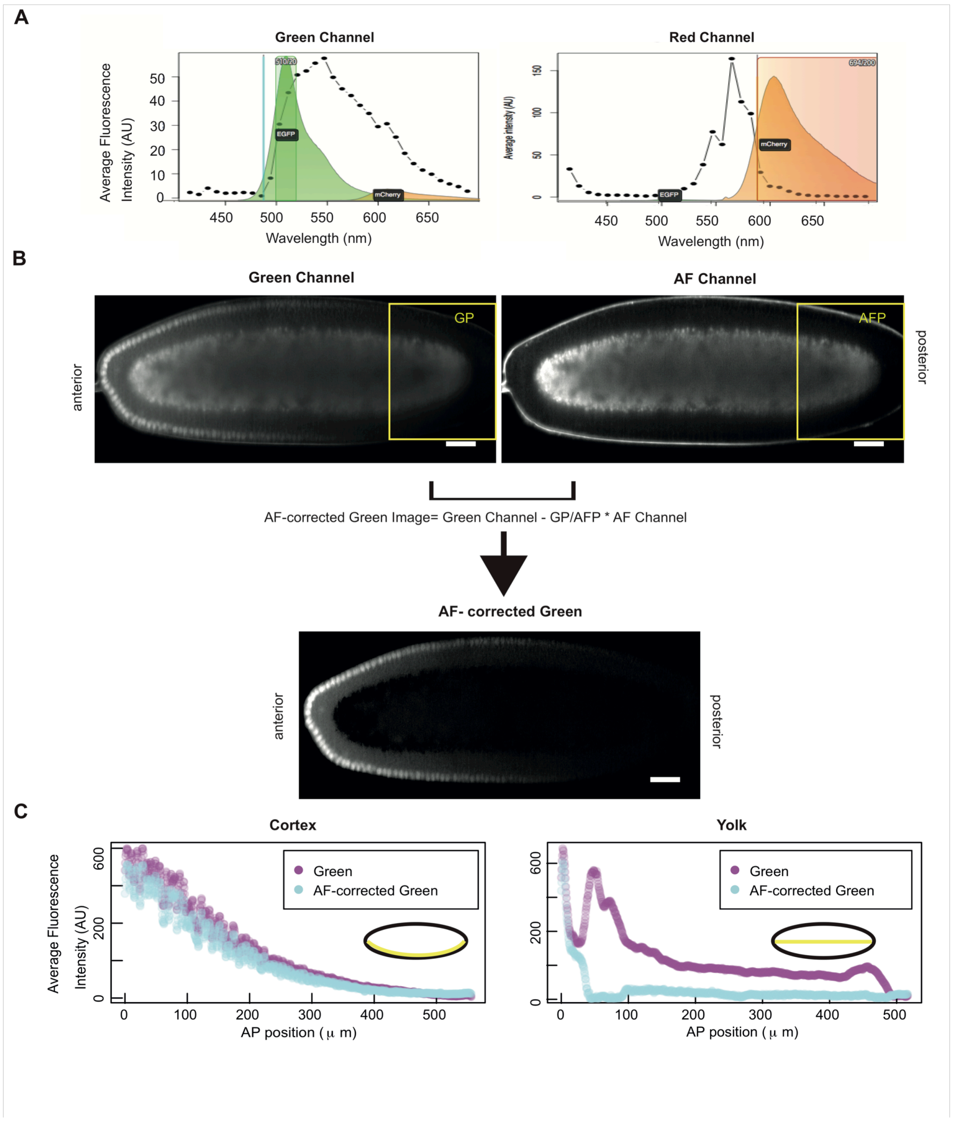

Appendix Figure S13 Background subtraction using linear unmixing

A Overlay of the sfGFP and mCherry emission spectra *in vitro* (http://www.bdbiosciences.com/us/s/spectrumviewer) and the early embryo autofluorescence spectrum (black lines and dots). The autofluorescence profile was acquired from 5 wt embryos of the y/w background (not expressing any fluorescent proteins) by performing a lambda scan on a confocal microscope. The autofluorescence and the GFP spectra overlap extensively.
B Basic workflow for linear unmixing. For each embryo and time point where the tFT was assessed throw-out this work, the red and green channels were recorded along with the "AF" channel (laser 488 nm, filter 594 nm). This channel was intended specifically for autofluorescence correction. The AF channel was chosen for showing a high correlation with the signal observed on the green channel in 5 wt embryos imaged with the MuViSPIM, but no GFP signal (as seen in A left). The AF channel image was then used to estimate the (spatially dependent) background signal in the green channel of fluorescent proteins-expressing embryos. This correction image was weighted and subtracted to the green channel image.

C Example of the impact of the linear unmixing on the fluorescence intensity quantification of a single embryo. The fluorescence profiles along the AP axis are shown. In the cortex area (left), where the mCherry-sfGFP-Bcd localizes, the correction has little effect. However on the yolk, where signal is expected to be due only to autofluorescence, the linear unmixing converts a gradient-looking profile into a no fluorescence flat line.
