# Supplementary figures and images for: Bicoid gradient formation mechanism and dynamics revealed by protein lifetime analysis
Source: Mol Syst Biol. 2018 Sep 4;14(9):e8355. doi: 10.15252/msb.20188355 (PMC6121778; doi:10.15252/msb.20188355)

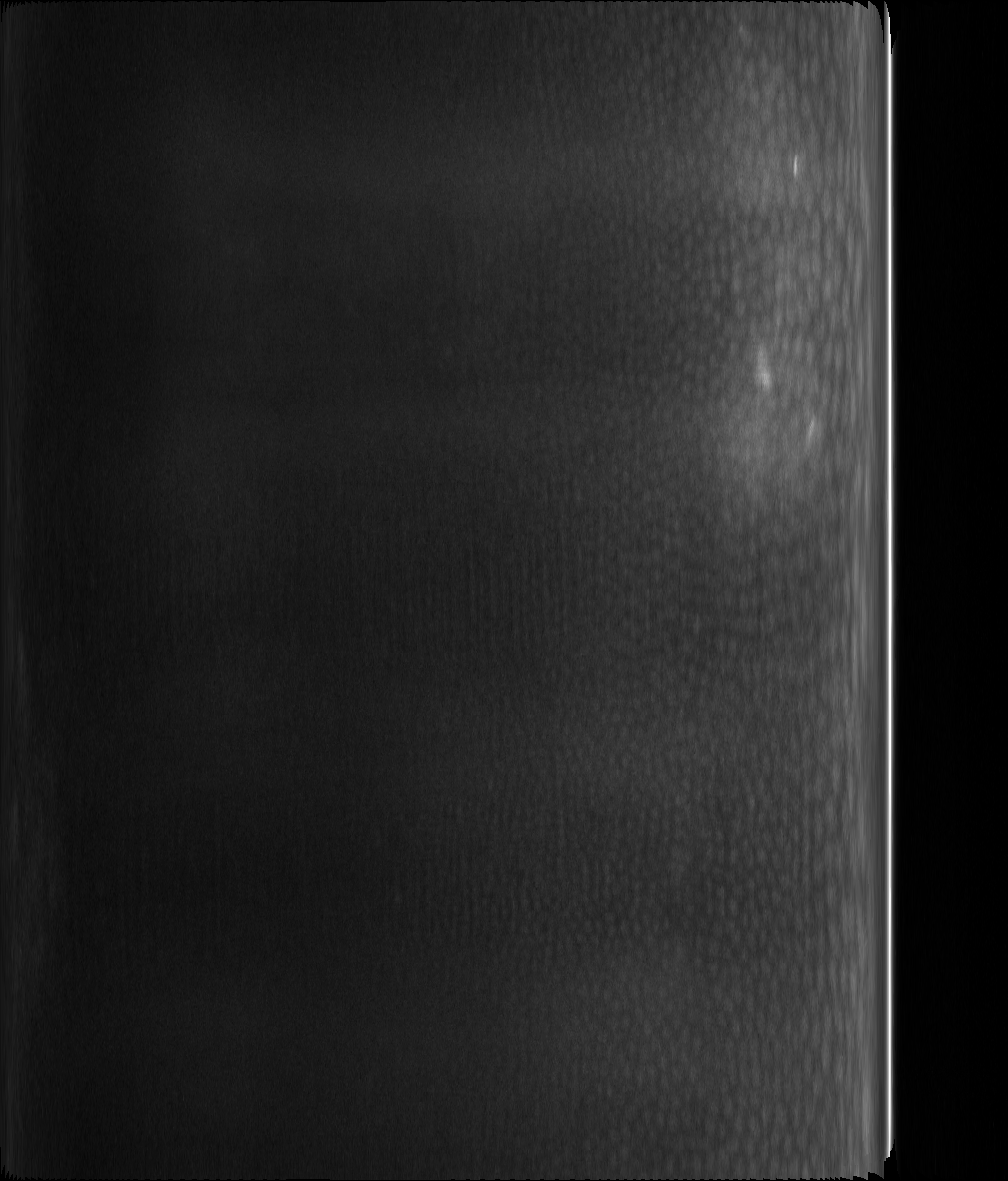

Supplement: Supplementary file 4 — Source Data for Figure 2 [file MSB-14-e8355-s003.zip › figure2-data/carpets_fmCherry-sfGFP-Bcd/emb10_g_fused_carpet.tif]

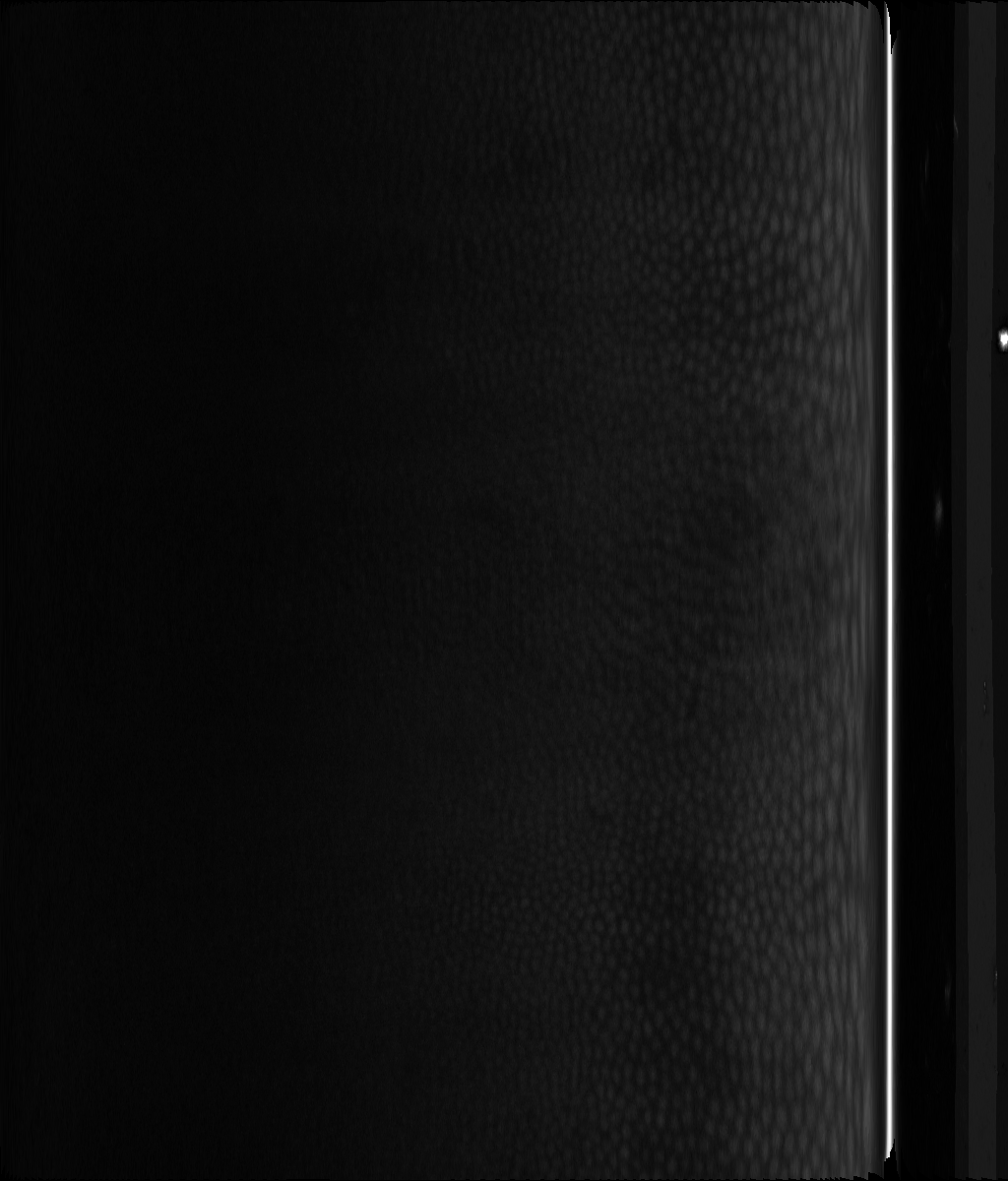

Supplement: Supplementary file 4 — Source Data for Figure 2 [file MSB-14-e8355-s003.zip › figure2-data/carpets_fmCherry-sfGFP-Bcd/emb10_r_fused_carpet.tif]

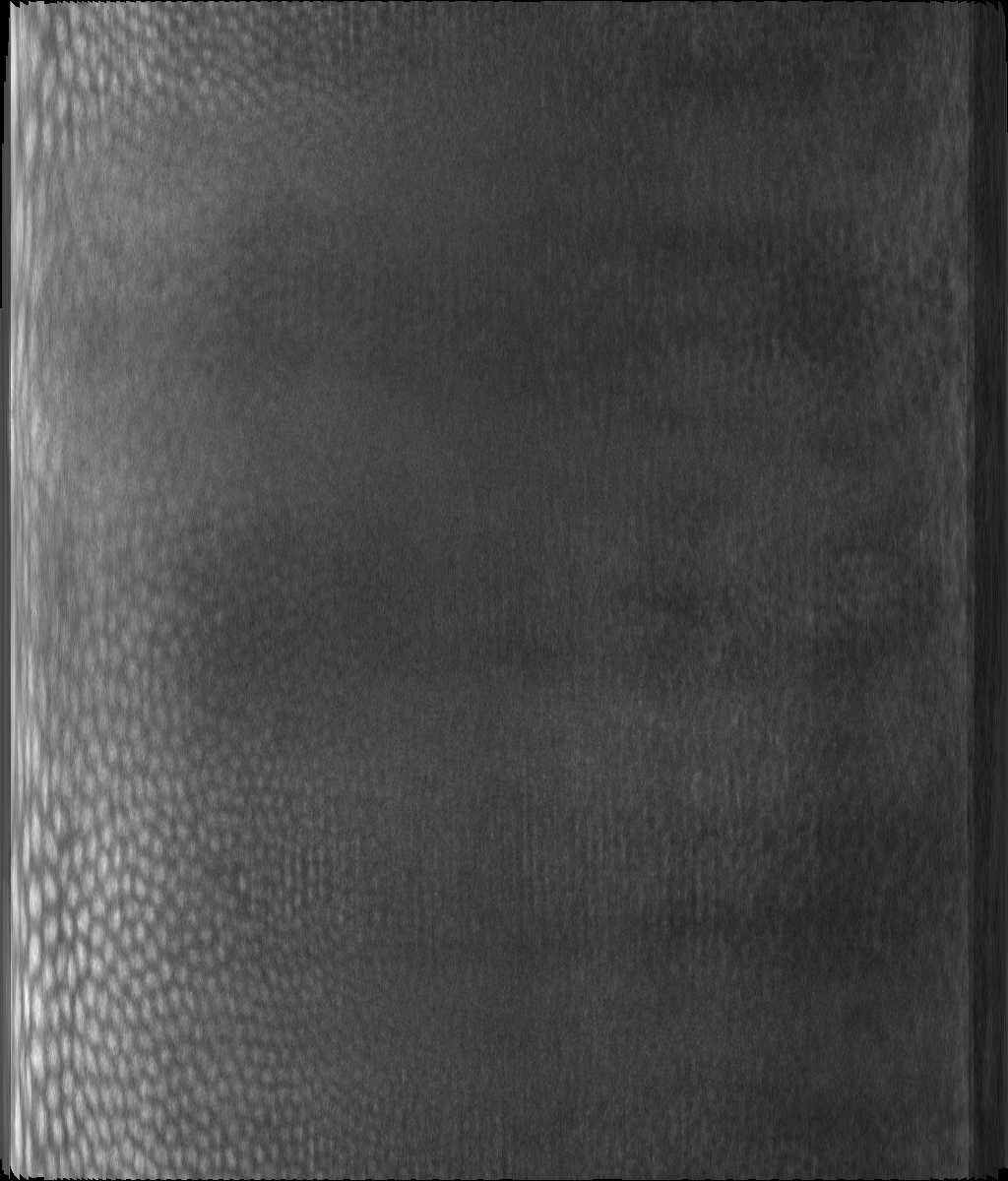

Supplement: Supplementary file 4 — Source Data for Figure 2 [file MSB-14-e8355-s003.zip › figure2-data/carpets_fmCherry-sfGFP-Bcd/emb1_g_fused_carpet.tif]

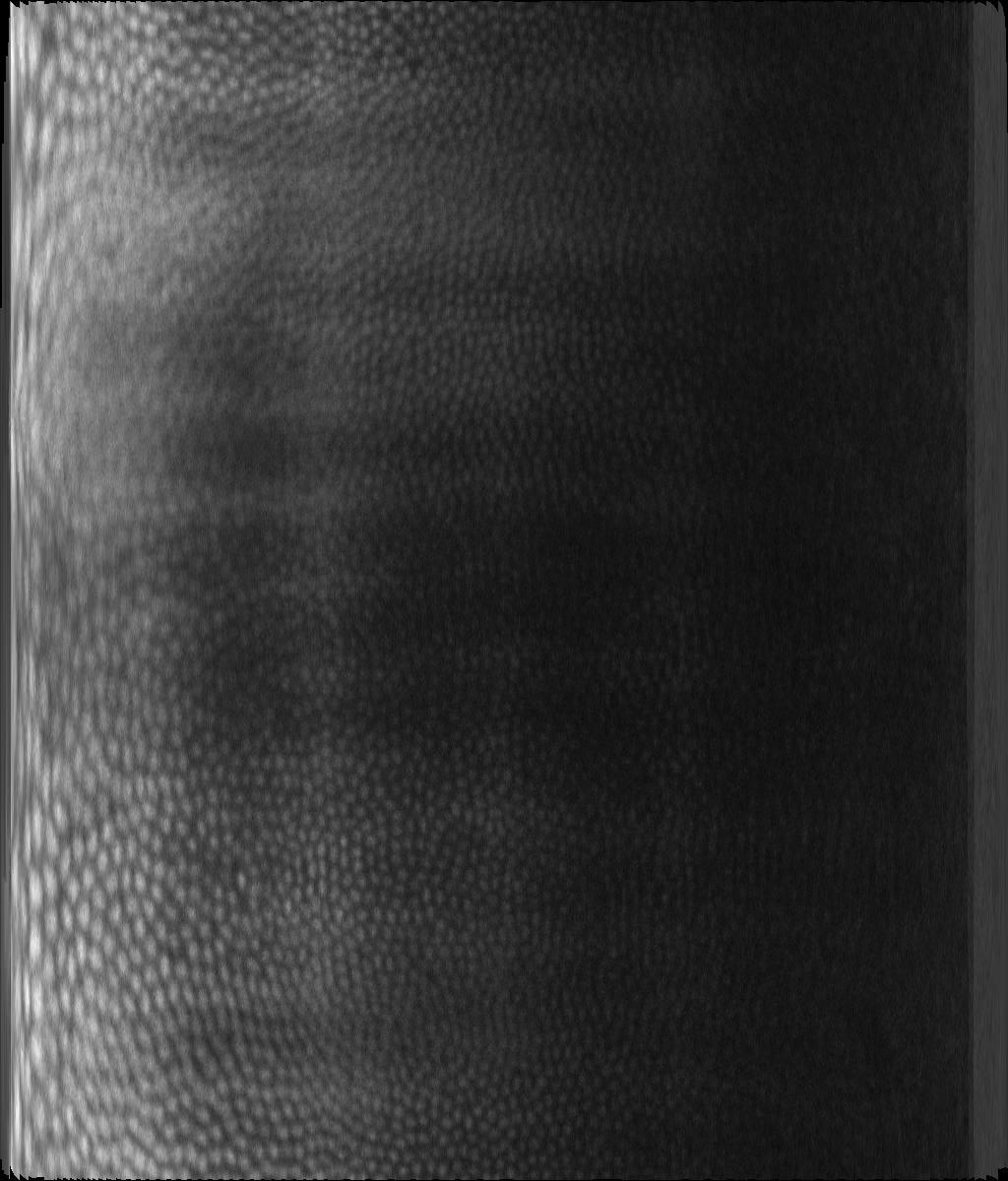

Supplement: Supplementary file 4 — Source Data for Figure 2 [file MSB-14-e8355-s003.zip › figure2-data/carpets_fmCherry-sfGFP-Bcd/emb1_r_fused_carpet.tif]

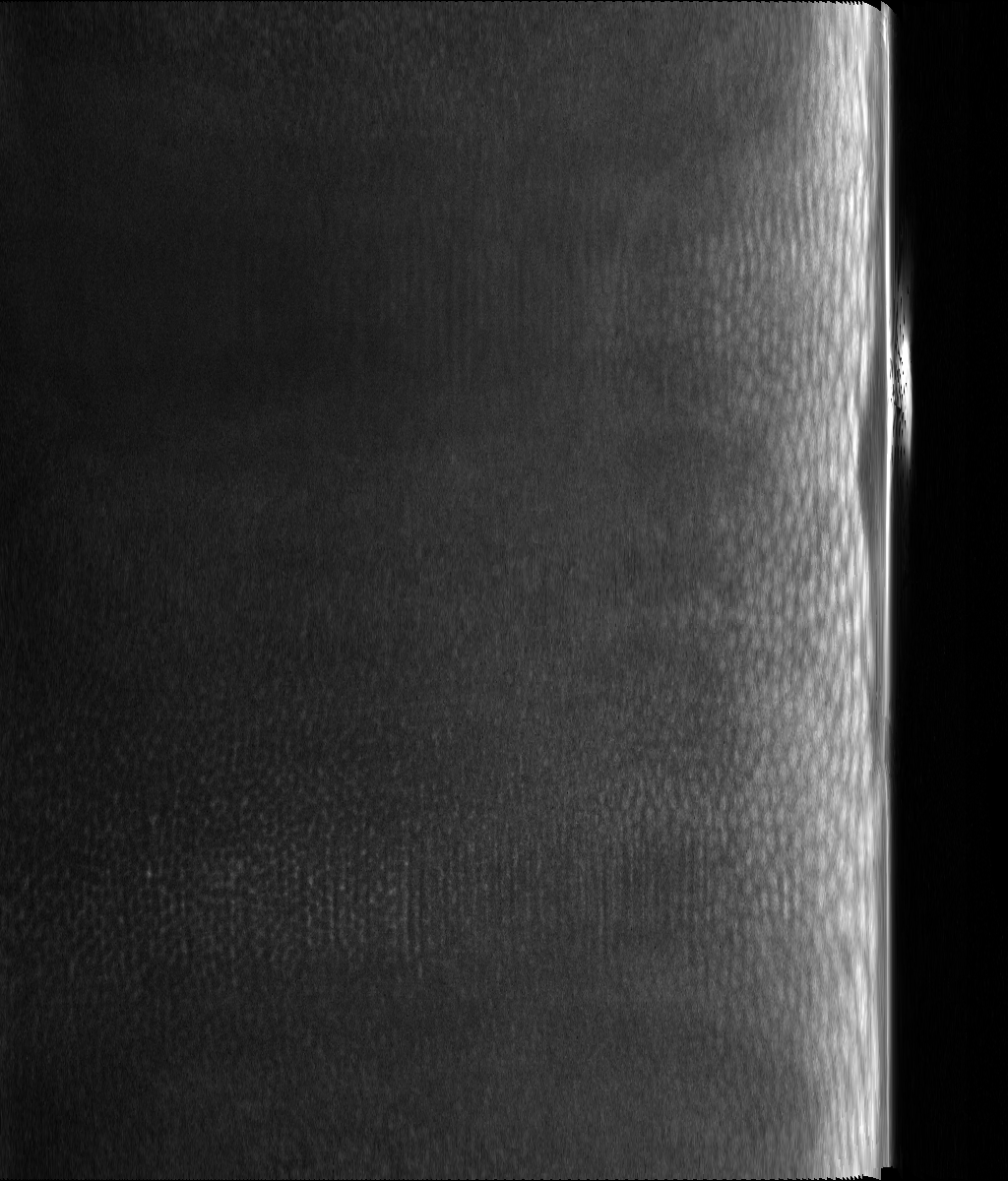

Supplement: Supplementary file 4 — Source Data for Figure 2 [file MSB-14-e8355-s003.zip › figure2-data/carpets_fmCherry-sfGFP-Bcd/emb2_g_rot0_carpet.tif]

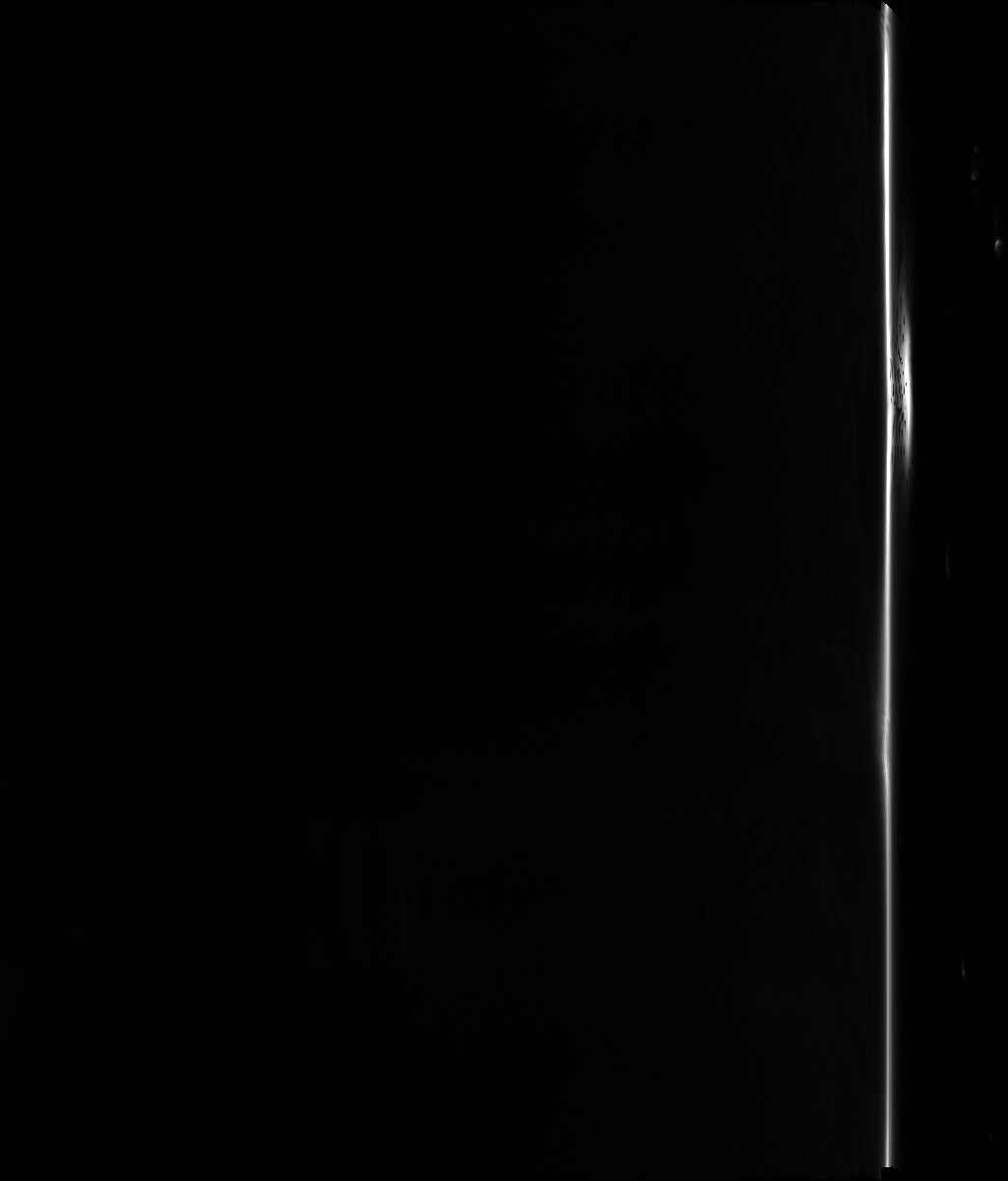

Supplement: Supplementary file 4 — Source Data for Figure 2 [file MSB-14-e8355-s003.zip › figure2-data/carpets_fmCherry-sfGFP-Bcd/emb2_r_rot0_carpet.tif]

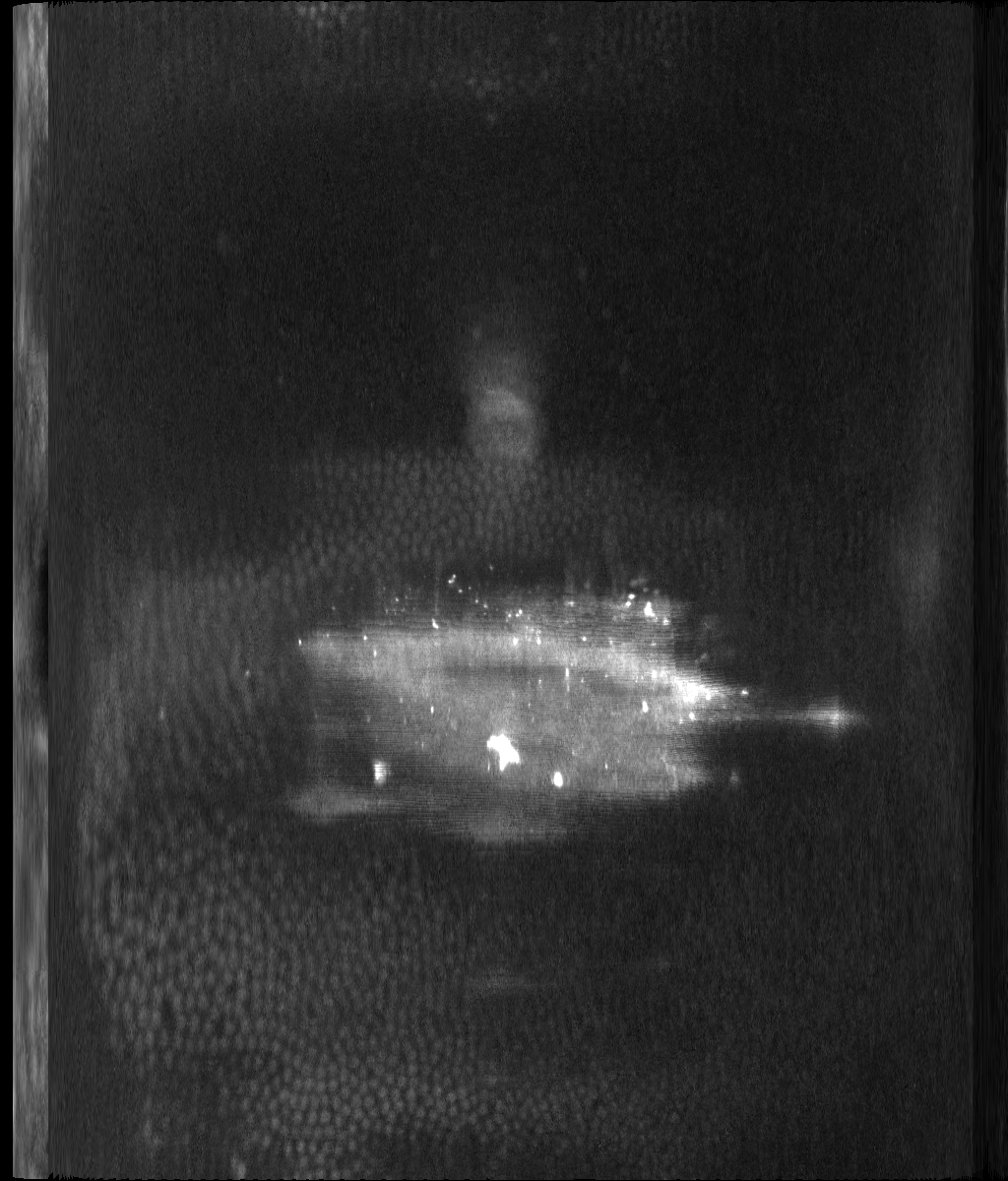

Supplement: Supplementary file 4 — Source Data for Figure 2 [file MSB-14-e8355-s003.zip › figure2-data/carpets_fmCherry-sfGFP-Bcd/emb2_r_rot1_carpet.tif]

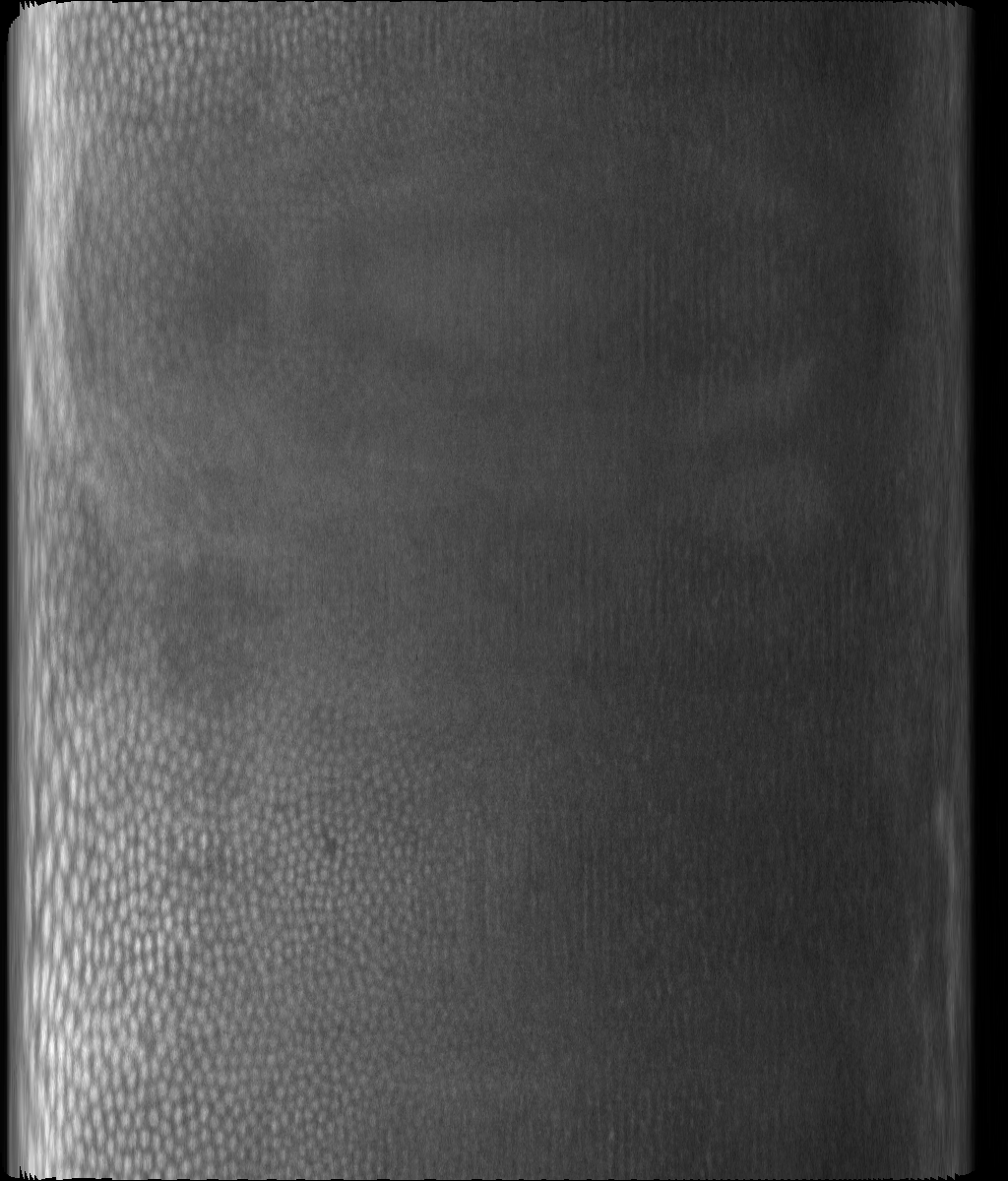

Supplement: Supplementary file 4 — Source Data for Figure 2 [file MSB-14-e8355-s003.zip › figure2-data/carpets_fmCherry-sfGFP-Bcd/emb4_g_fused_carpet.tif]

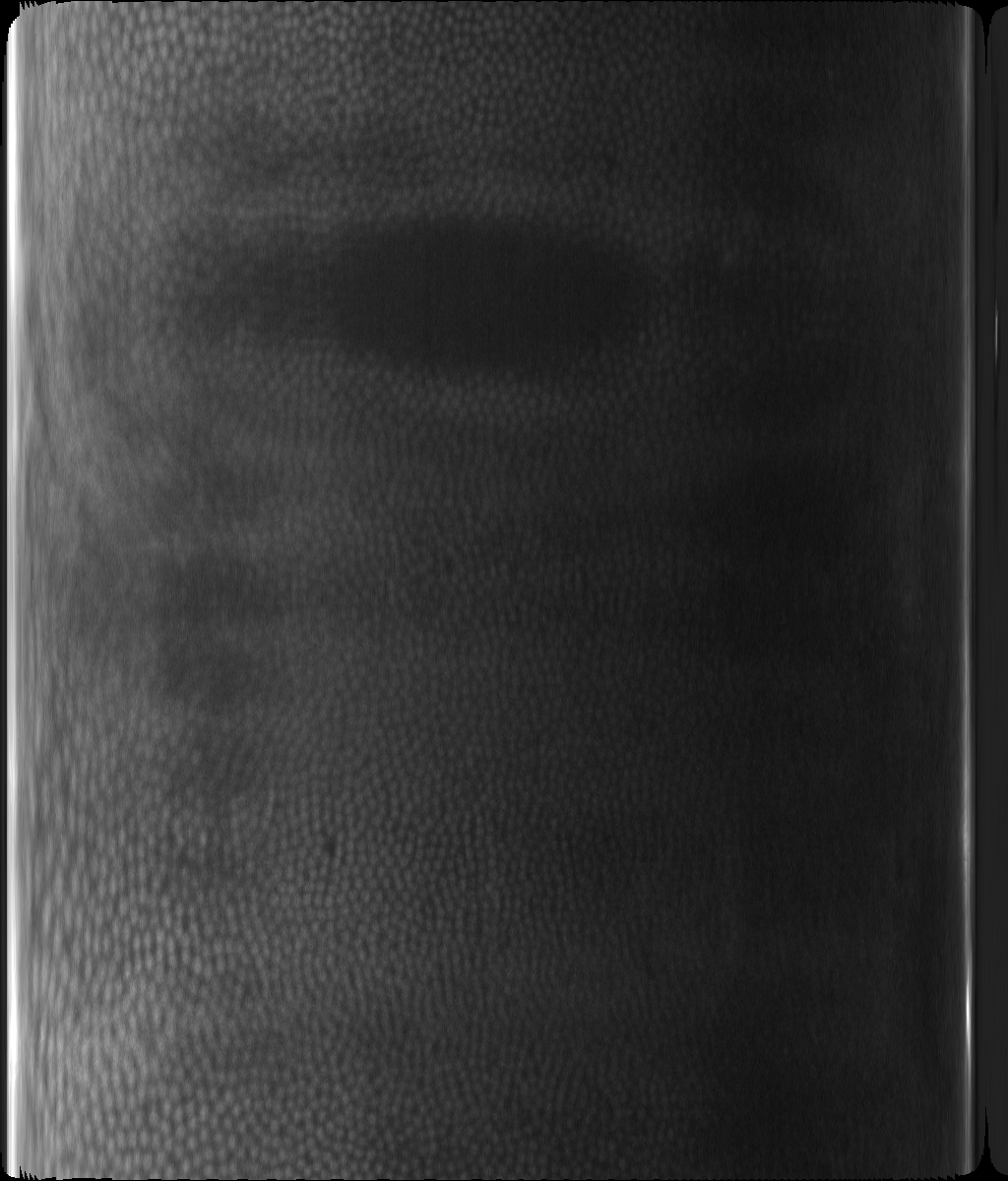

Supplement: Supplementary file 4 — Source Data for Figure 2 [file MSB-14-e8355-s003.zip › figure2-data/carpets_fmCherry-sfGFP-Bcd/emb4_r_fused_carpet.tif]

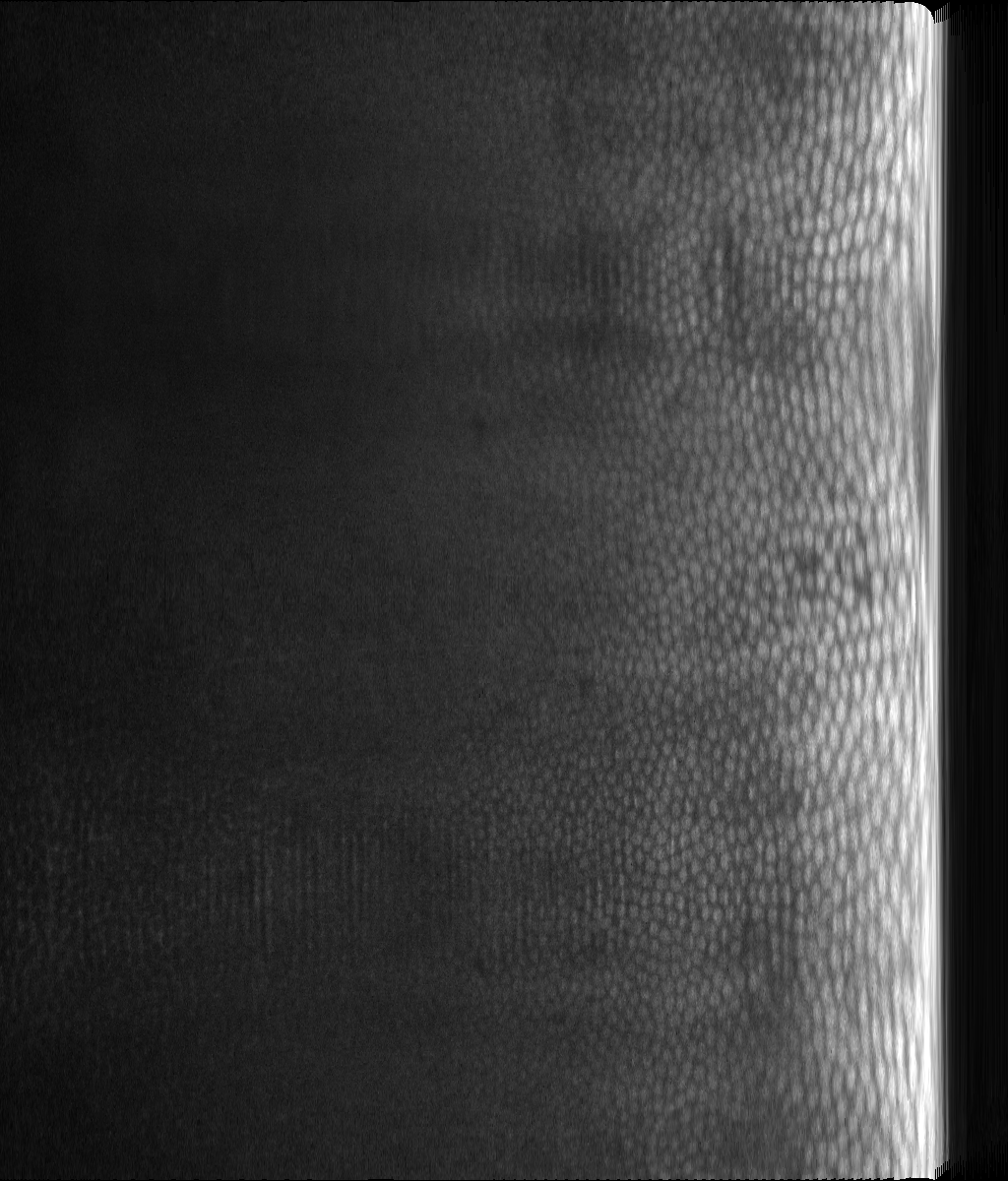

Supplement: Supplementary file 4 — Source Data for Figure 2 [file MSB-14-e8355-s003.zip › figure2-data/carpets_fmCherry-sfGFP-Bcd/emb6_g_rot0_carpet.tif]

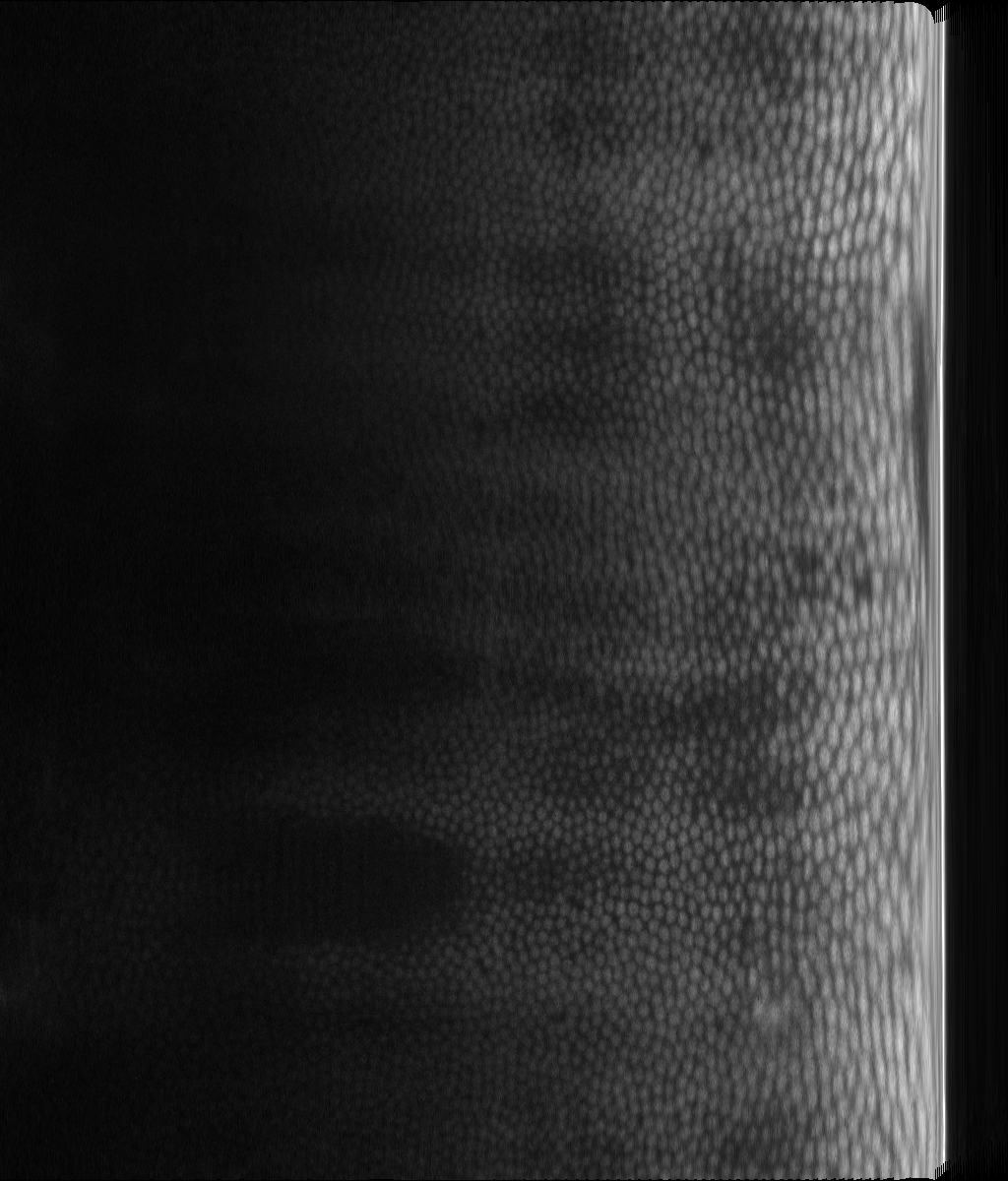

Supplement: Supplementary file 4 — Source Data for Figure 2 [file MSB-14-e8355-s003.zip › figure2-data/carpets_fmCherry-sfGFP-Bcd/emb6_r_rot0_carpet.tif]

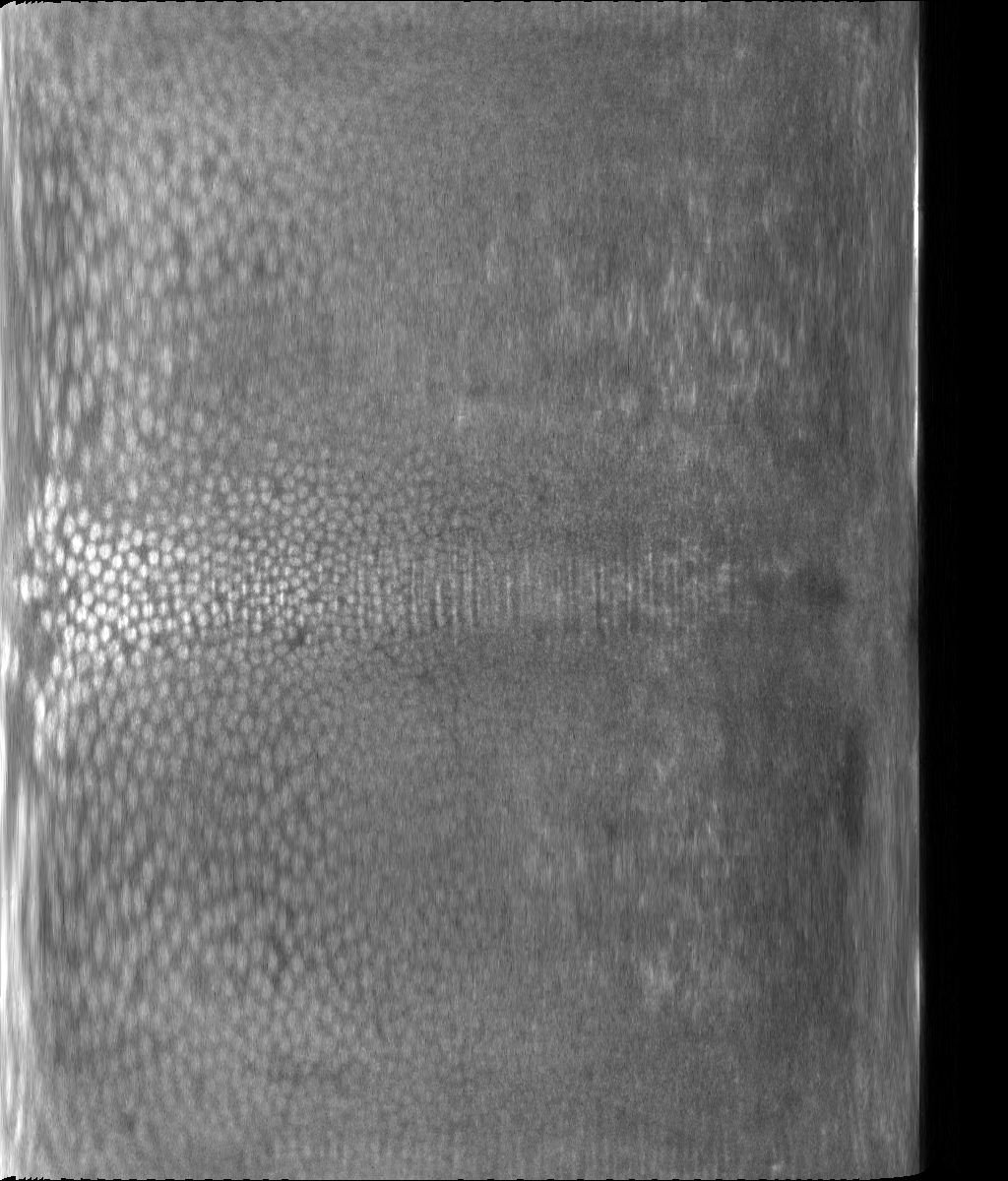

Supplement: Supplementary file 4 — Source Data for Figure 2 [file MSB-14-e8355-s003.zip › figure2-data/carpets_fmCherry-sfGFP-Bcd/emb7_g_fused_carpet.tif]

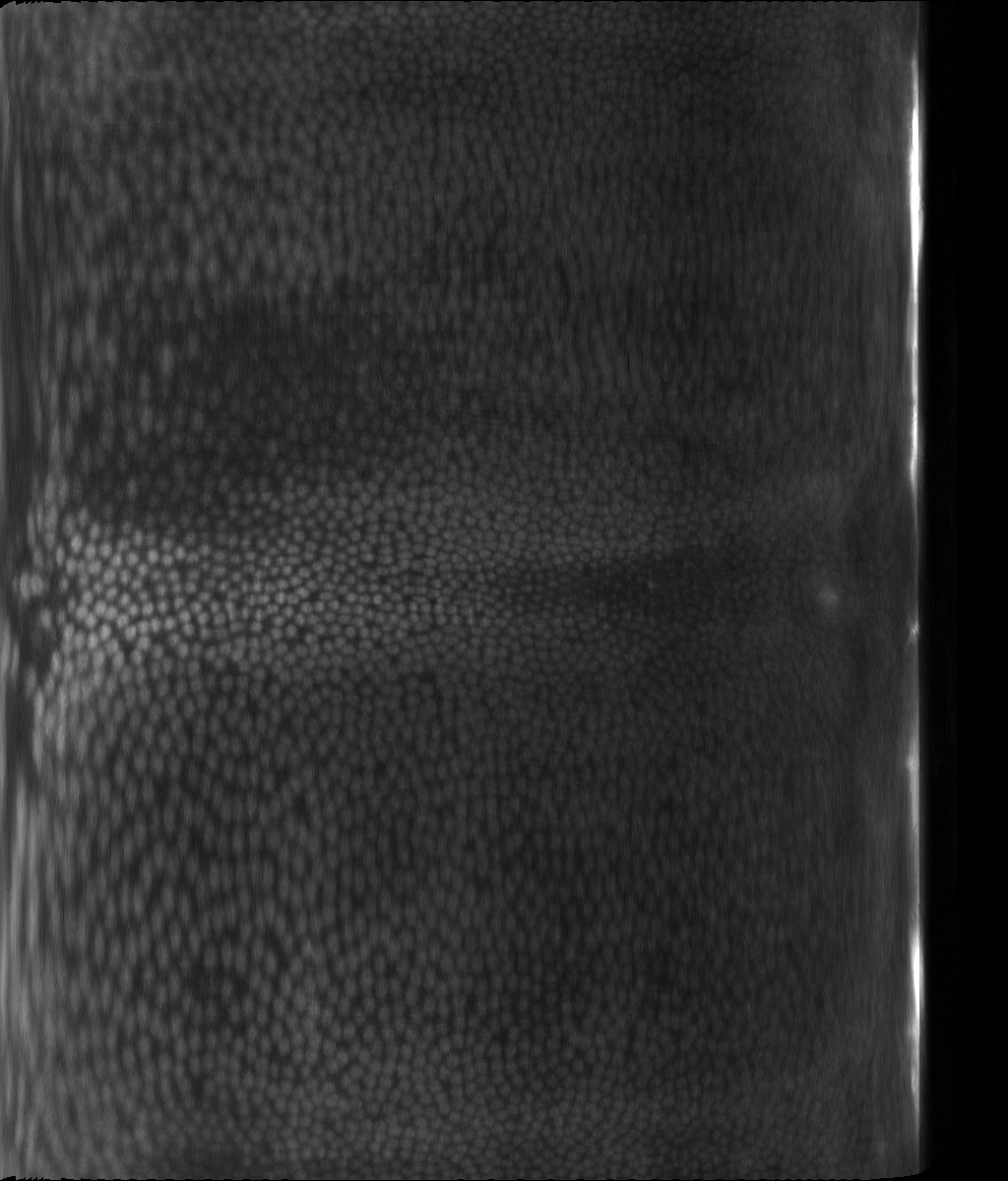

Supplement: Supplementary file 4 — Source Data for Figure 2 [file MSB-14-e8355-s003.zip › figure2-data/carpets_fmCherry-sfGFP-Bcd/emb7_r_fused_carpet.tif]

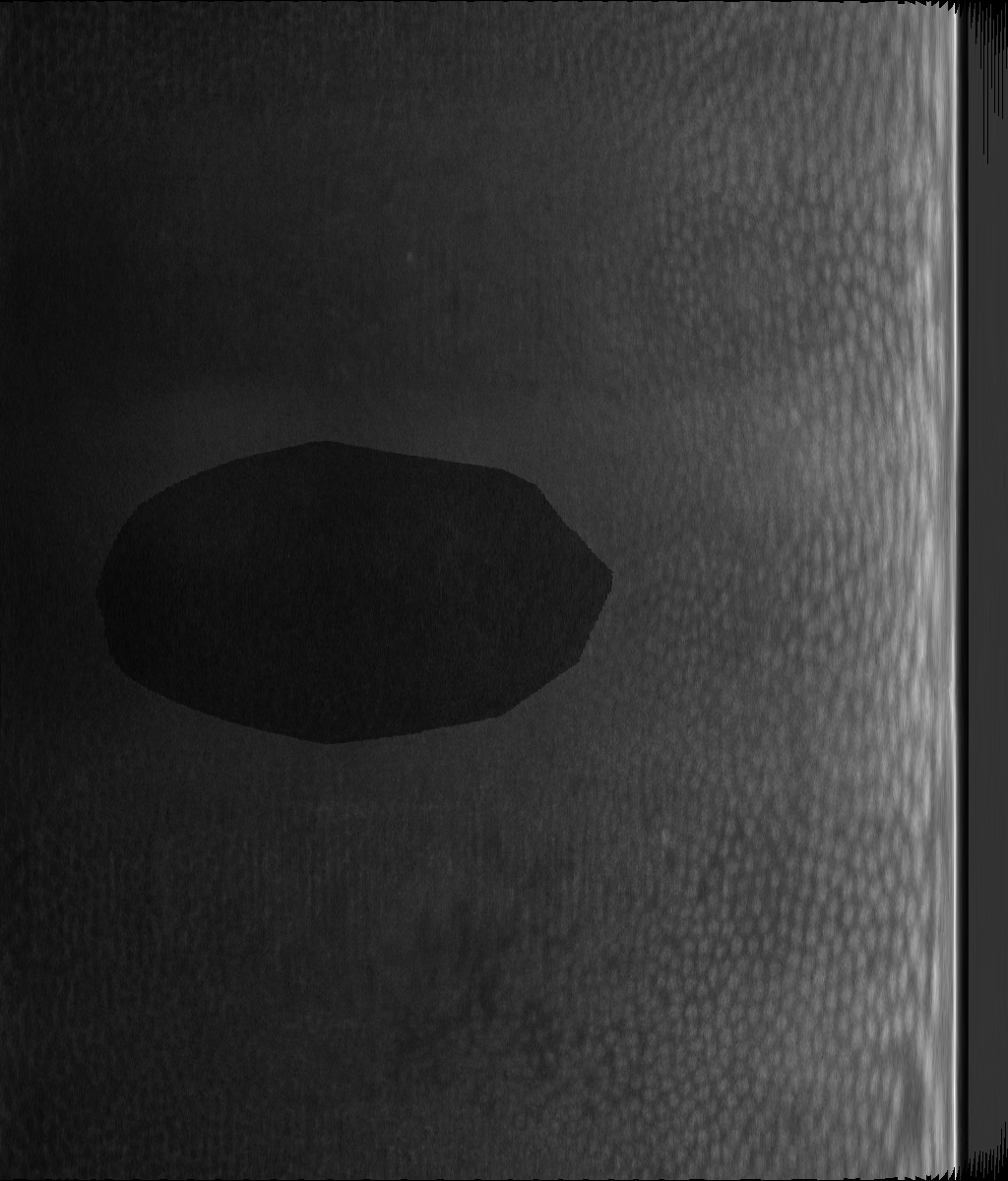

Supplement: Supplementary file 4 — Source Data for Figure 2 [file MSB-14-e8355-s003.zip › figure2-data/carpets_fmCherry-sfGFP-Bcd/emb9_g_fused_carpet.tif]

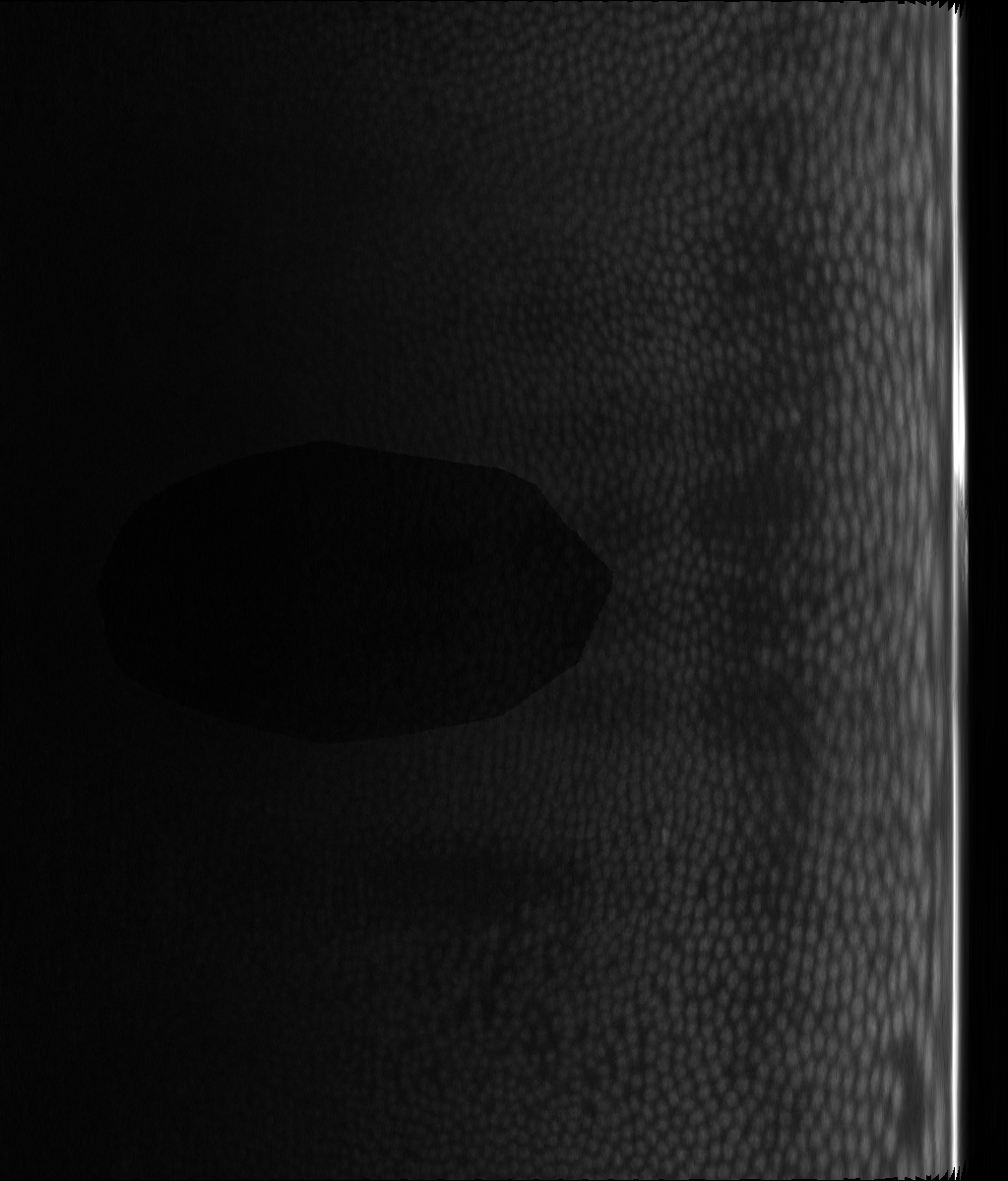

Supplement: Supplementary file 4 — Source Data for Figure 2 [file MSB-14-e8355-s003.zip › figure2-data/carpets_fmCherry-sfGFP-Bcd/emb9_r_fused_carpet.tif]

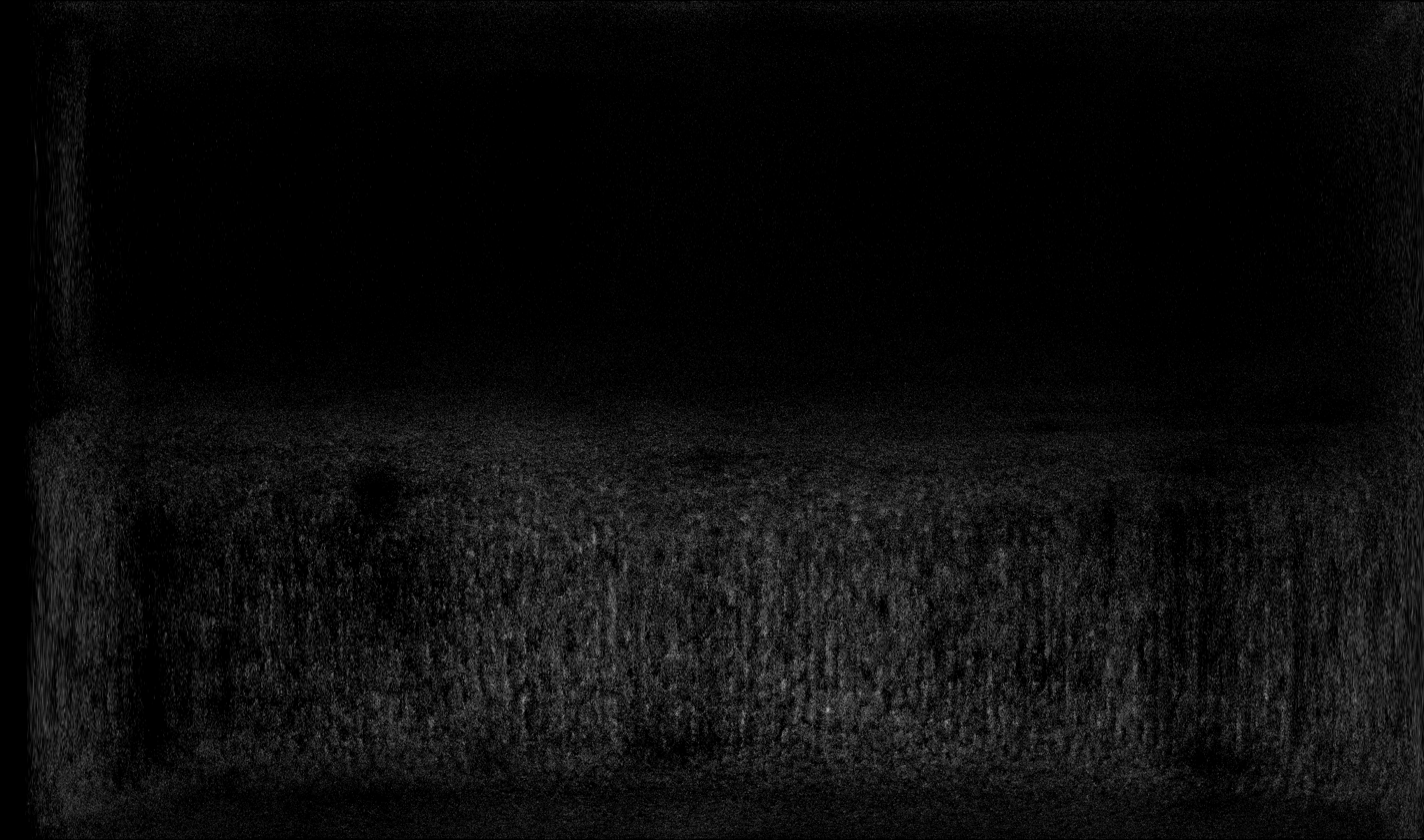

Supplement: Supplementary file 4 — Source Data for Figure 2 [file MSB-14-e8355-s003.zip › figure2-data/carpets_mCherry-sfGFP/con10_rot0_right_g_carpet.tif]

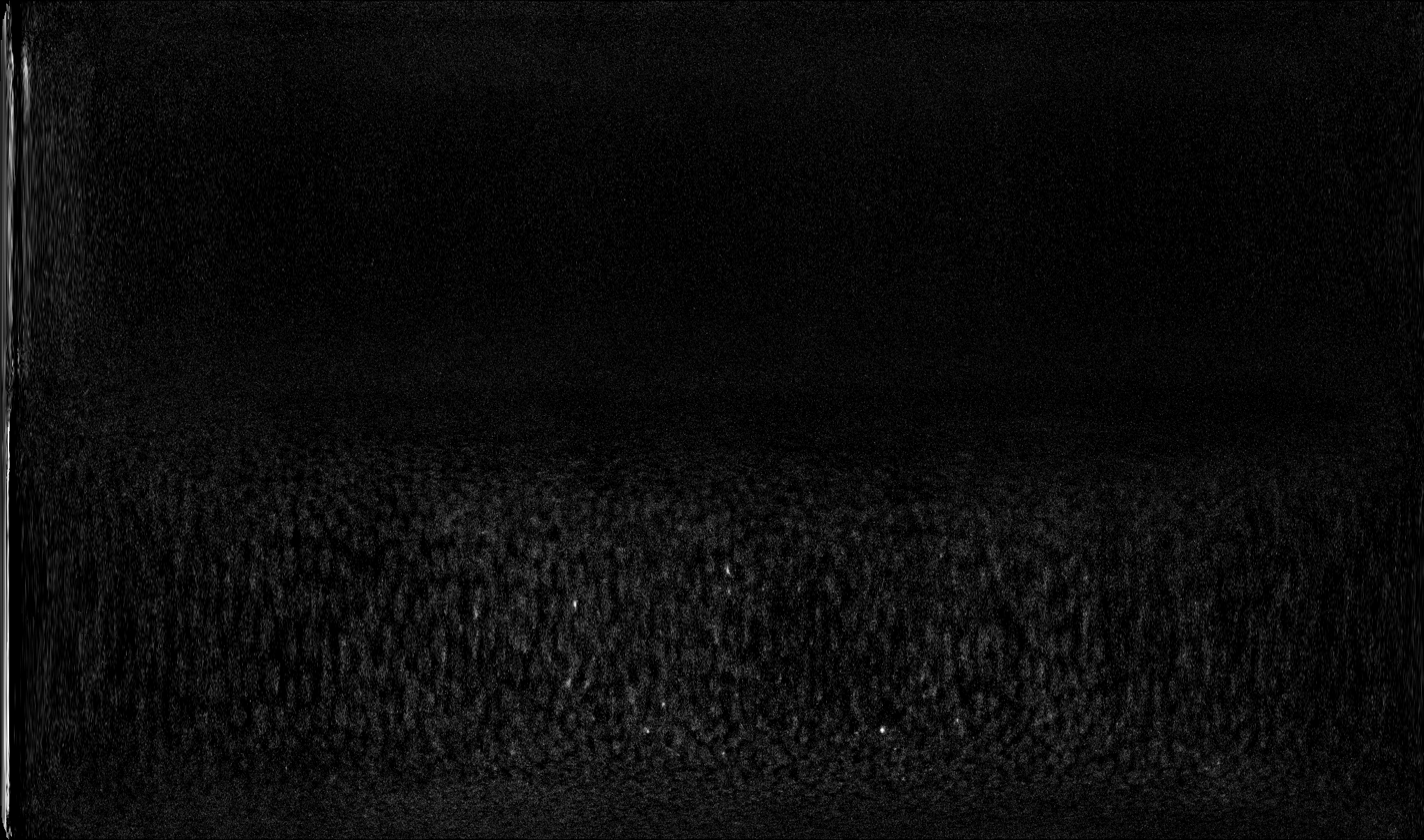

Supplement: Supplementary file 4 — Source Data for Figure 2 [file MSB-14-e8355-s003.zip › figure2-data/carpets_mCherry-sfGFP/con10_rot0_right_r_carpet.tif]

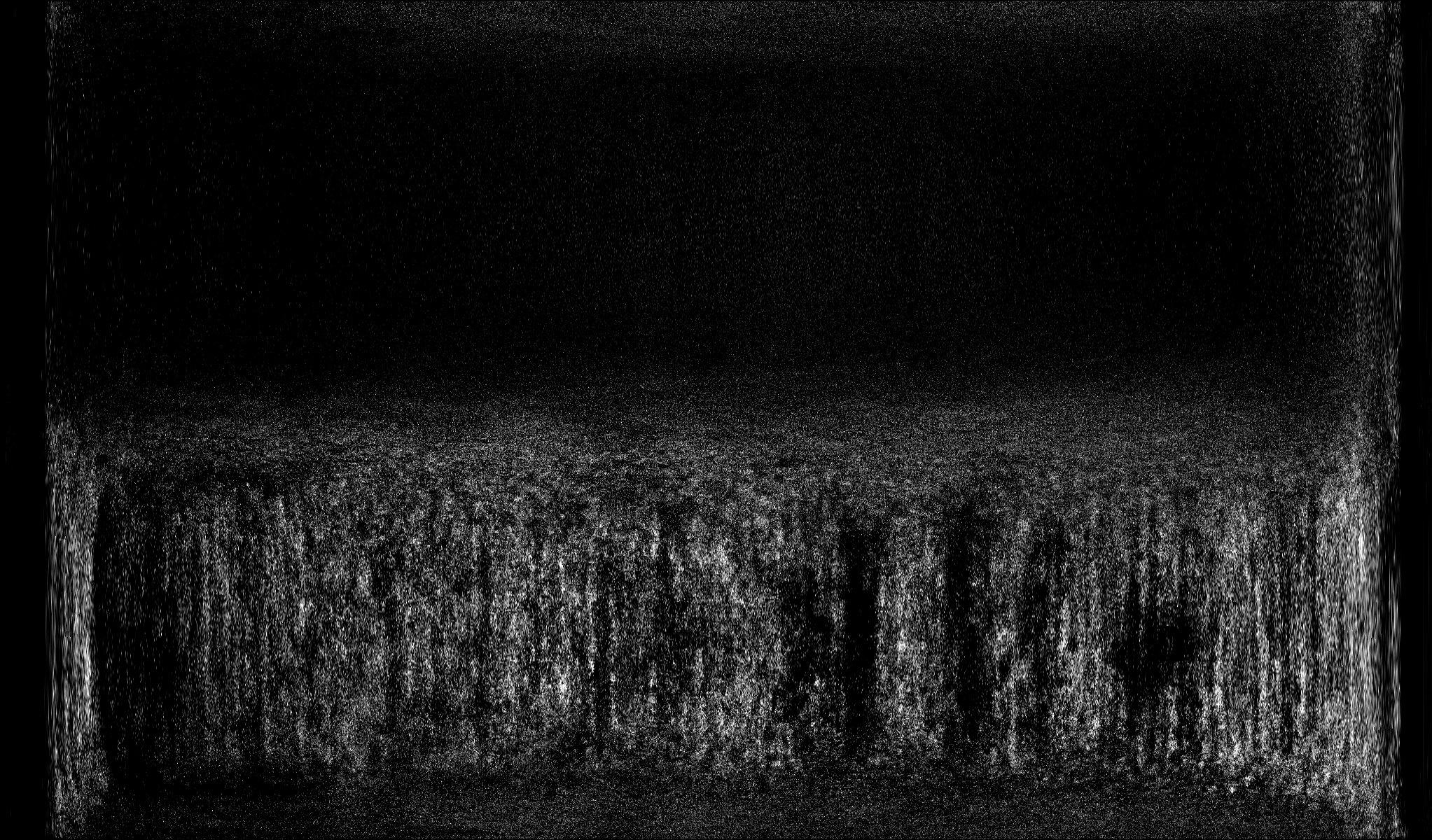

Supplement: Supplementary file 4 — Source Data for Figure 2 [file MSB-14-e8355-s003.zip › figure2-data/carpets_mCherry-sfGFP/con12_rot0_right_g_carpet.tif]

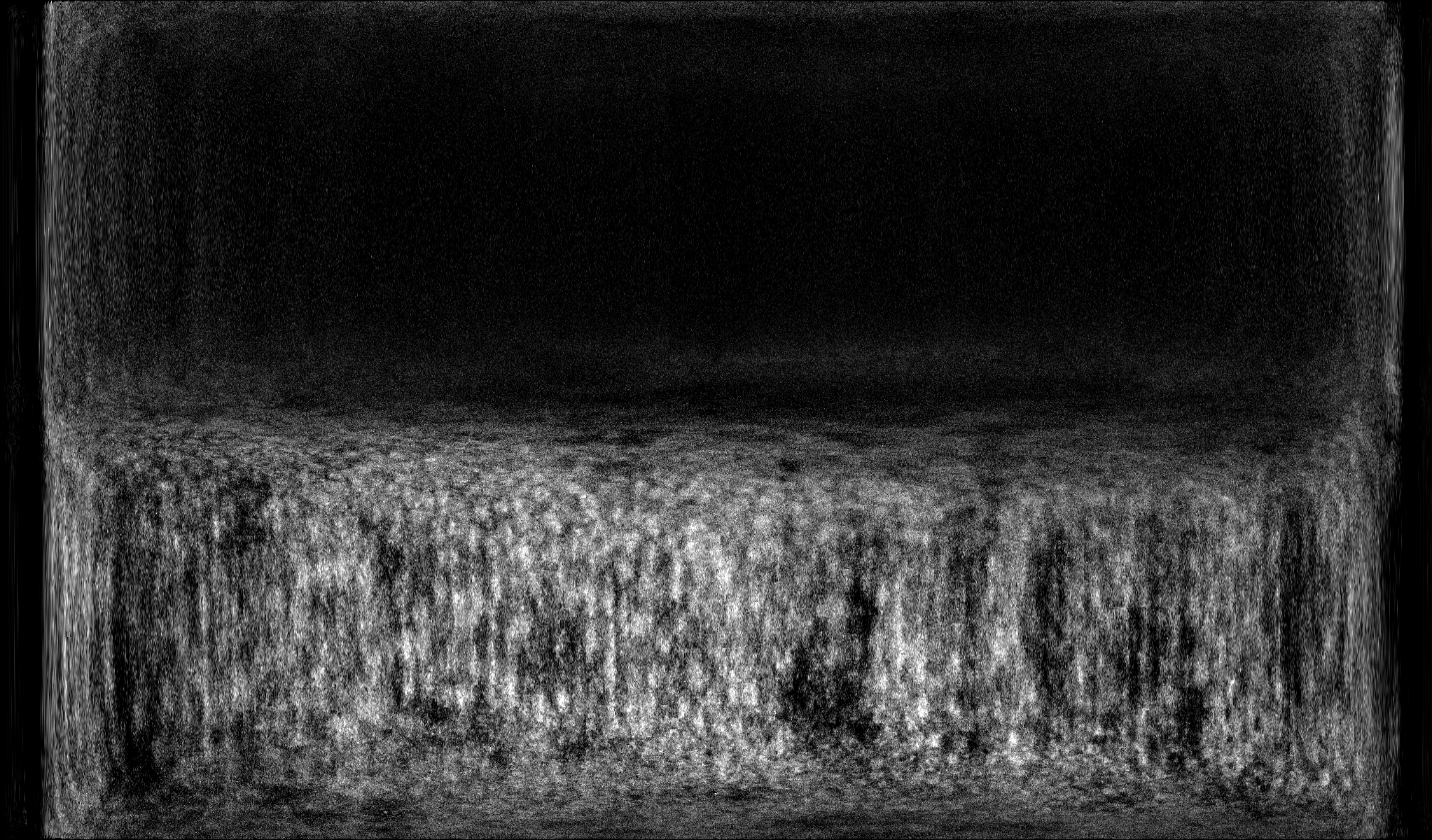

Supplement: Supplementary file 4 — Source Data for Figure 2 [file MSB-14-e8355-s003.zip › figure2-data/carpets_mCherry-sfGFP/con12_rot0_right_r_carpet.tif]

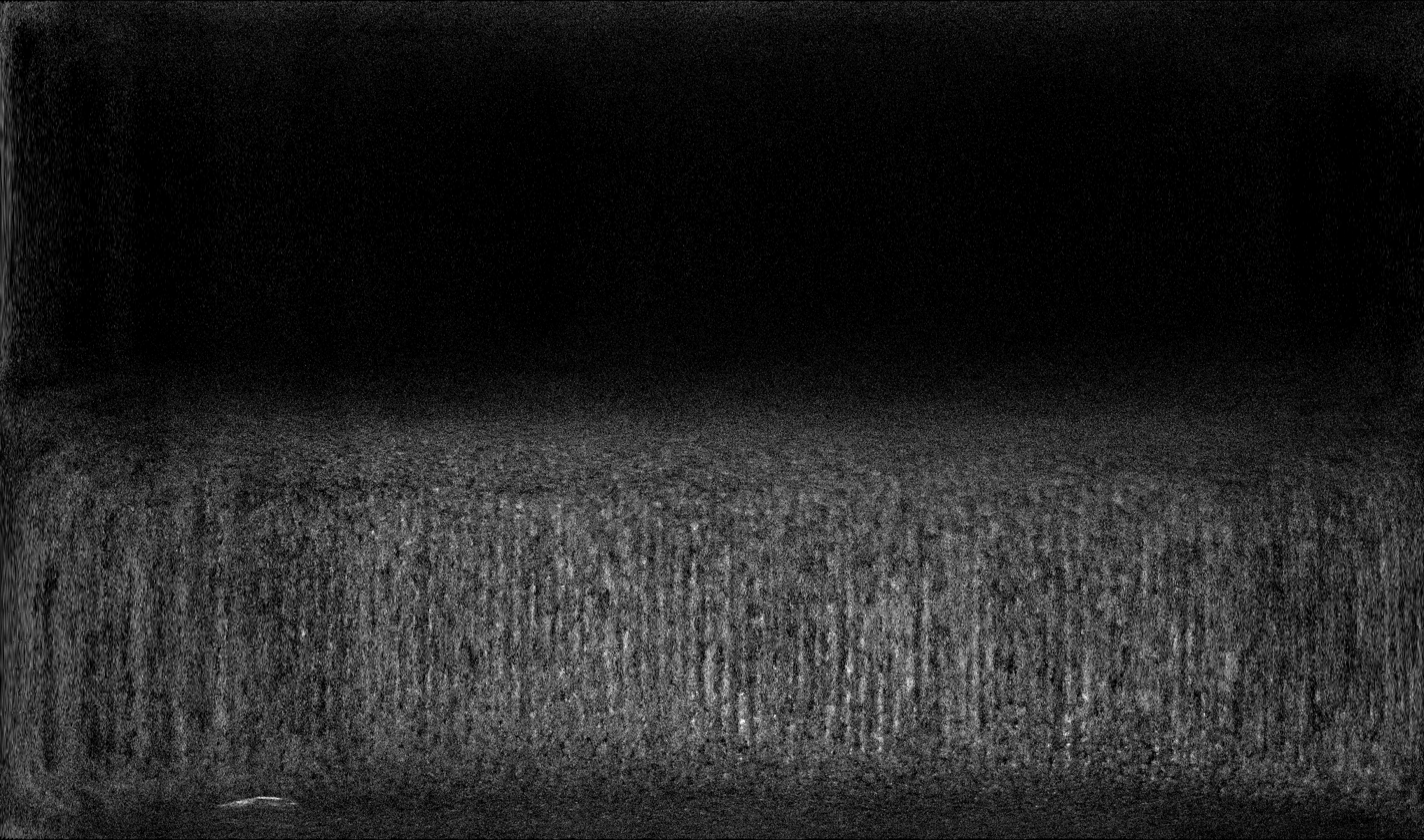

Supplement: Supplementary file 4 — Source Data for Figure 2 [file MSB-14-e8355-s003.zip › figure2-data/carpets_mCherry-sfGFP/con13_rot0_right_g_carpet.tif]

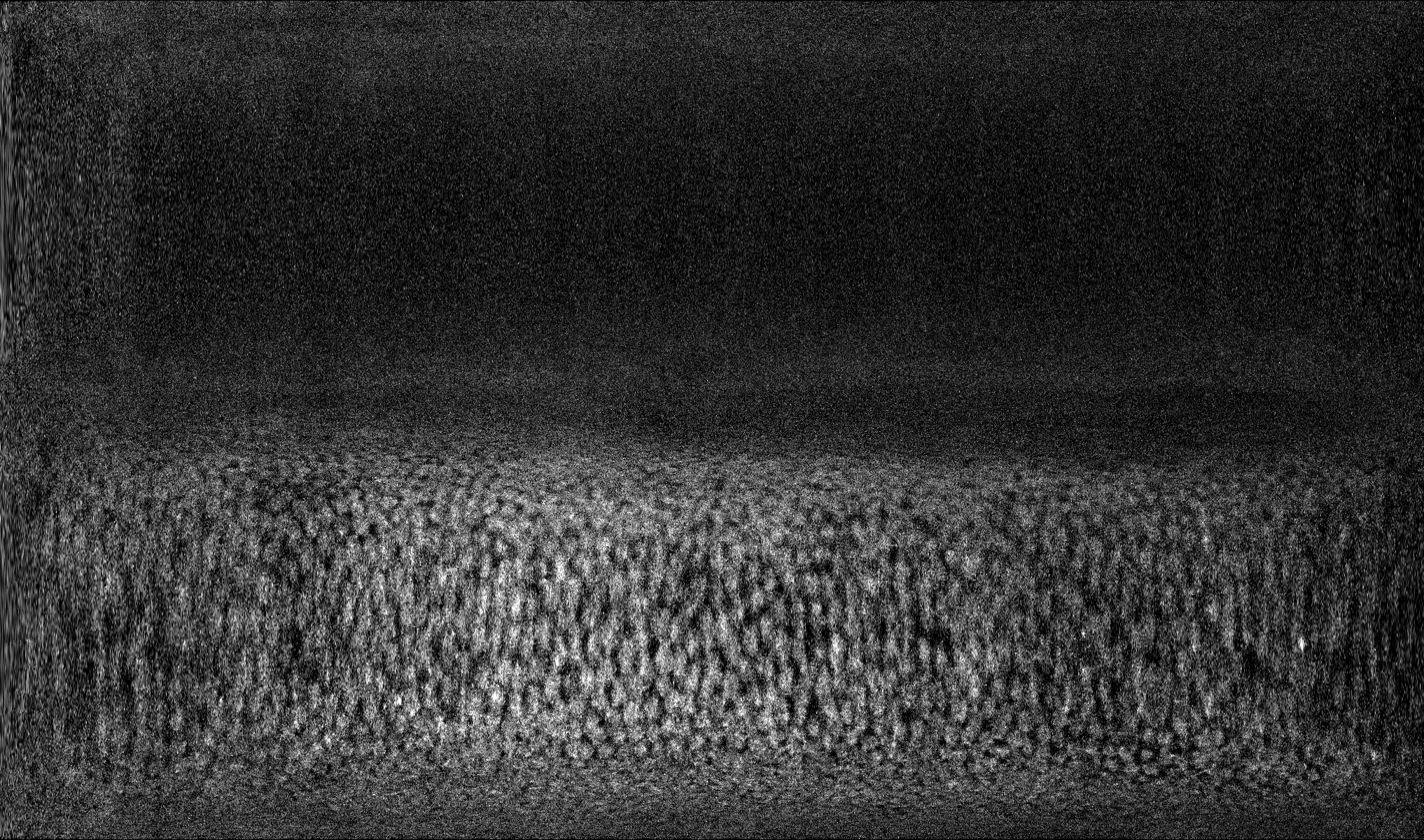

Supplement: Supplementary file 4 — Source Data for Figure 2 [file MSB-14-e8355-s003.zip › figure2-data/carpets_mCherry-sfGFP/con13_rot0_right_r_carpet.tif]

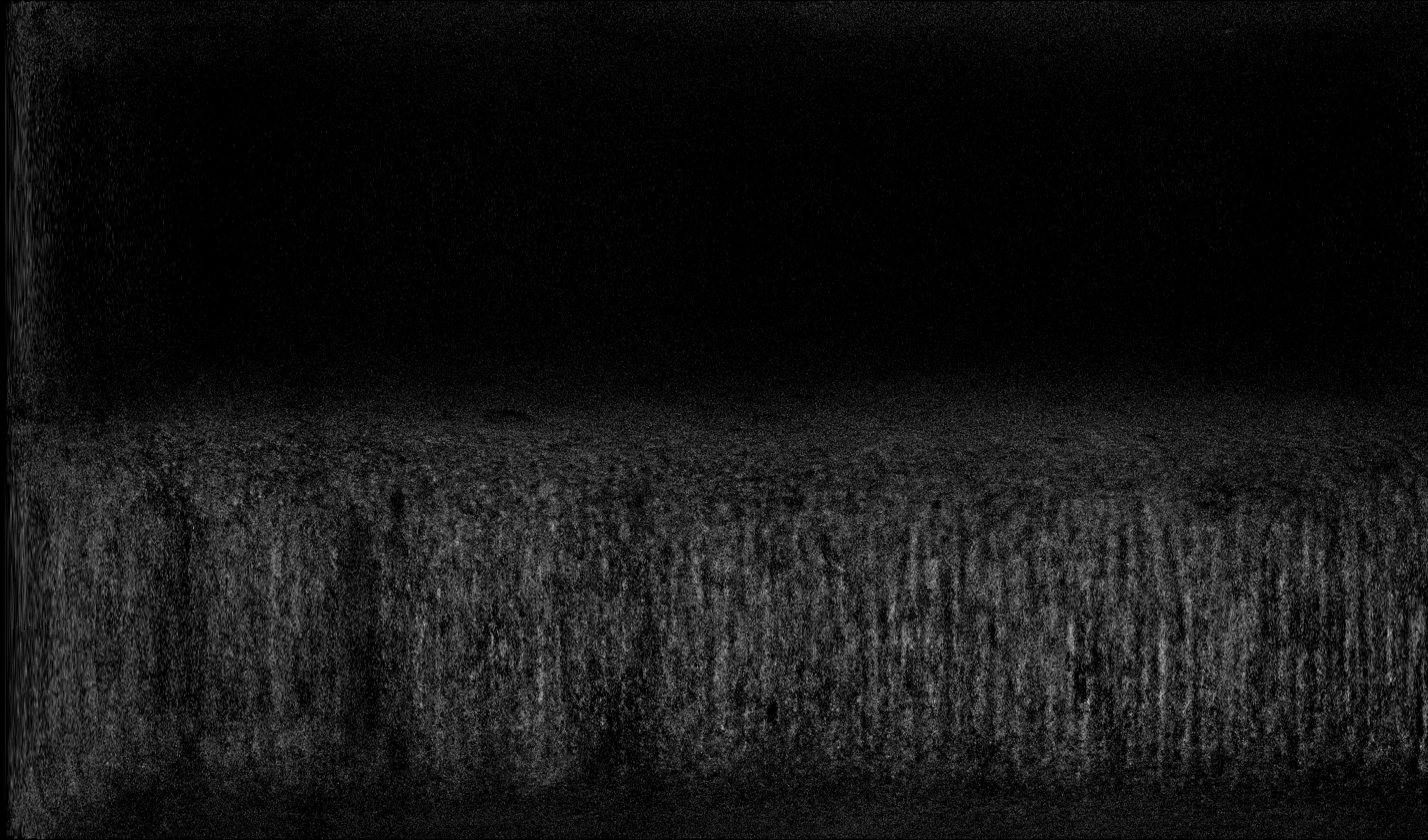

Supplement: Supplementary file 4 — Source Data for Figure 2 [file MSB-14-e8355-s003.zip › figure2-data/carpets_mCherry-sfGFP/con14_rot0_right_g_carpet.tif]

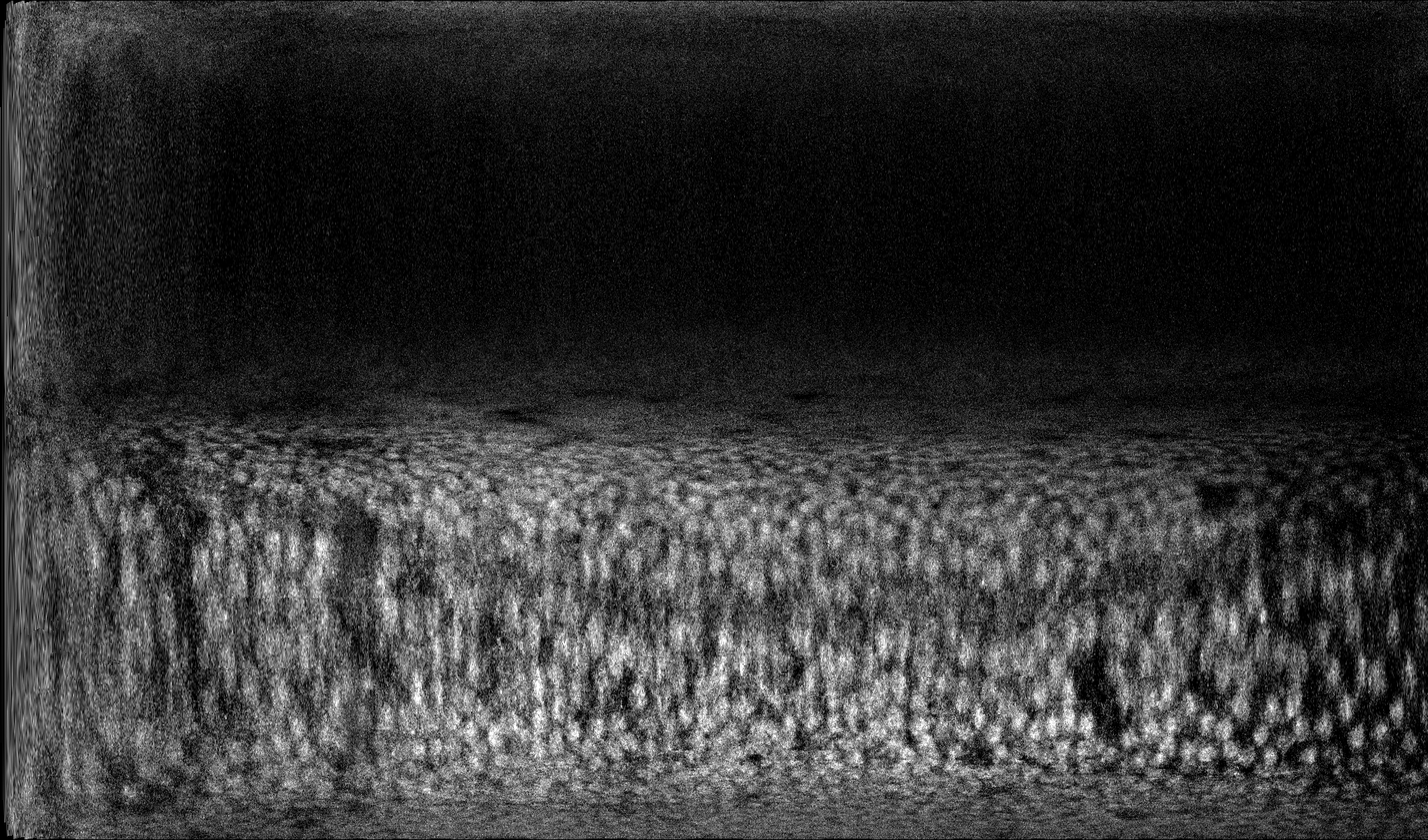

Supplement: Supplementary file 4 — Source Data for Figure 2 [file MSB-14-e8355-s003.zip › figure2-data/carpets_mCherry-sfGFP/con14_rot0_right_r_carpet.tif]

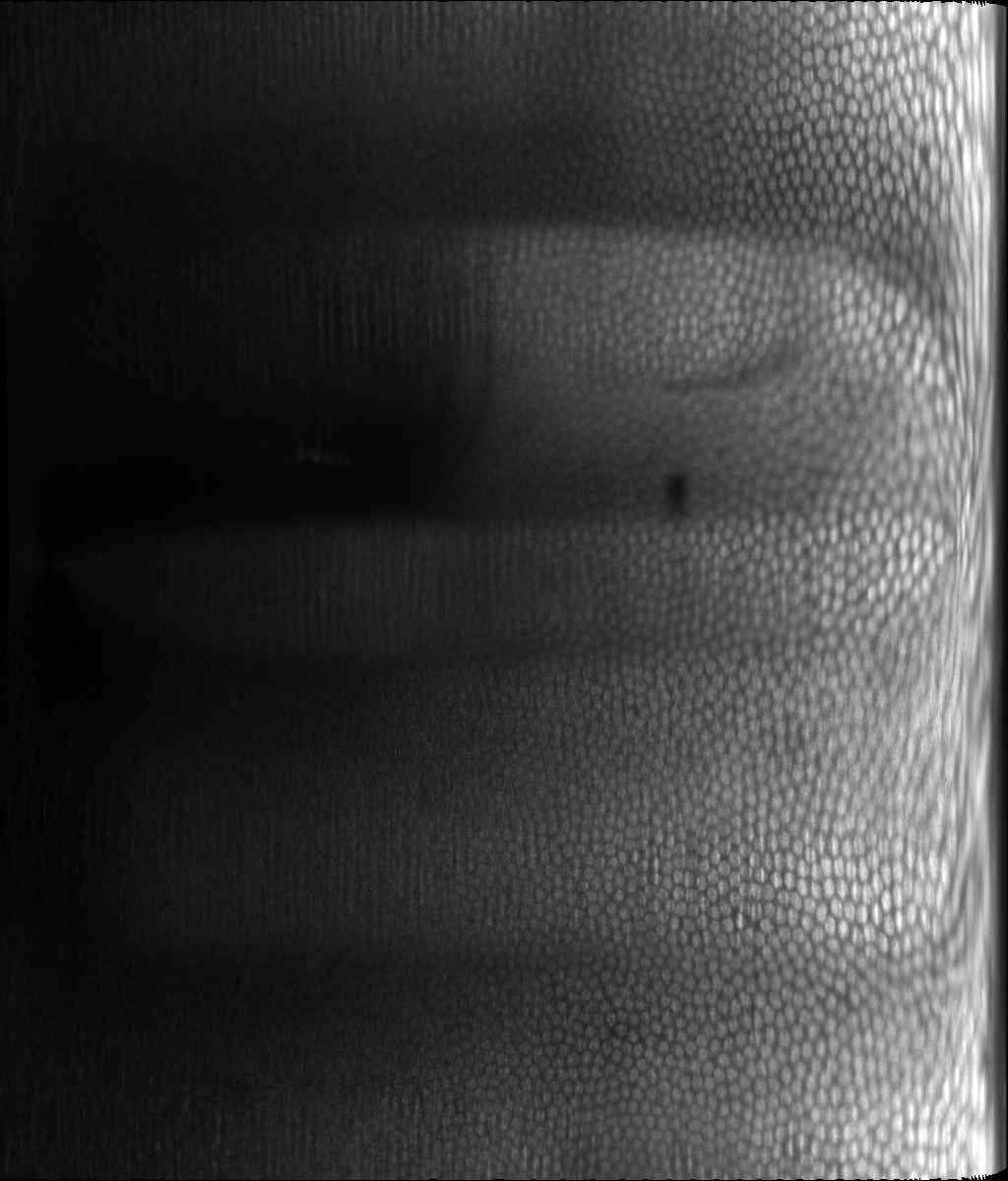

Supplement: Supplementary file 4 — Source Data for Figure 2 [file MSB-14-e8355-s003.zip › figure2-data/carpets_mCherry-sfGFP-Bcd/wt1_fused_g_carpet.tif]

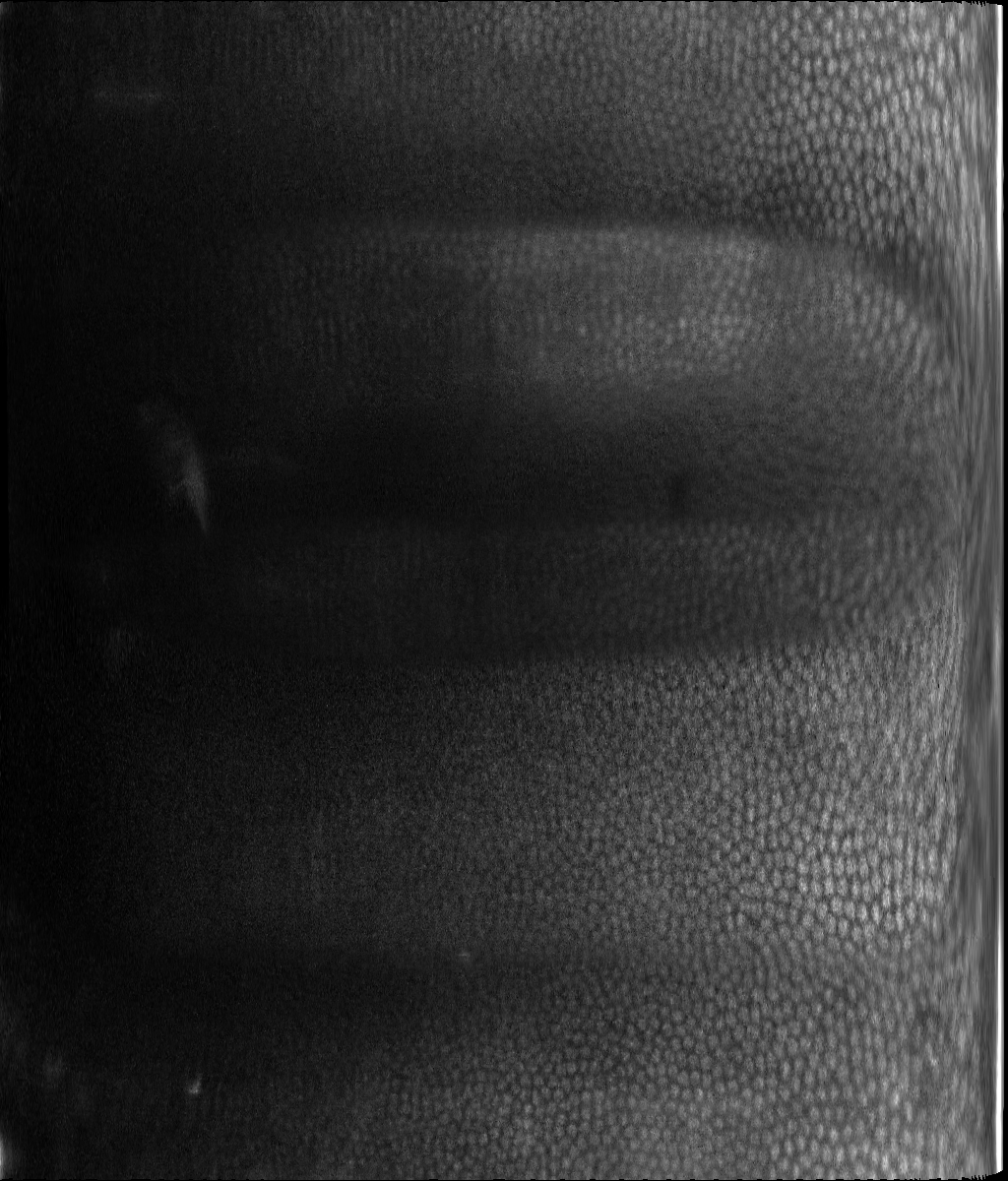

Supplement: Supplementary file 4 — Source Data for Figure 2 [file MSB-14-e8355-s003.zip › figure2-data/carpets_mCherry-sfGFP-Bcd/wt1_fused_r_carpet.tif]

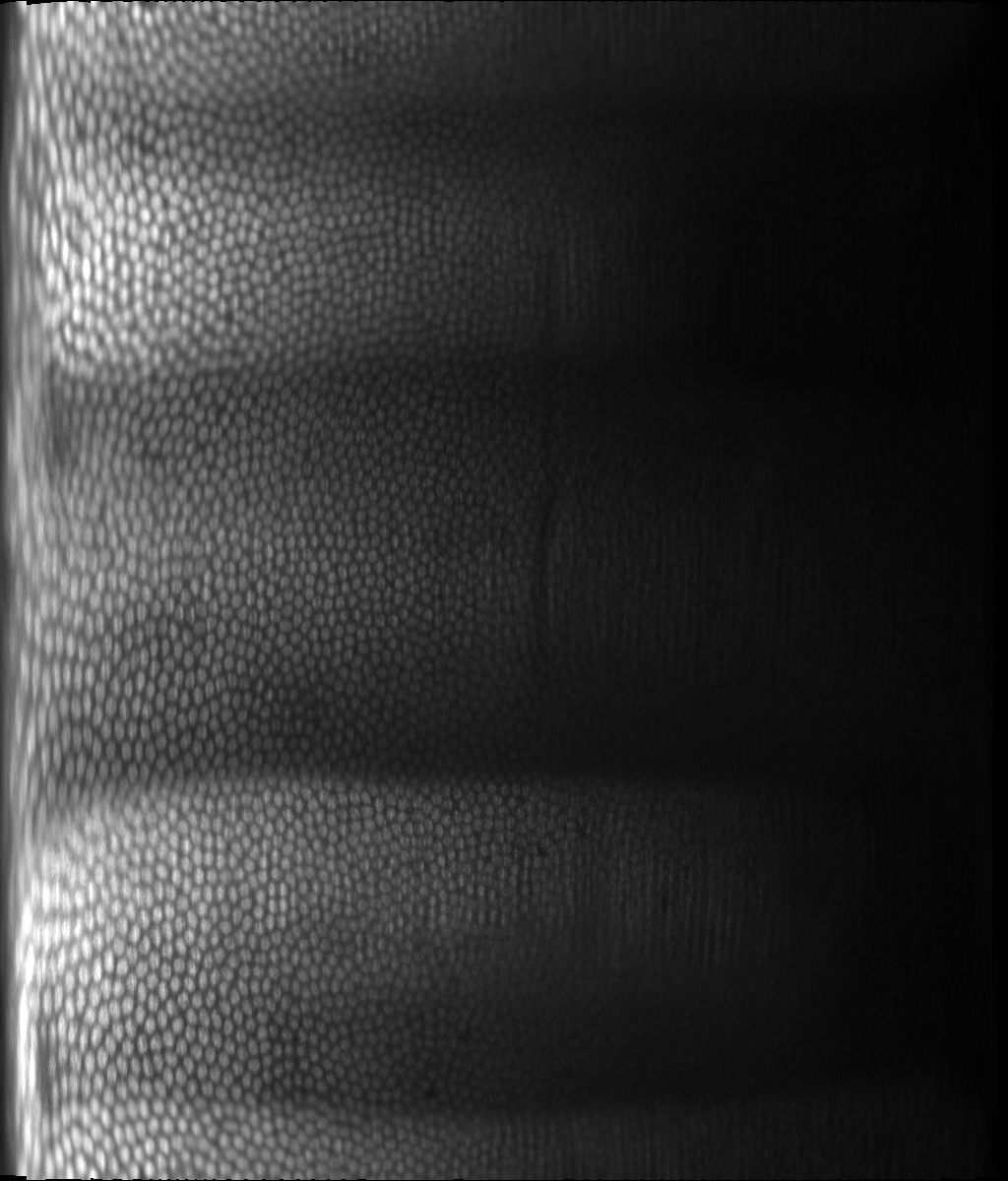

Supplement: Supplementary file 4 — Source Data for Figure 2 [file MSB-14-e8355-s003.zip › figure2-data/carpets_mCherry-sfGFP-Bcd/wt2_fused_g_carpet.tif]

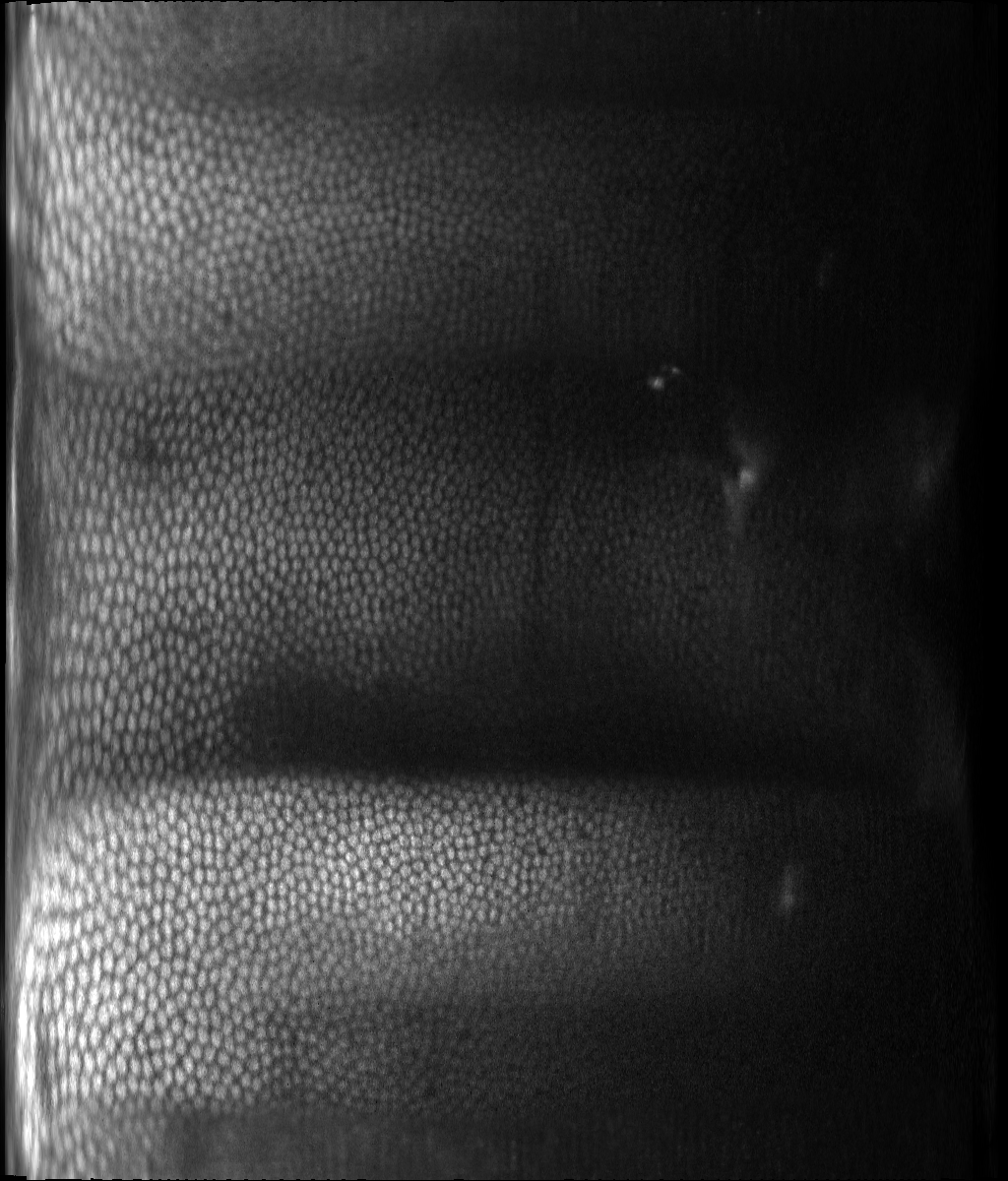

Supplement: Supplementary file 4 — Source Data for Figure 2 [file MSB-14-e8355-s003.zip › figure2-data/carpets_mCherry-sfGFP-Bcd/wt2_fused_r_carpet.tif]

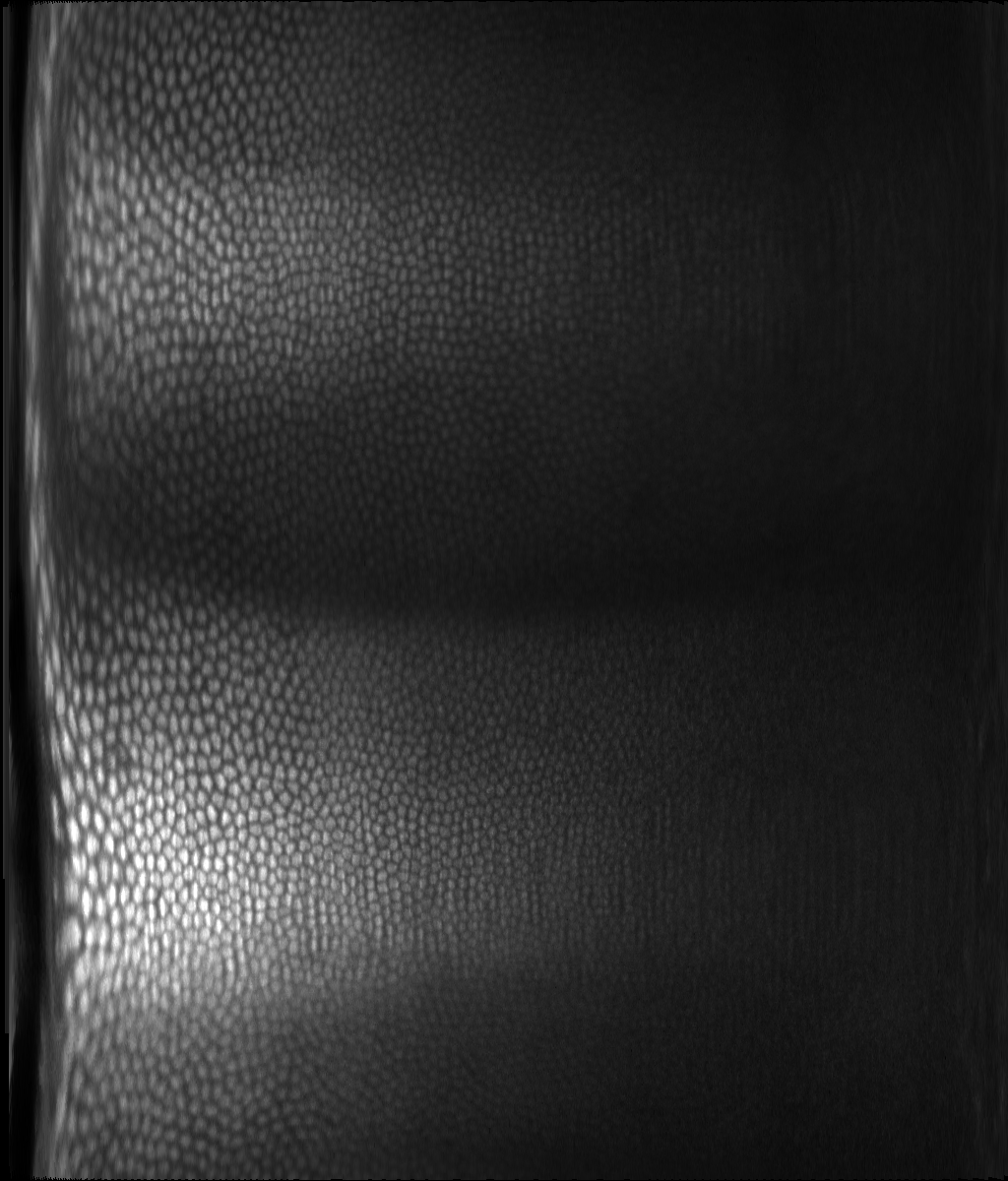

Supplement: Supplementary file 4 — Source Data for Figure 2 [file MSB-14-e8355-s003.zip › figure2-data/carpets_mCherry-sfGFP-Bcd/wt3_rot0_g_carpet.tif]

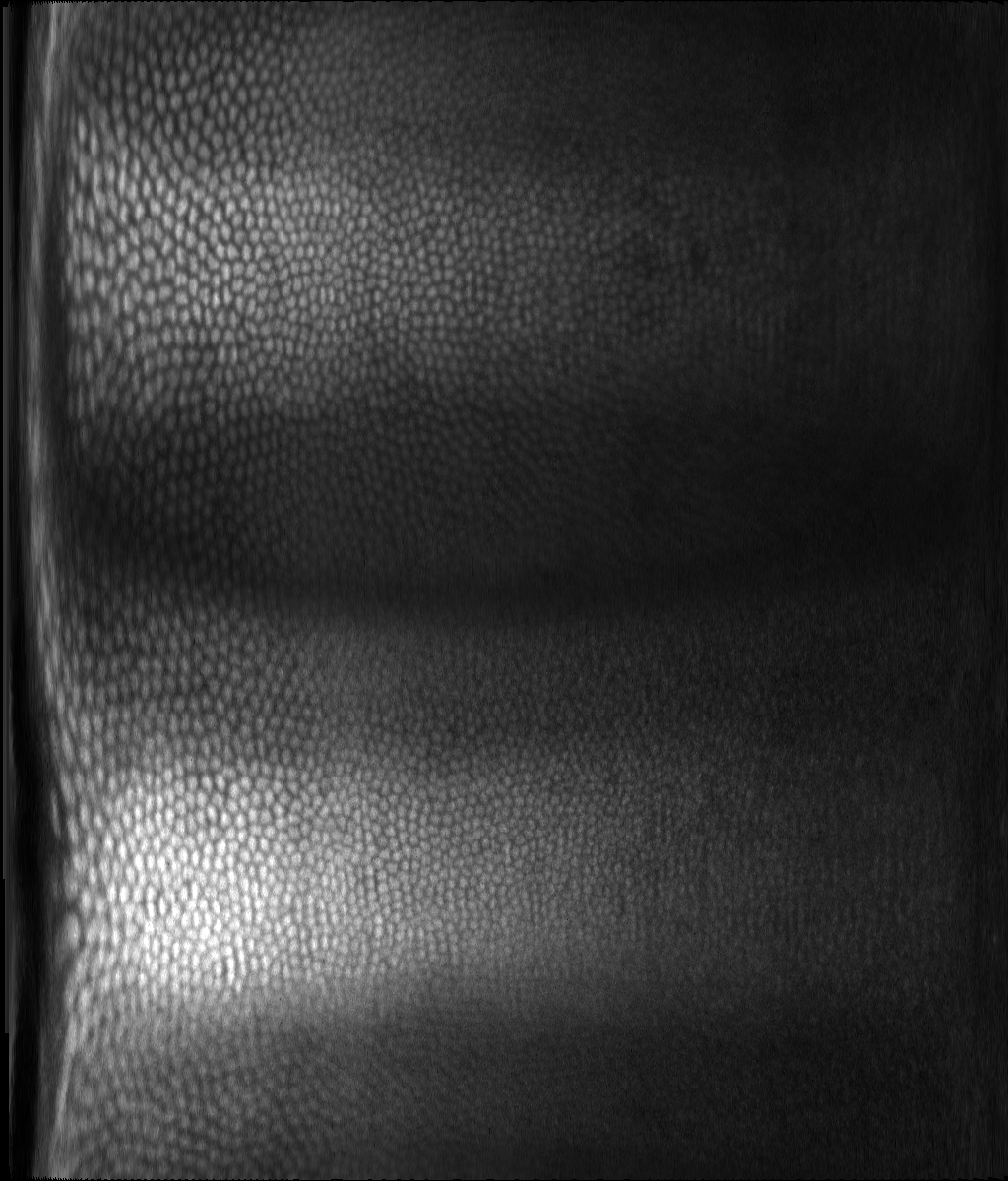

Supplement: Supplementary file 4 — Source Data for Figure 2 [file MSB-14-e8355-s003.zip › figure2-data/carpets_mCherry-sfGFP-Bcd/wt3_rot0_r_carpet.tif]

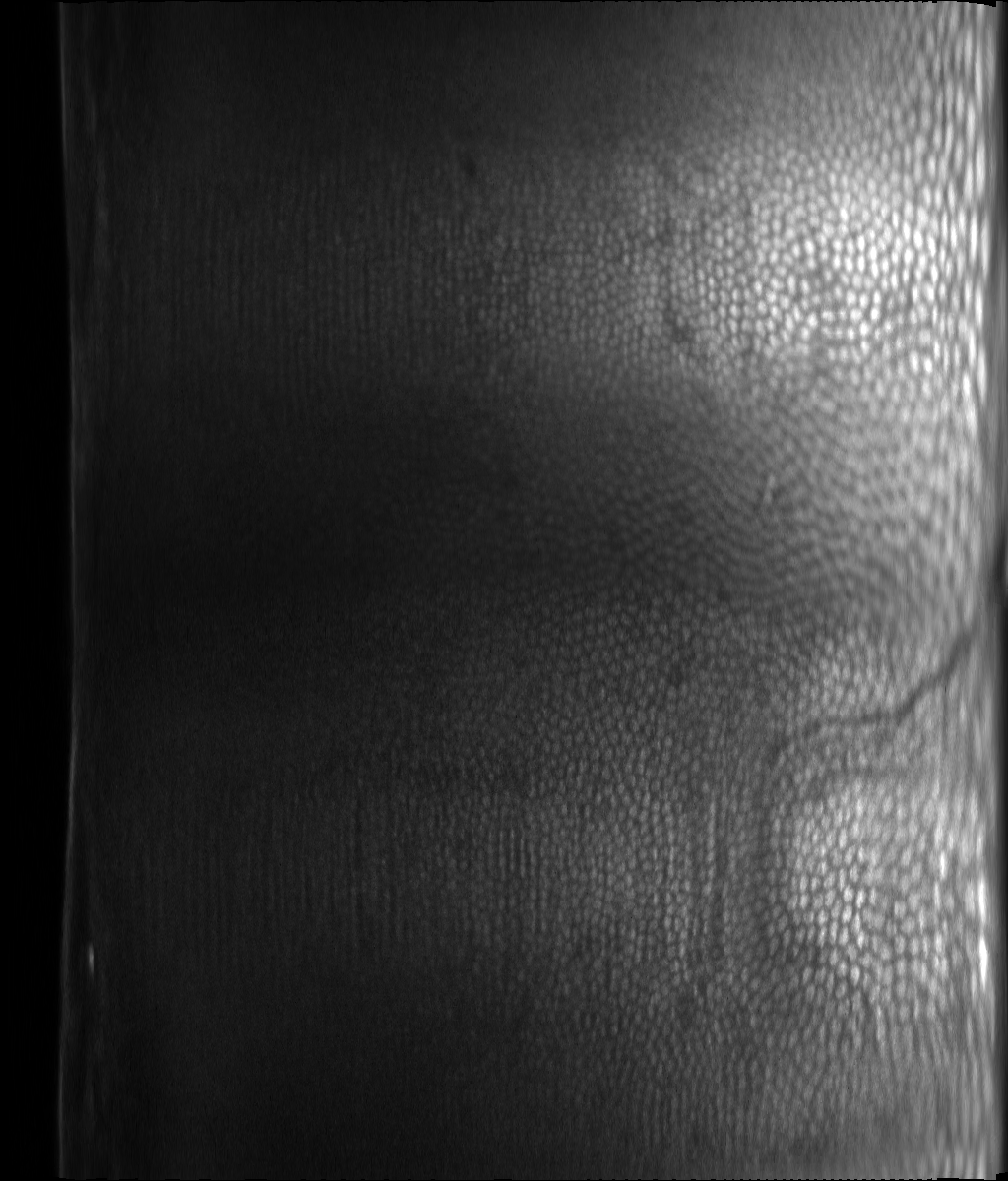

Supplement: Supplementary file 4 — Source Data for Figure 2 [file MSB-14-e8355-s003.zip › figure2-data/carpets_mCherry-sfGFP-Bcd/wt4_fused_g_carpet.tif]

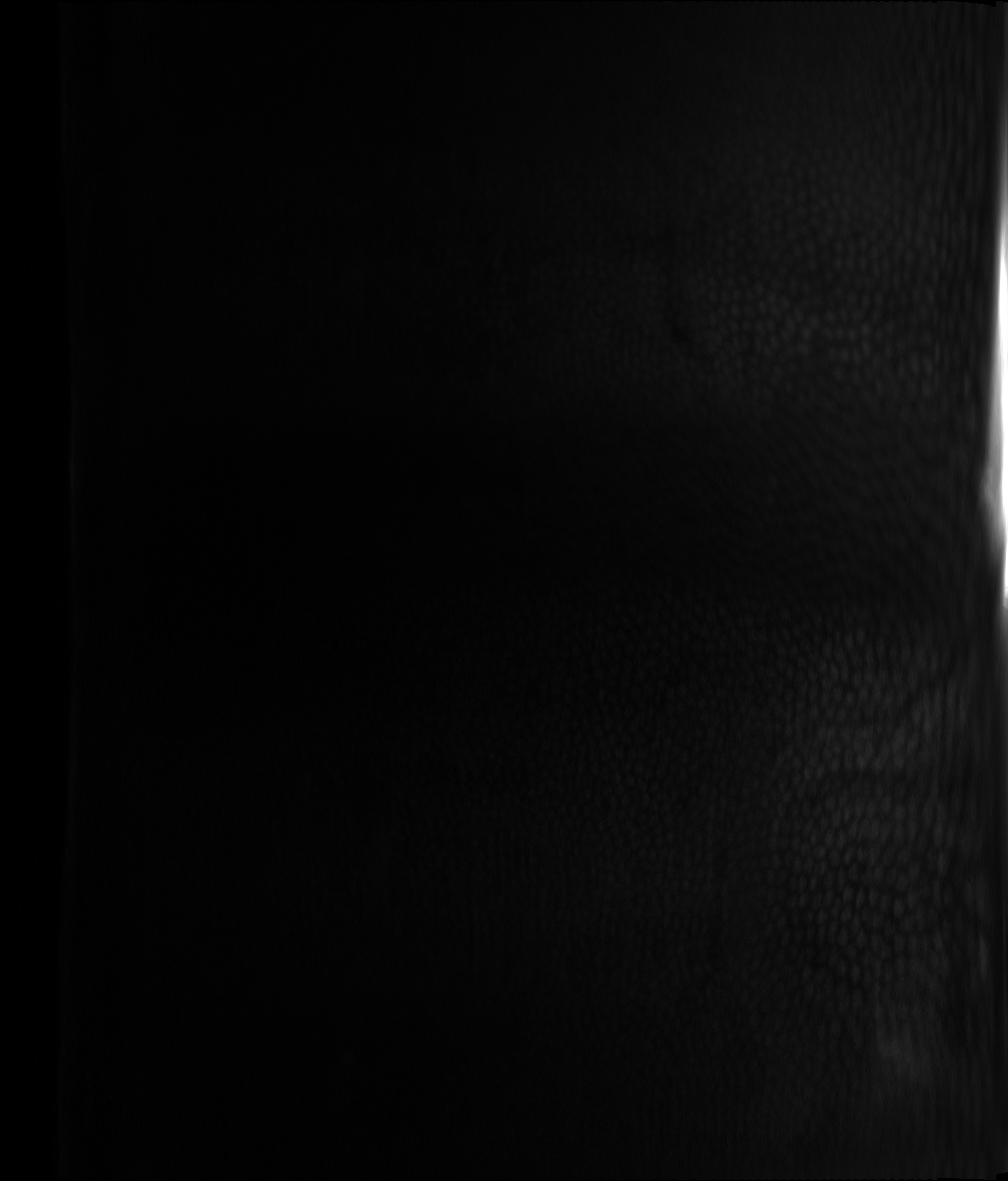

Supplement: Supplementary file 4 — Source Data for Figure 2 [file MSB-14-e8355-s003.zip › figure2-data/carpets_mCherry-sfGFP-Bcd/wt4_fused_r_carpet.tif]

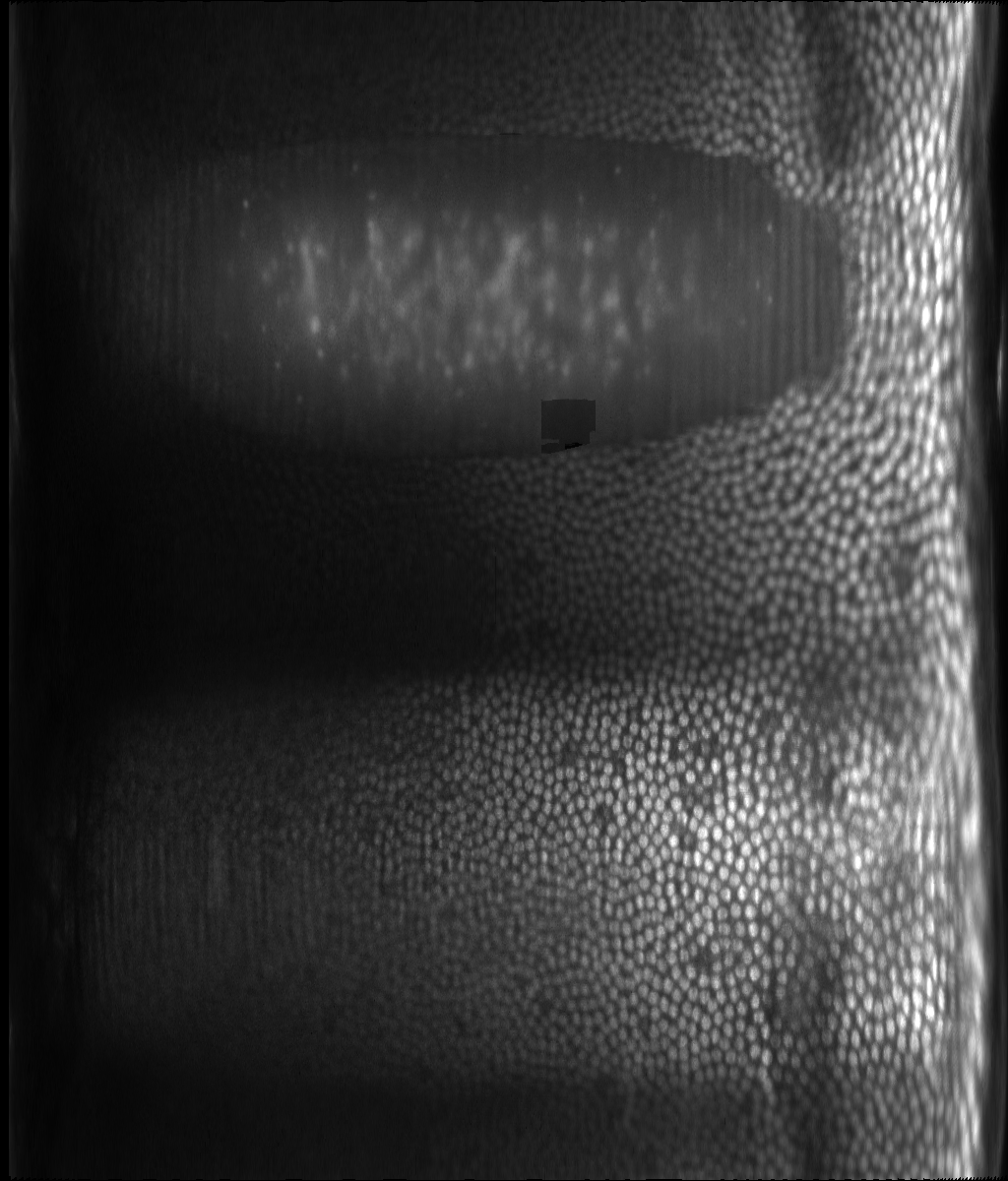

Supplement: Supplementary file 4 — Source Data for Figure 2 [file MSB-14-e8355-s003.zip › figure2-data/carpets_mCherry-sfGFP-Bcd/wt5_g_carpet.tif]

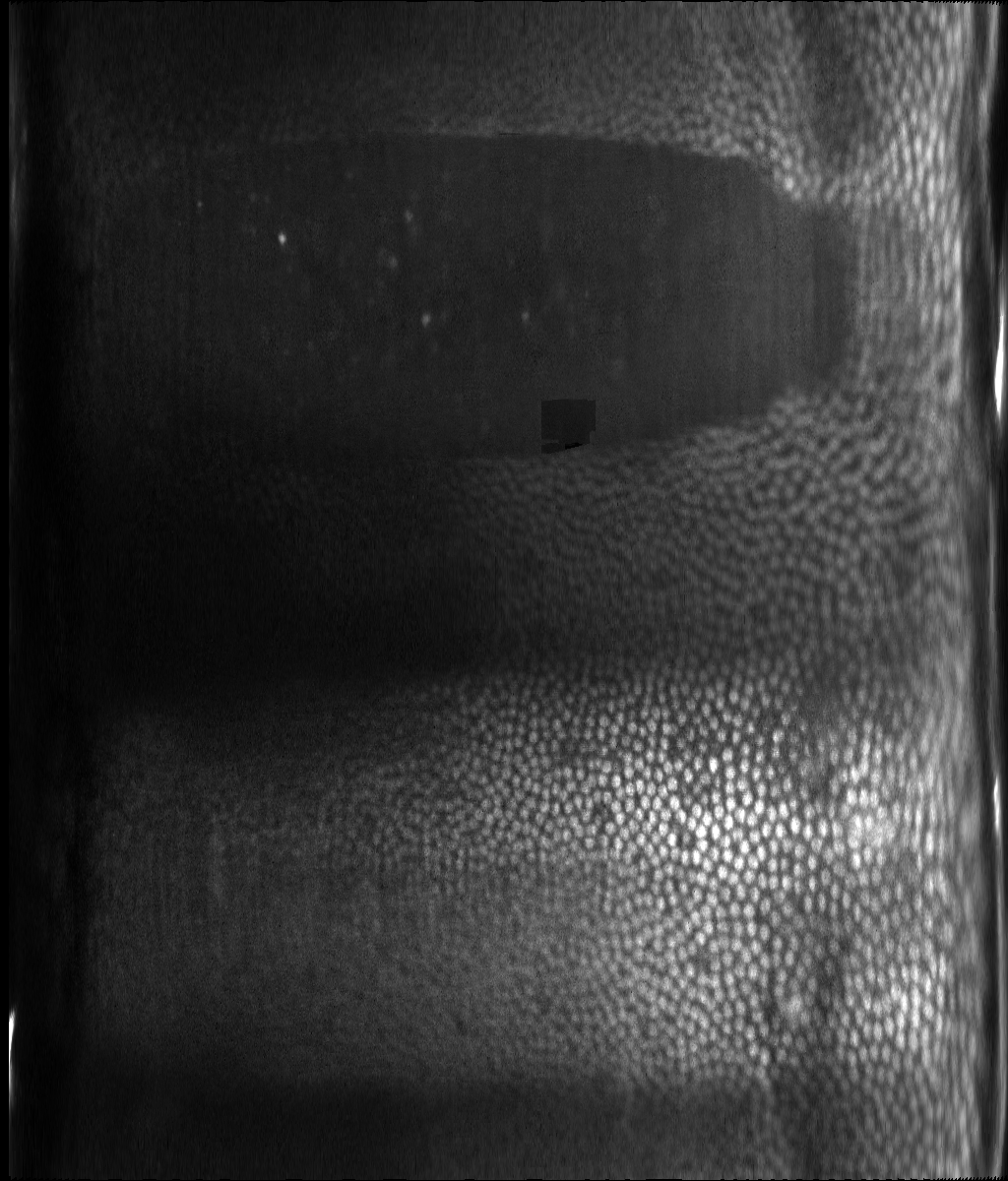

Supplement: Supplementary file 4 — Source Data for Figure 2 [file MSB-14-e8355-s003.zip › figure2-data/carpets_mCherry-sfGFP-Bcd/wt5_r_carpet.tif]

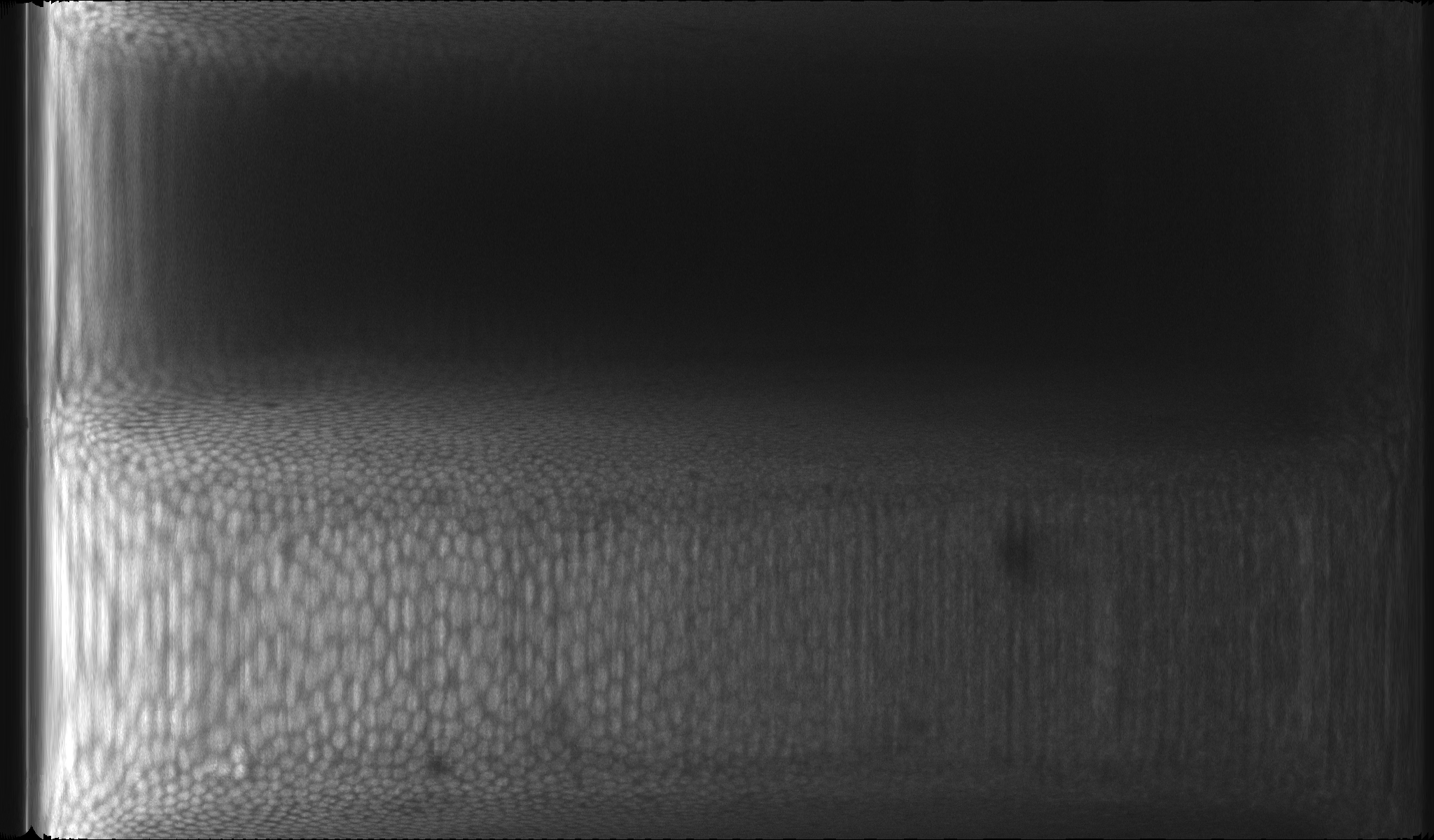

Supplement: Supplementary file 4 — Source Data for Figure 2 [file MSB-14-e8355-s003.zip › figure2-data/carpets_mCherry-sfGFP-Bcd/wt6_rot0_right_g_carpet.tif]

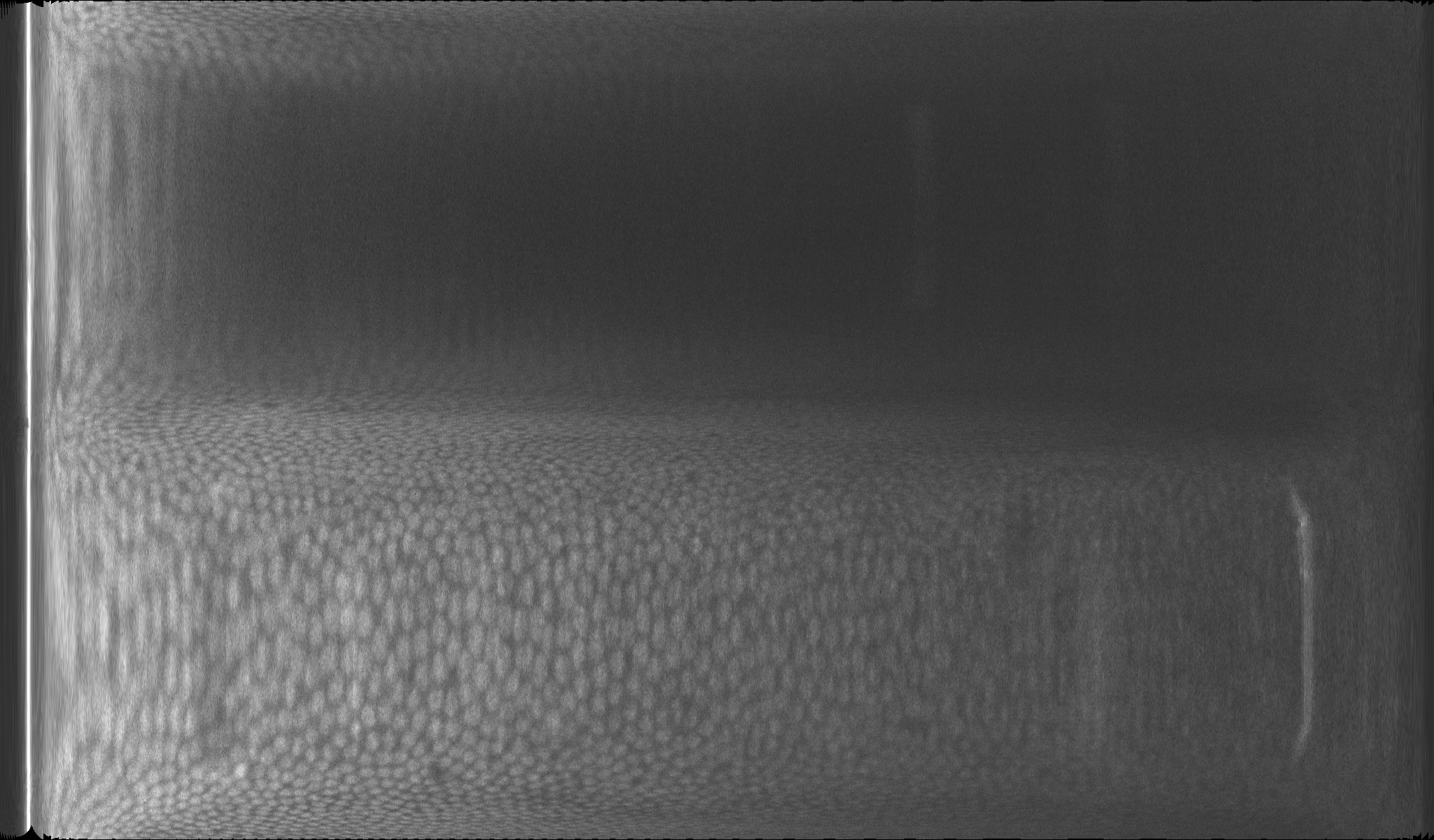

Supplement: Supplementary file 4 — Source Data for Figure 2 [file MSB-14-e8355-s003.zip › figure2-data/carpets_mCherry-sfGFP-Bcd/wt6_rot0_right_r_carpet.tif]

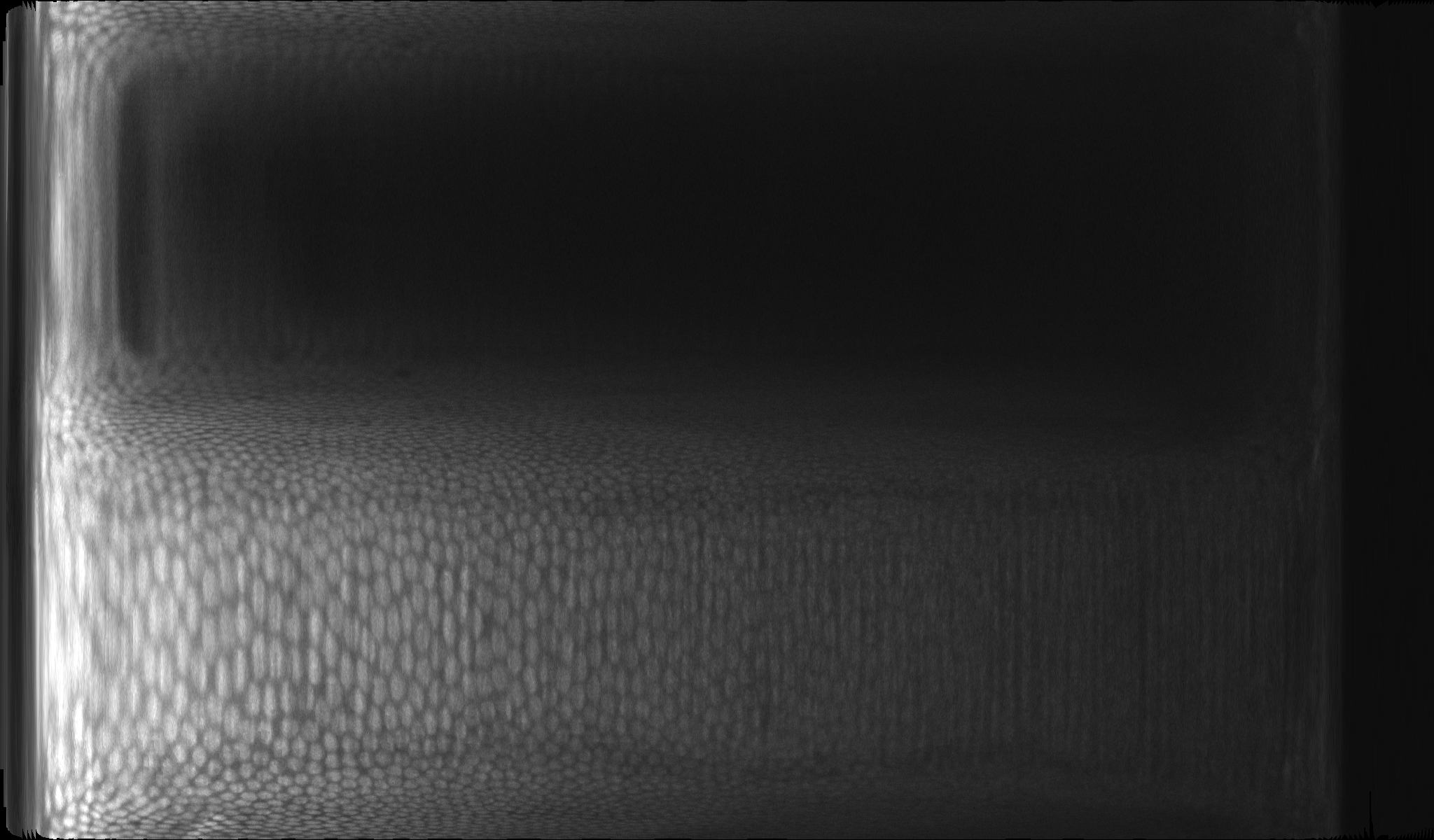

Supplement: Supplementary file 4 — Source Data for Figure 2 [file MSB-14-e8355-s003.zip › figure2-data/carpets_mCherry-sfGFP-Bcd/wt7_rot0_right_g_carpet.tif]

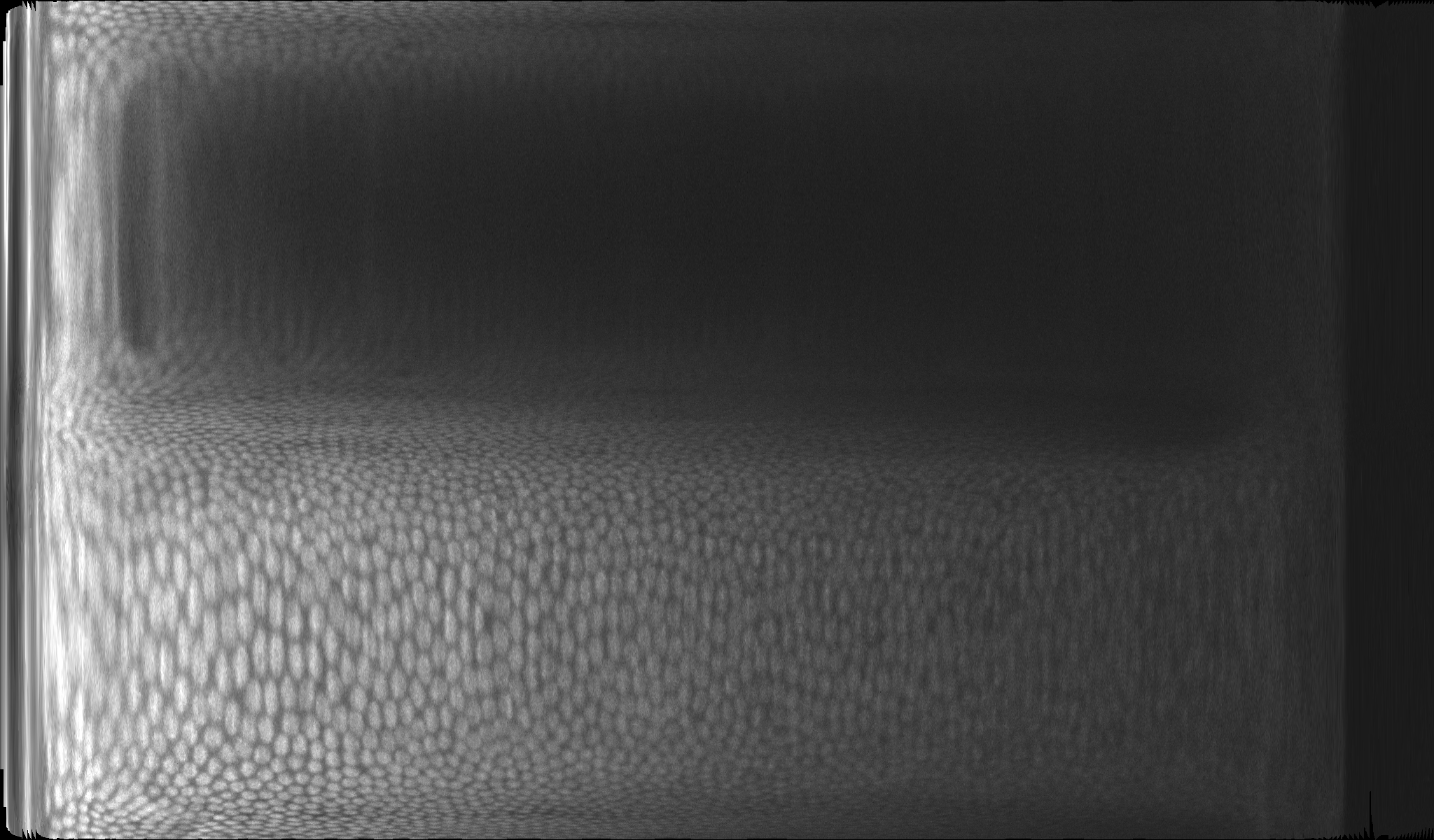

Supplement: Supplementary file 4 — Source Data for Figure 2 [file MSB-14-e8355-s003.zip › figure2-data/carpets_mCherry-sfGFP-Bcd/wt7_rot0_right_r_carpet.tif]

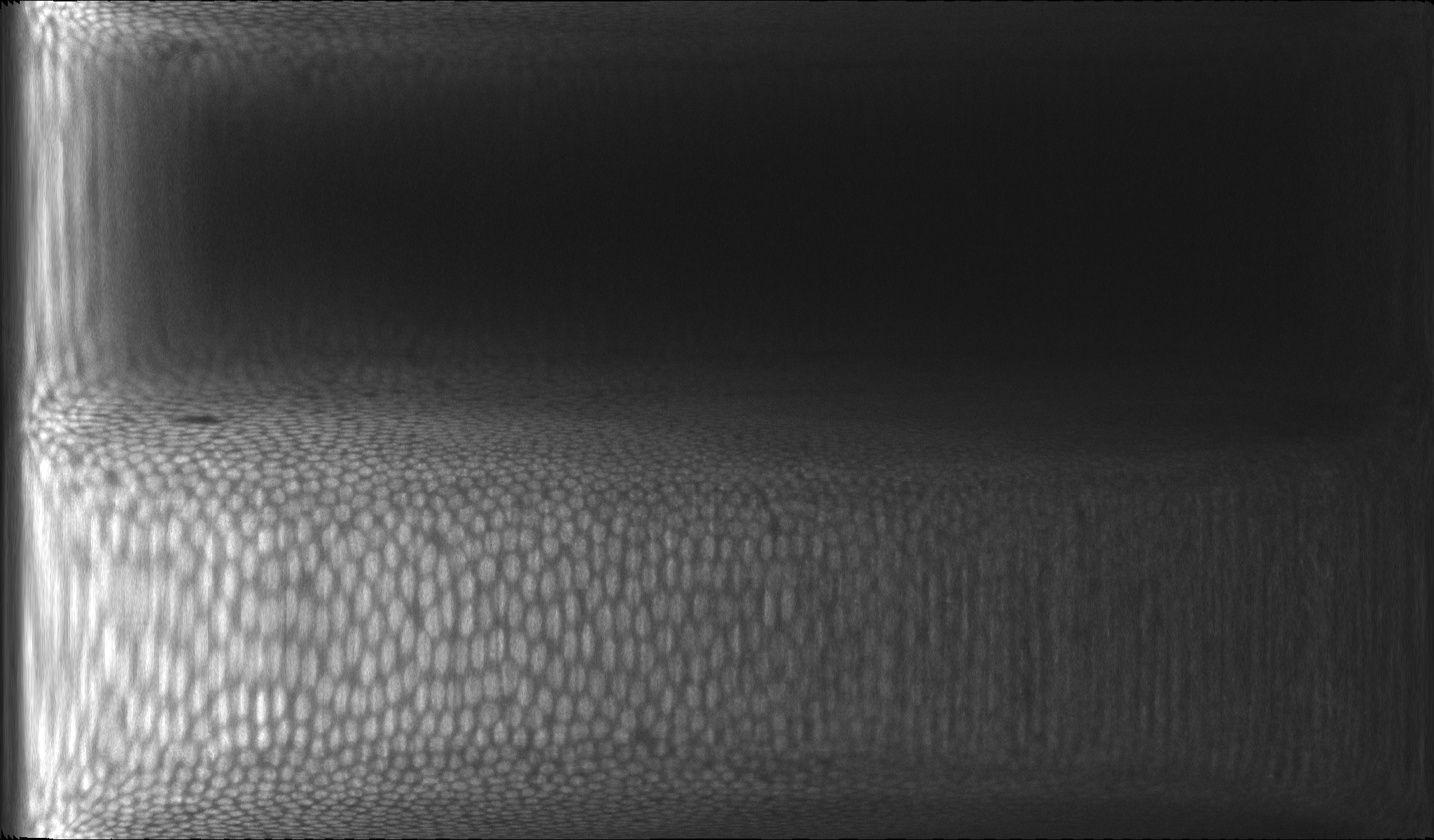

Supplement: Supplementary file 4 — Source Data for Figure 2 [file MSB-14-e8355-s003.zip › figure2-data/carpets_mCherry-sfGFP-Bcd/wt8_rot0_right_g_carpet1.tif]

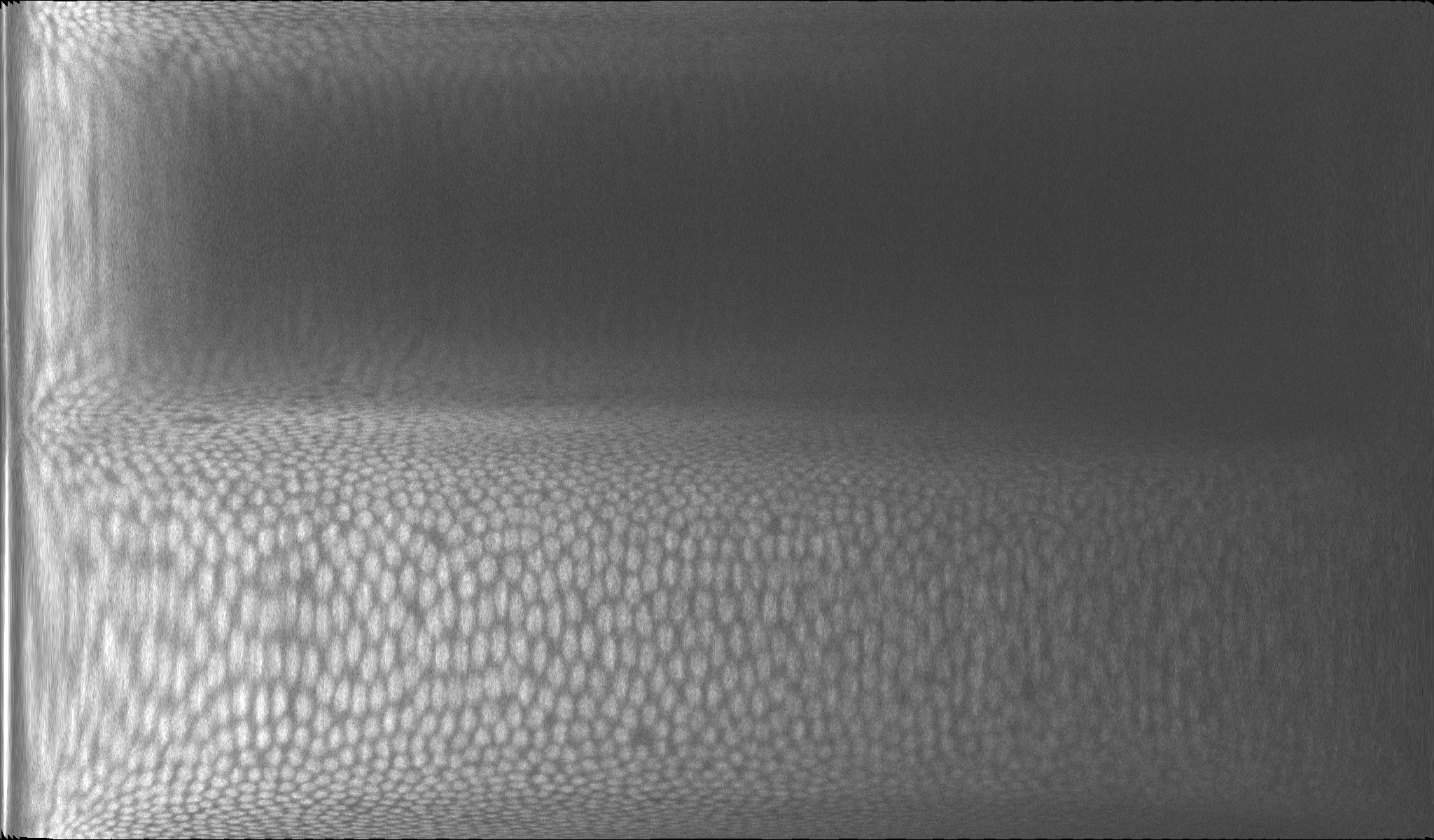

Supplement: Supplementary file 4 — Source Data for Figure 2 [file MSB-14-e8355-s003.zip › figure2-data/carpets_mCherry-sfGFP-Bcd/wt8_rot0_right_r_carpet1.tif]

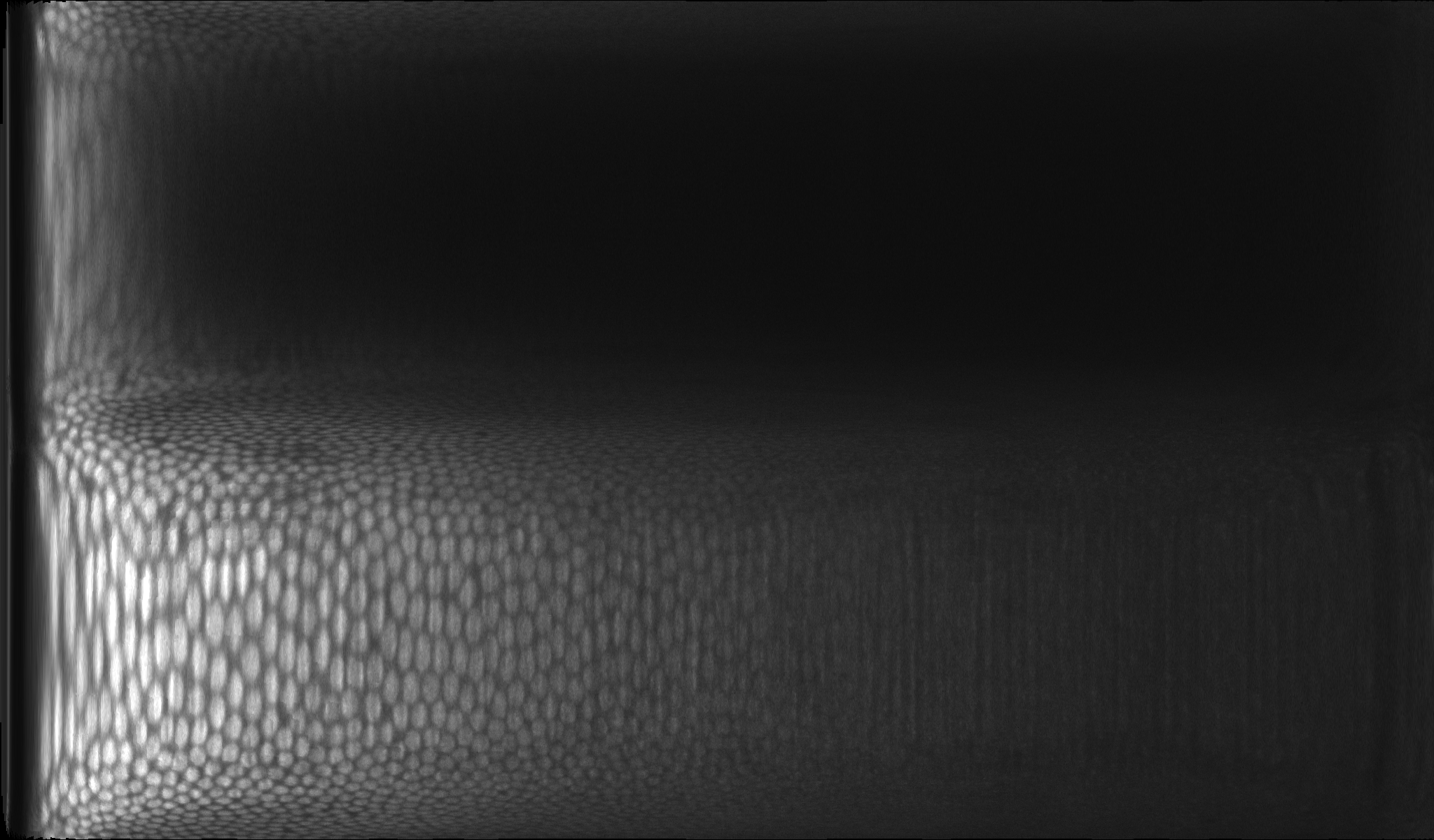

Supplement: Supplementary file 4 — Source Data for Figure 2 [file MSB-14-e8355-s003.zip › figure2-data/carpets_mCherry-sfGFP-Bcd/wt9_rot0_right_g_carpet2.tif]

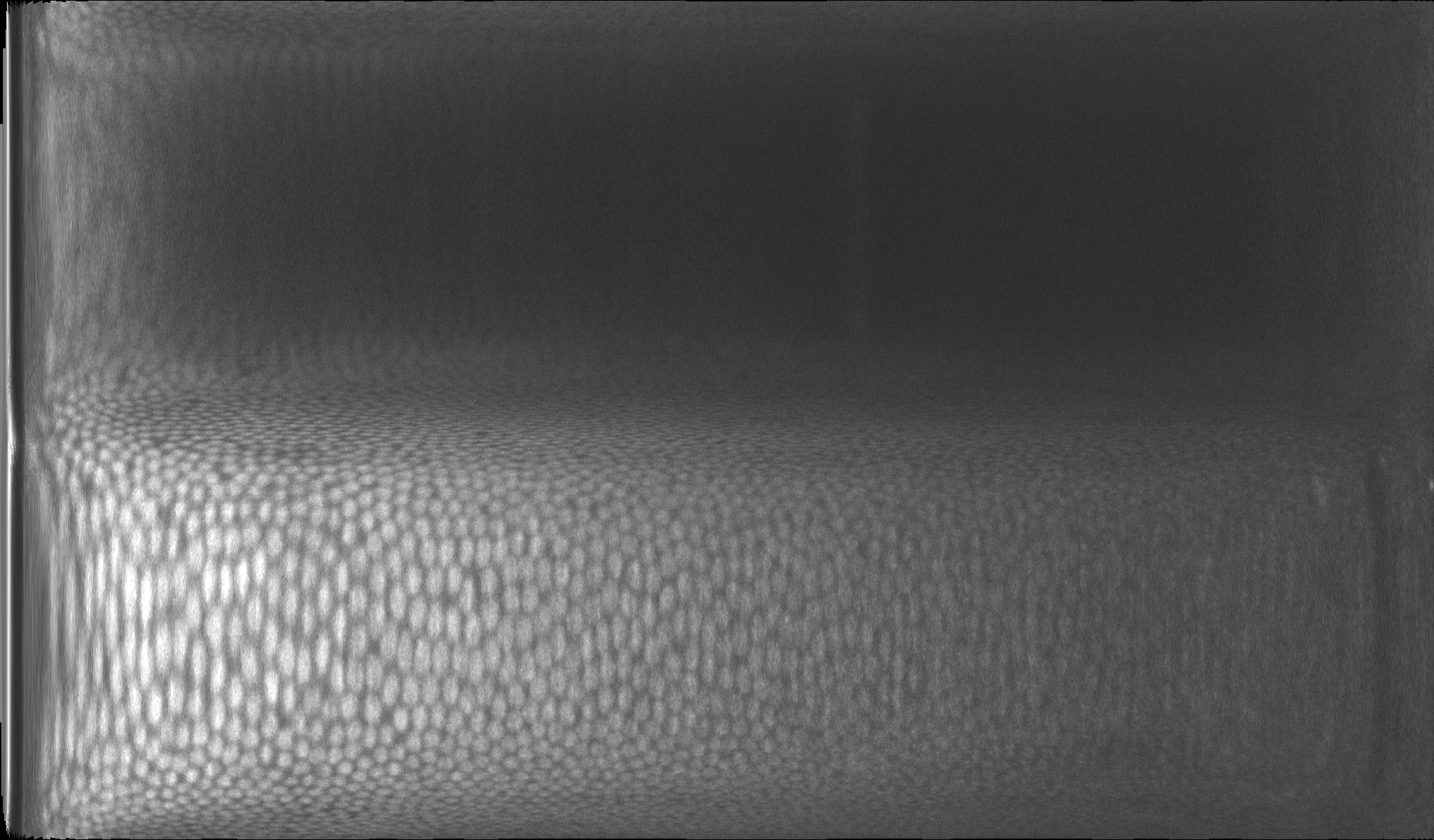

Supplement: Supplementary file 4 — Source Data for Figure 2 [file MSB-14-e8355-s003.zip › figure2-data/carpets_mCherry-sfGFP-Bcd/wt9_rot0_right_r_carpet2.tif]

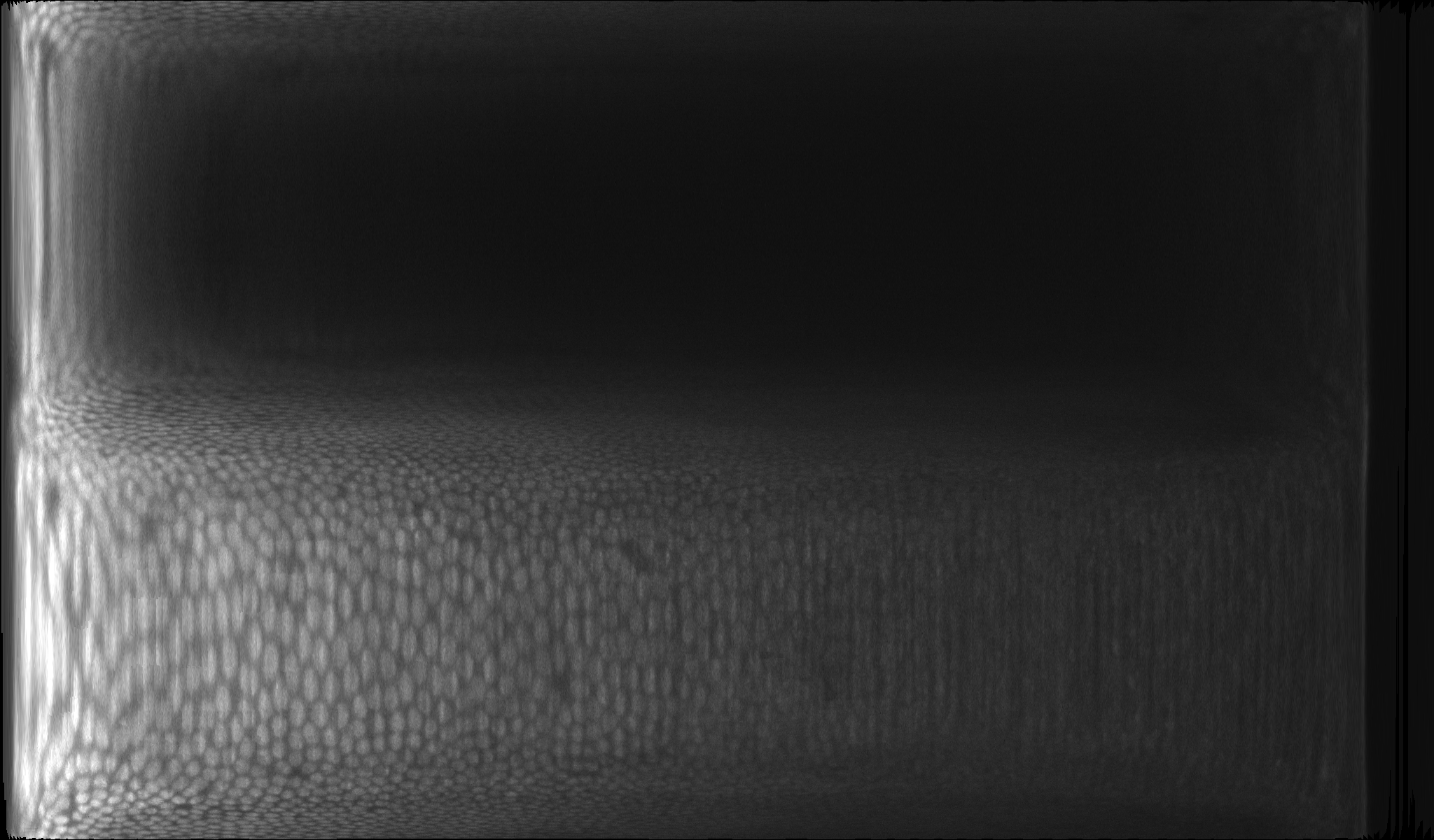

Supplement: Supplementary file 6 — Source Data for Figure 4 [file MSB-14-e8355-s005.zip › figure4-data/Fig4E_FSD-wt/carpets_mCherry-sfGFP-Bcd_deltaFSD/fsd10_rot0_right_g_carpet5.tif]

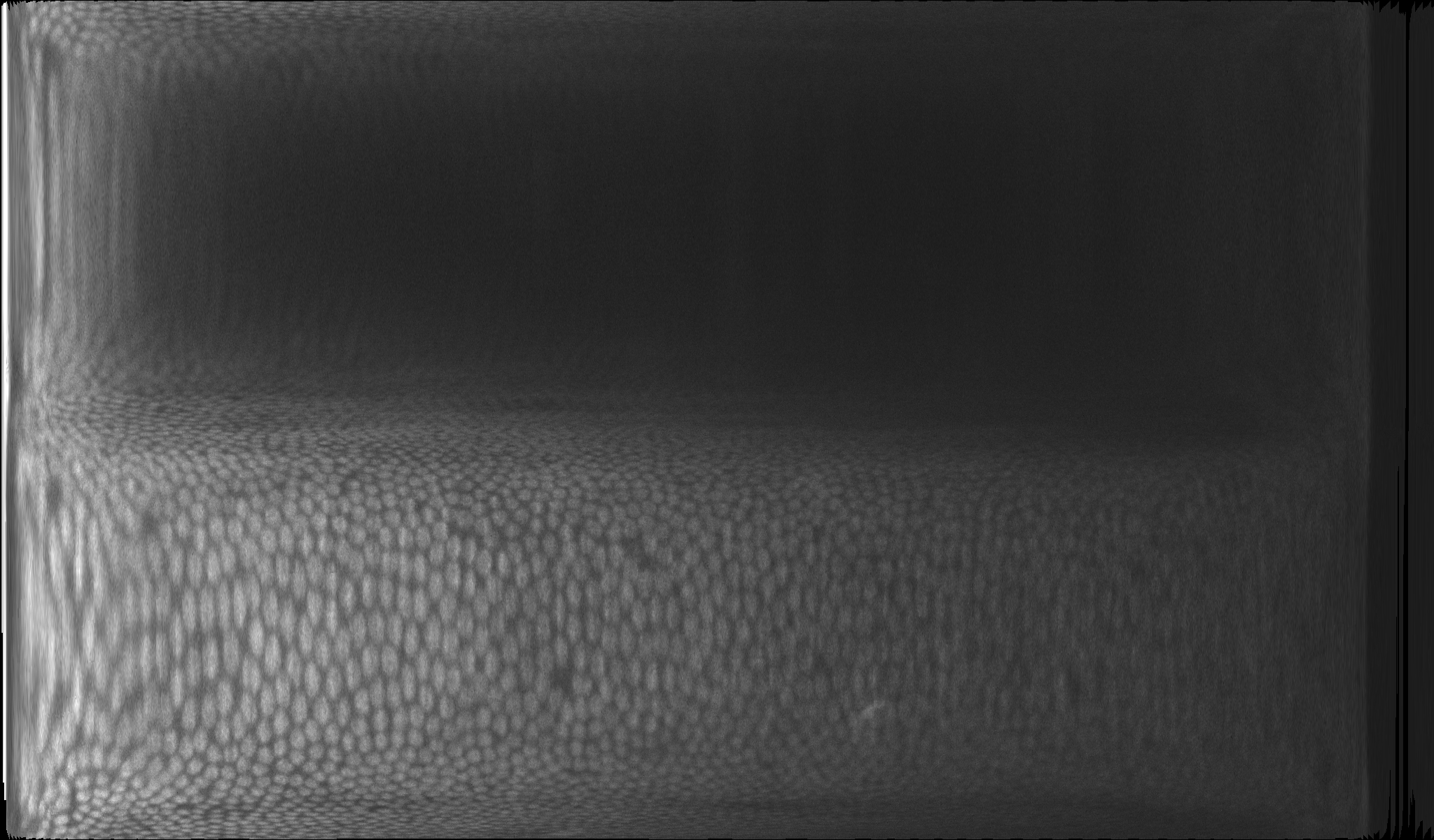

Supplement: Supplementary file 6 — Source Data for Figure 4 [file MSB-14-e8355-s005.zip › figure4-data/Fig4E_FSD-wt/carpets_mCherry-sfGFP-Bcd_deltaFSD/fsd10_rot0_right_r_carpet5.tif]

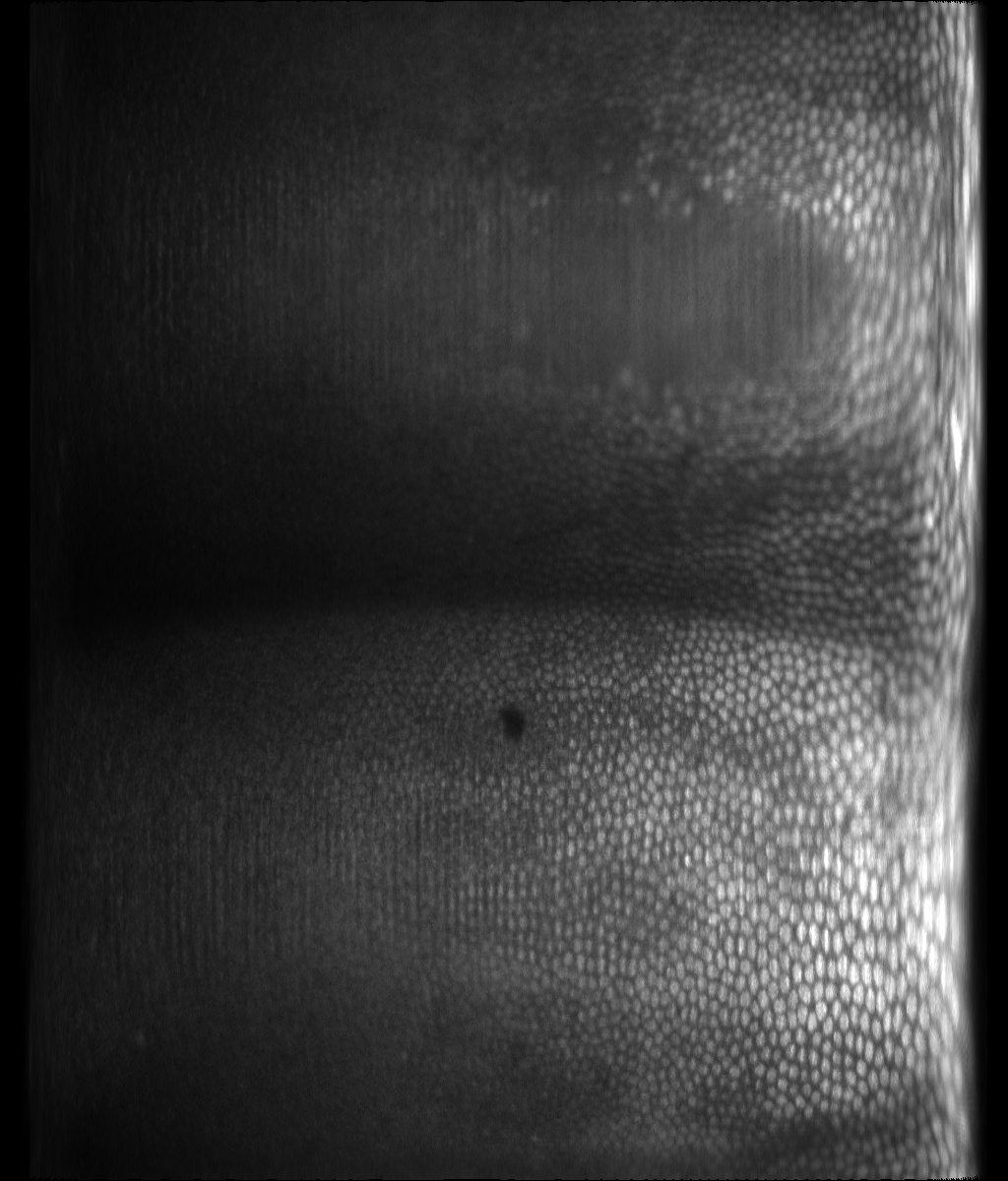

Supplement: Supplementary file 6 — Source Data for Figure 4 [file MSB-14-e8355-s005.zip › figure4-data/Fig4E_FSD-wt/carpets_mCherry-sfGFP-Bcd_deltaFSD/fsd2_g_carpet.tif]

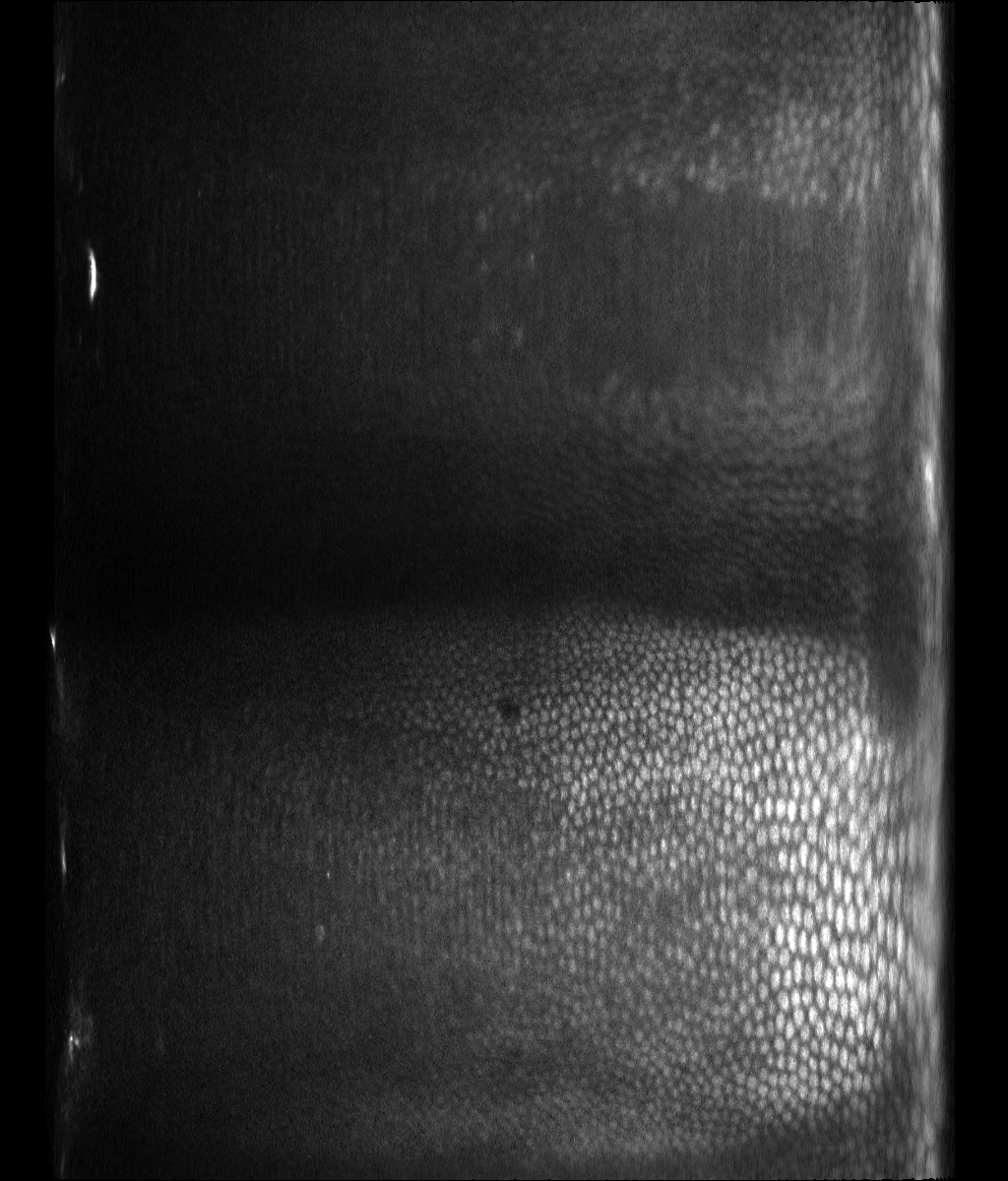

Supplement: Supplementary file 6 — Source Data for Figure 4 [file MSB-14-e8355-s005.zip › figure4-data/Fig4E_FSD-wt/carpets_mCherry-sfGFP-Bcd_deltaFSD/fsd2_r_carpet.tif]

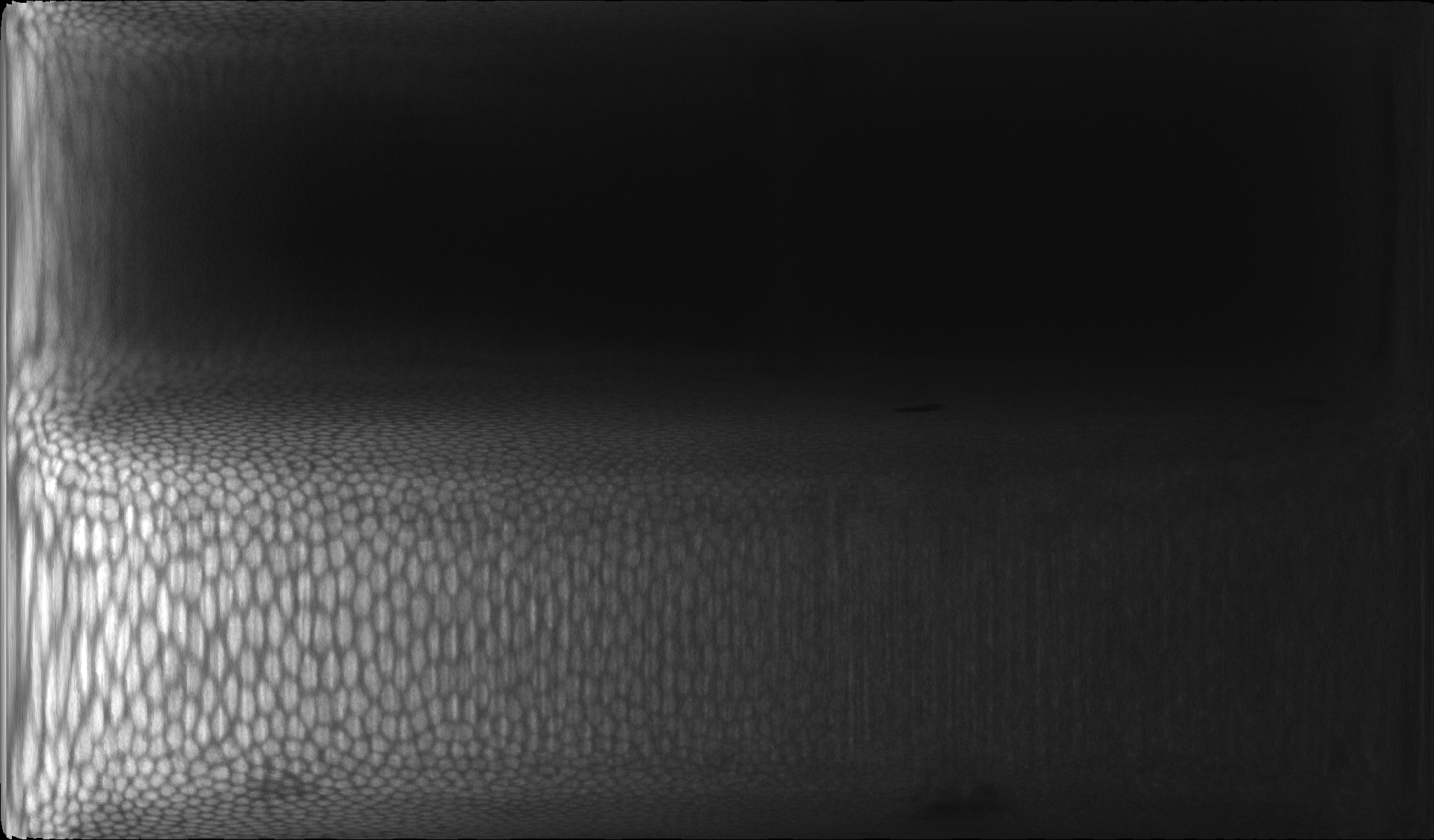

Supplement: Supplementary file 6 — Source Data for Figure 4 [file MSB-14-e8355-s005.zip › figure4-data/Fig4E_FSD-wt/carpets_mCherry-sfGFP-Bcd_deltaFSD/fsd6_rot0_right_g_carpet8.tif]

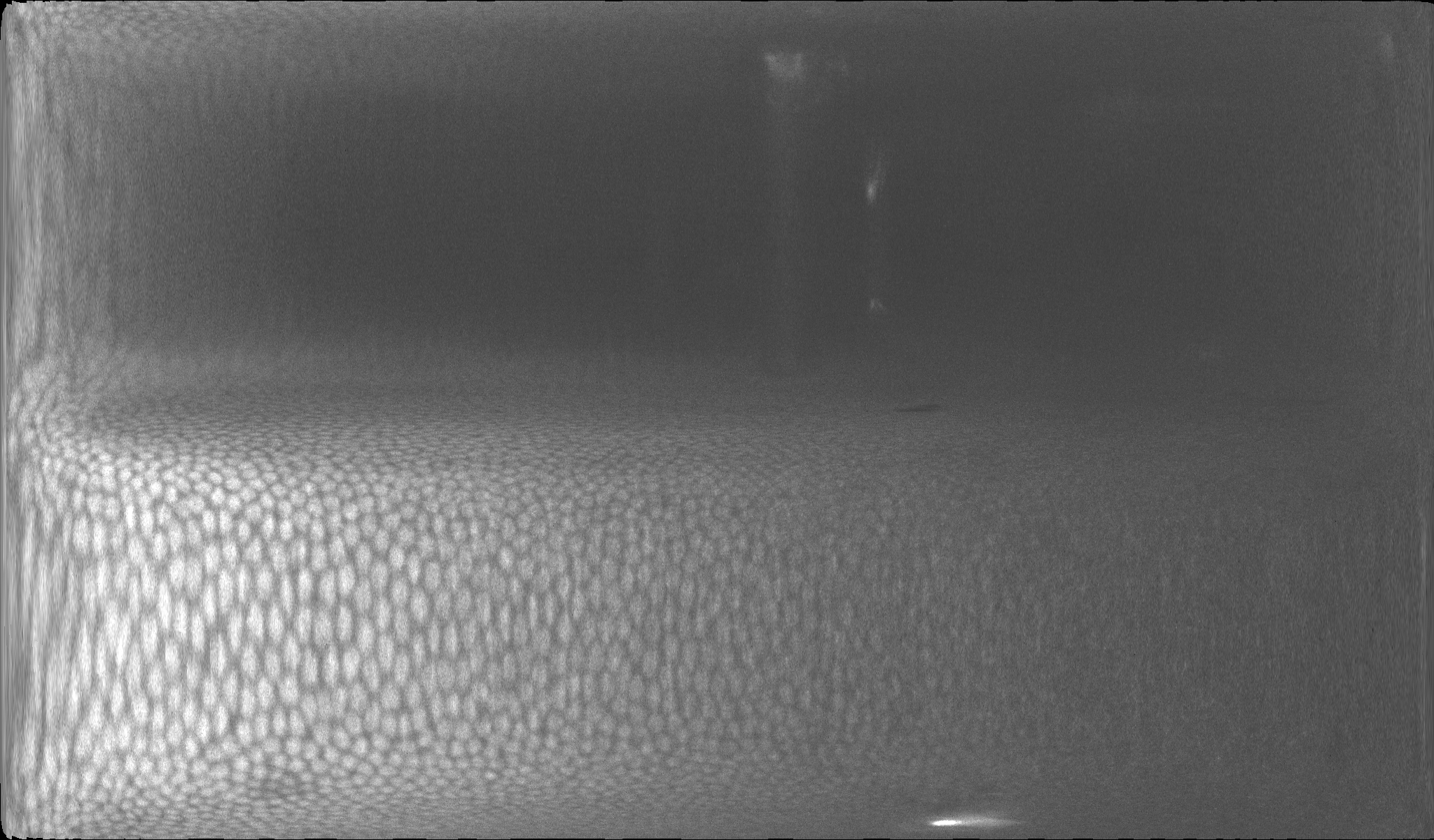

Supplement: Supplementary file 6 — Source Data for Figure 4 [file MSB-14-e8355-s005.zip › figure4-data/Fig4E_FSD-wt/carpets_mCherry-sfGFP-Bcd_deltaFSD/fsd6_rot0_right_r_carpet8.tif]

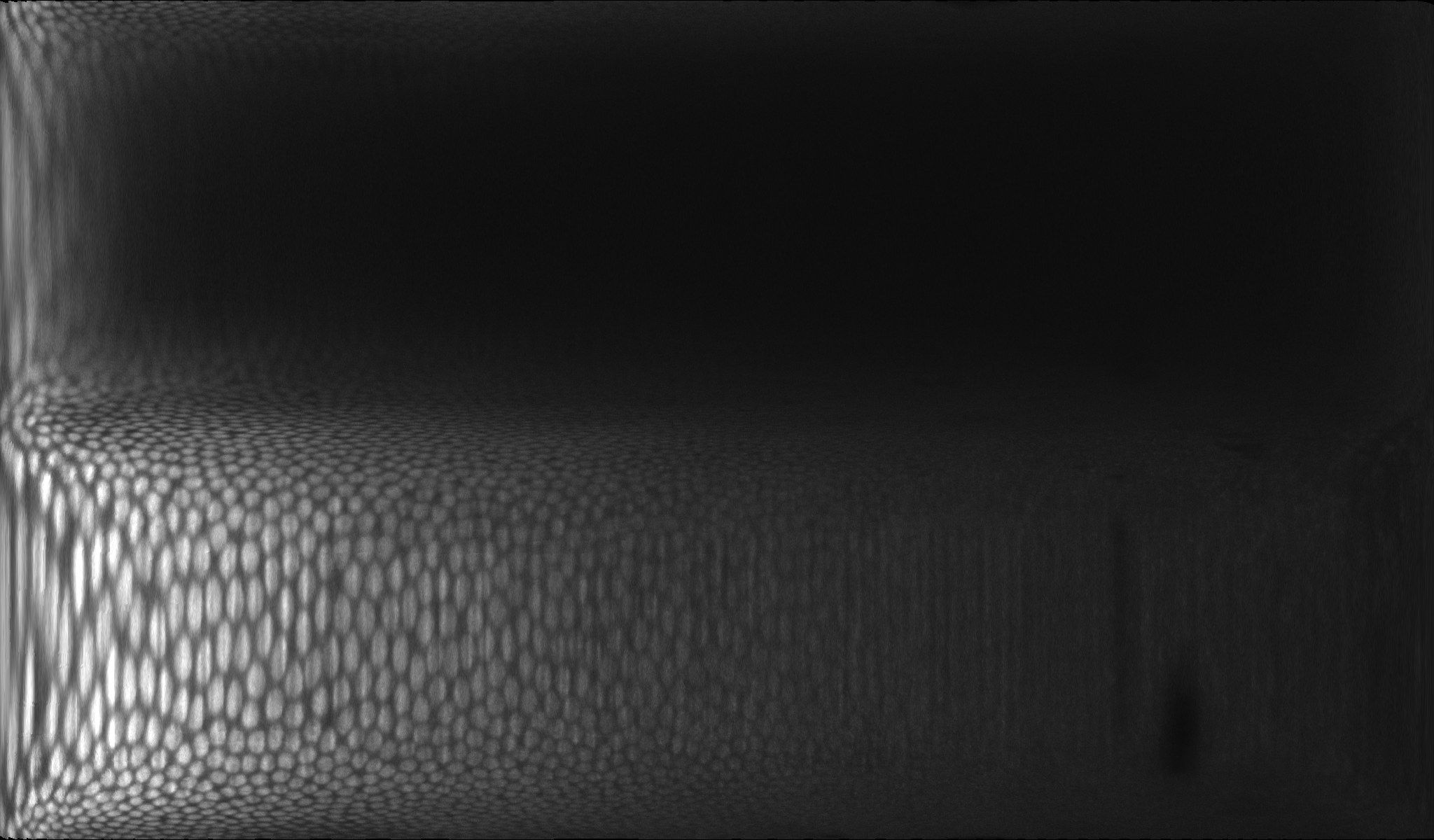

Supplement: Supplementary file 6 — Source Data for Figure 4 [file MSB-14-e8355-s005.zip › figure4-data/Fig4E_FSD-wt/carpets_mCherry-sfGFP-Bcd_deltaFSD/fsd7_rot1_right_g_carpet1.tif]

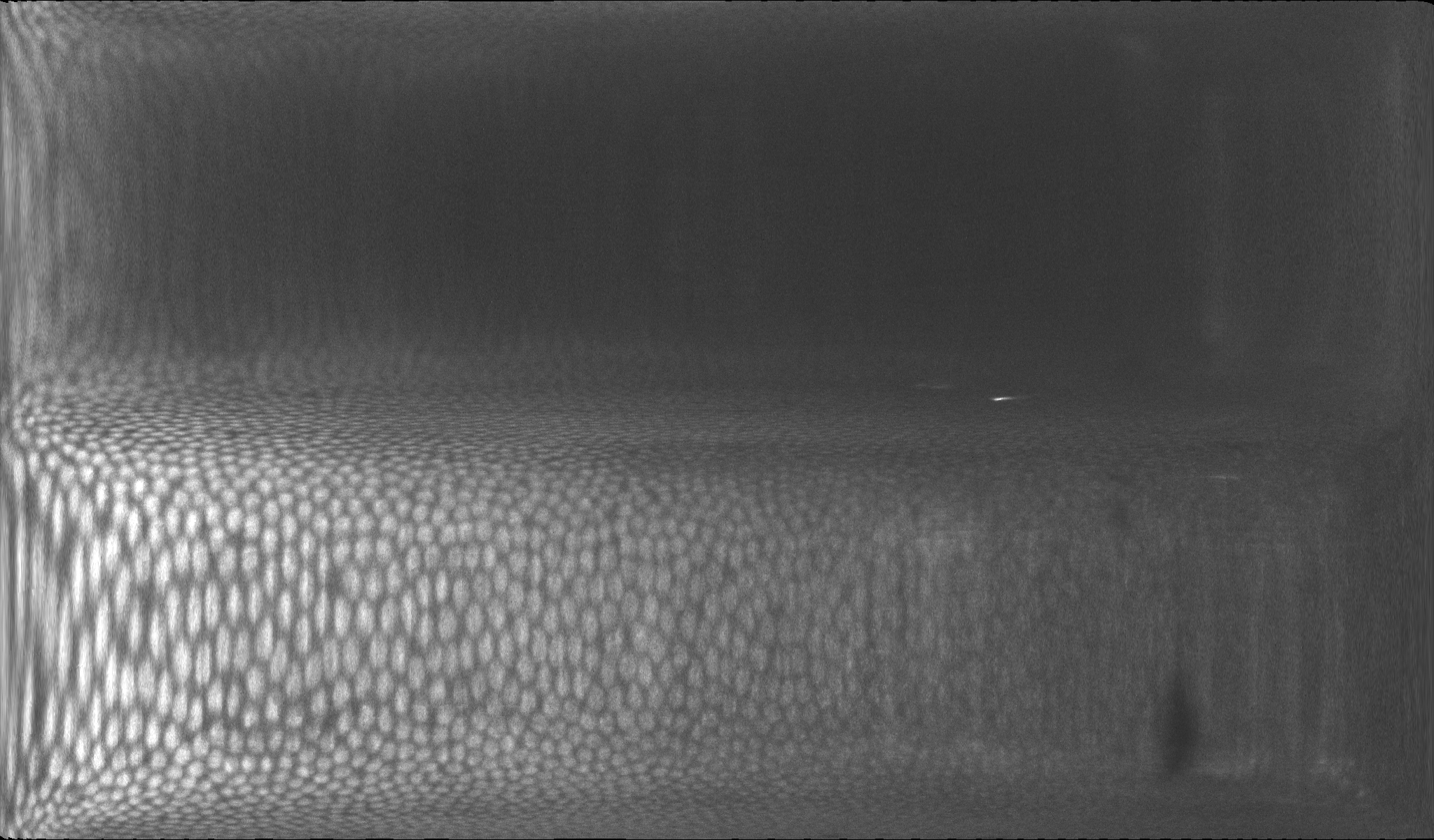

Supplement: Supplementary file 6 — Source Data for Figure 4 [file MSB-14-e8355-s005.zip › figure4-data/Fig4E_FSD-wt/carpets_mCherry-sfGFP-Bcd_deltaFSD/fsd7_rot1_right_r_carpet1.tif]

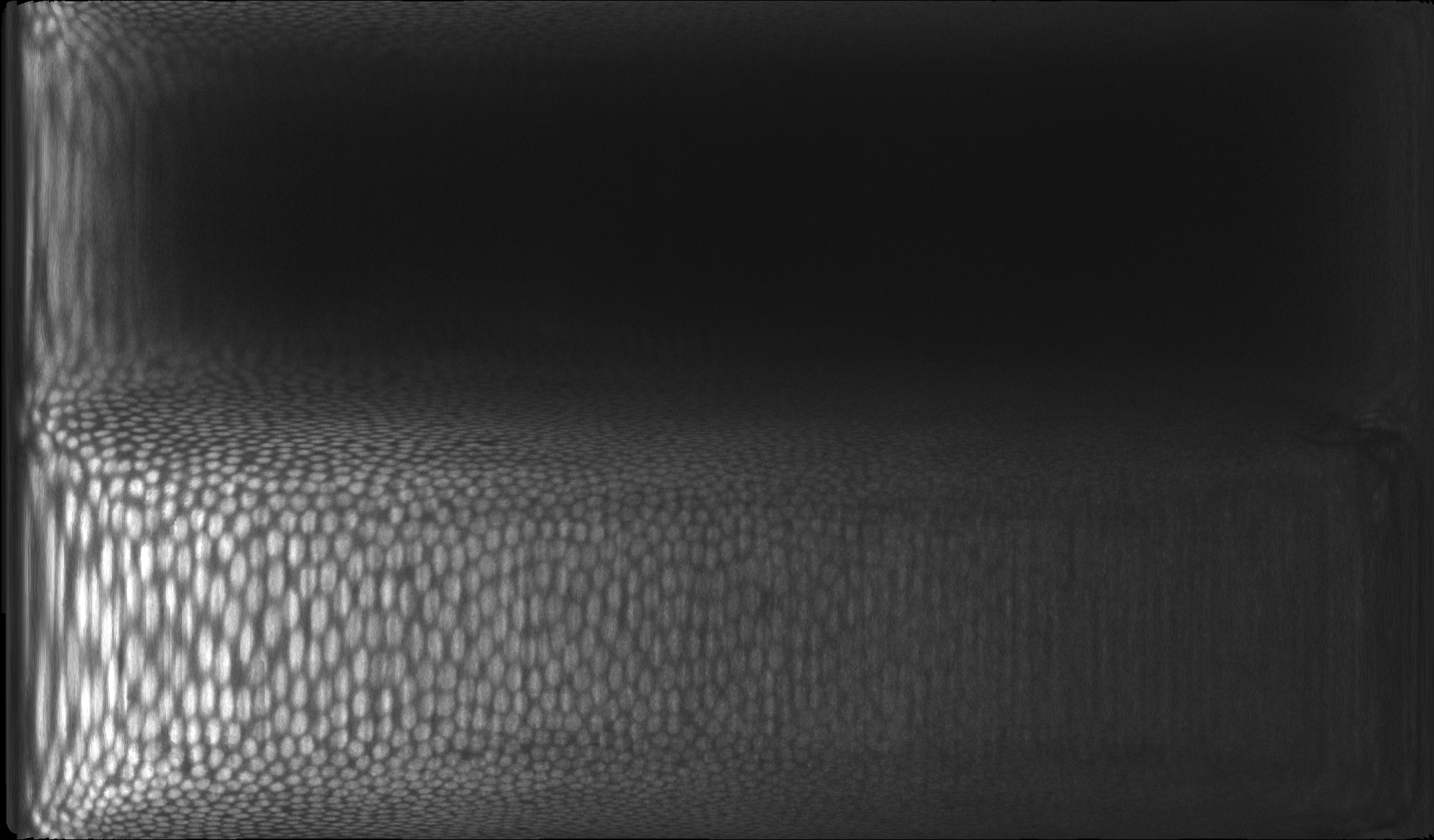

Supplement: Supplementary file 6 — Source Data for Figure 4 [file MSB-14-e8355-s005.zip › figure4-data/Fig4E_FSD-wt/carpets_mCherry-sfGFP-Bcd_deltaFSD/fsd9_rot0_right_g_carpet2.tif]

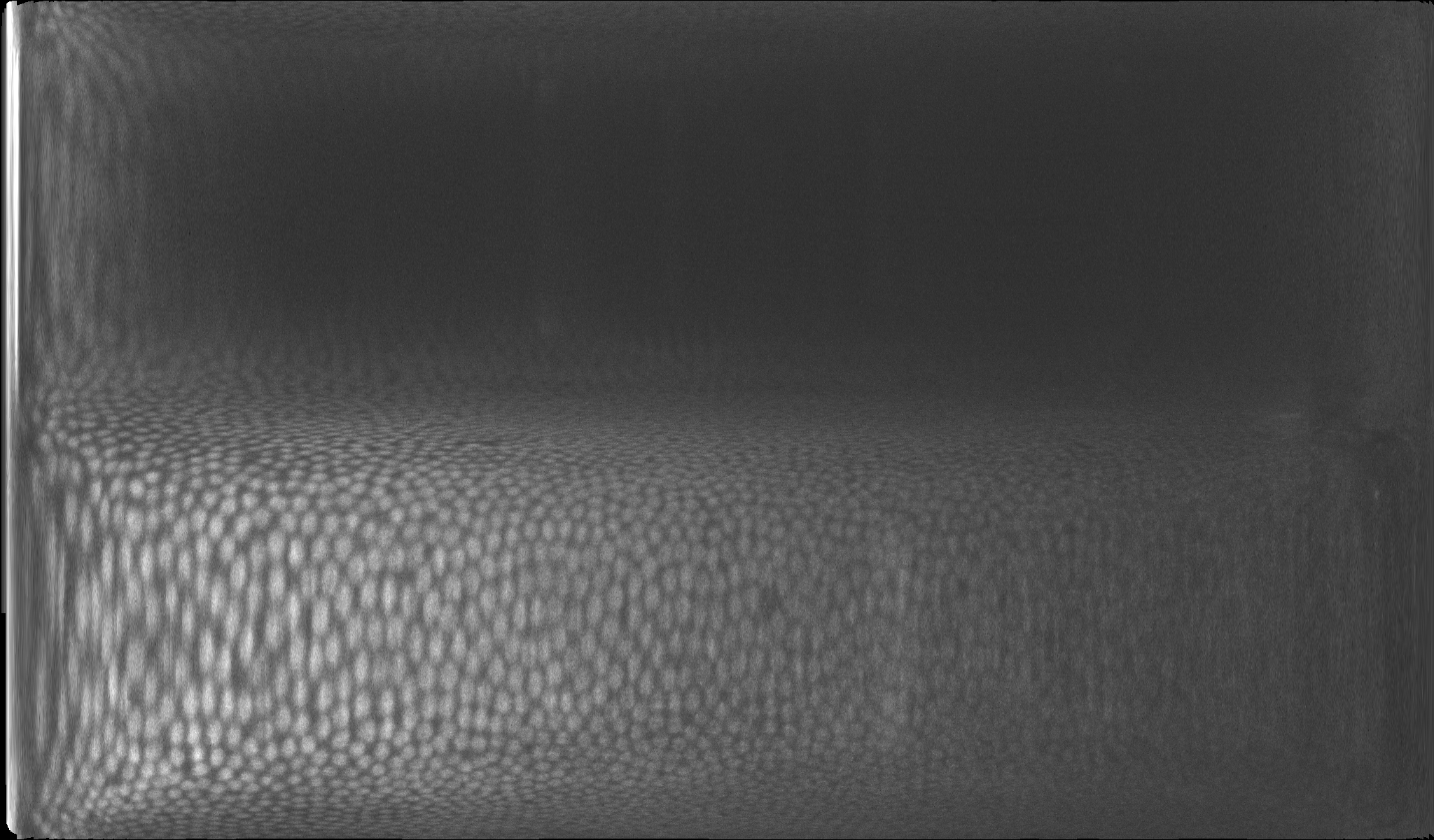

Supplement: Supplementary file 6 — Source Data for Figure 4 [file MSB-14-e8355-s005.zip › figure4-data/Fig4E_FSD-wt/carpets_mCherry-sfGFP-Bcd_deltaFSD/fsd9_rot0_right_r_carpet2.tif]
